# Mapping the Geometric Evolution of Protein Folding Motor

**DOI:** 10.1371/journal.pone.0163993

**Published:** 2016-10-07

**Authors:** Gaurav Jerath, Prakash Kishore Hazam, Shashi Shekhar, Vibin Ramakrishnan

**Affiliations:** Department of Biosciences and Bioengineering, Indian Institute of Technology Guwahati, Assam, India; Russian Academy of Medical Sciences, RUSSIAN FEDERATION

## Abstract

Polypeptide chain has an invariant main-chain and a variant side-chain sequence. How the side-chain sequence determines fold in terms of its chemical constitution has been scrutinized extensively and verified periodically. However, a focussed investigation on the directive effect of side-chain geometry may provide important insights supplementing existing algorithms in mapping the geometrical evolution of protein chains and its structural preferences. Geometrically, folding of protein structure may be envisaged as the evolution of its geometric variables: ϕ, and ψ dihedral angles of polypeptide main-chain directed by χ_1_, and χ_2_ of side chain. In this work, protein molecule is metaphorically modelled as a machine with 4 rotors ϕ, ψ, χ_1_ and χ_2_, with its evolution to the functional fold is directed by combinations of its rotor directions. We observe that differential rotor motions lead to different secondary structure formations and the combinatorial pattern is unique and consistent for particular secondary structure type. Further, we found that combination of rotor geometries of each amino acid is unique which partly explains how different amino acid sequence combinations have unique structural evolution and functional adaptation. Quantification of these amino acid rotor preferences, resulted in the generation of 3 substitution matrices, which later on plugged in the BLAST tool, for evaluating their efficiency in aligning sequences. We have employed BLOSUM62 and PAM30 as standard for primary evaluation. Generation of substitution matrices is a logical extension of the conceptual framework we attempted to build during the development of this work. Optimization of matrices following the conventional routines and possible application with biologically relevant data sets are beyond the scope of this manuscript, though it is a part of the larger project design.

## Introduction

The three dimensional native structure of a protein defies all concepts of aesthetics that nature generally tend to practice in its creation. However, these folded structures and the guiding principles of its construction is an important scientific problem, yet to be completely solved. The physical folding code, folding mechanism and a perfect algorithm that predicts the fold from sequence has been a matter of intense scrutiny for more than five decades now [1]. The scientific community has invested significant amount of time and resources in deciphering this Protein Folding code, because it is the key factor in the next level of drug discovery initiatives. Protein folding research community has grown in terms of scientific resources, ever since Anfinsen’s Thermodynamic hypothesis [2] and Levinthal’s paradox [3] have been established. Attempts to understand folding of sequences have resulted in two full-fledged disciplines in protein science viz. protein structure prediction and protein design [4]. Competition for Critical Assessment of protein Structure Prediction (CASP) has completed 11 versions so far, with varying degrees of success [5, 6] but with steady growth in its technical know-how. De novo Protein design started with rational design of small peptide segments developed to spontaneously self-folding units [7, 8]. In the last two decades, automated design methods took over, resulting in further advancement by creating functional units with amino acid sequences [9–11]. On the other hand, structural genomics initiatives are also gathering pace with entries in Protein Data Bank increasing more than ten folds in the last 15 years [12]. Even though significant progress has been made, structure elucidation of molecular targets for designing novel drugs is far from complete. Approximate solutions from various protein structure prediction tools are still very much operative and may continue for few more years at least.

In simple terms, solution to the protein folding problem is the ability to predict its structure from sequence. Researchers engaged in structure prediction are attempting this objective by alignment of amino acid sequences. On the other hand, for protein design, we may require an optimum sequence solution for a given template. Since the number of distinct folds for more than hundred thousand structures in PDB is only about 1500, multiple sequence solutions are possible for a given fold. Therefore, protein design is a more tractable problem than folding. Rotamer libraries by Dunbrack [13], Richardson [14] and studies by Pal *et al*. [15] were significant attempts in quantifying side-chain geometries and therefore serve as the most crucial element in automated design tools. However, mechanistic investigations so far, have conveniently under-played the specific role of side-chain geometry and its effect on folding.

Protein folding research was largely concentrating on the thermodynamics of folding in its early years of research, principally because of the lack of sufficient data to study its kinetics. Efforts to decipher the sequential events in a typical folding process have gained momentum in last couple of decades [16–18]. Molecular dynamics simulations [19–21], graph based unfolding schemes [22] and various other ‘reductionist’ approaches have contributed to the understanding of intermediate structures in the route of evolution of a typical hetero-polymeric amino-acid chain to a fully functional three dimensional fold [23]. Basically, the only difference between two proteins is the difference in its length and amino-acid sequence. Amino-acid sequence is typically its side-chain sequence. Therefore, it is logical to assume that the chemistry of side-chain or its combinations determine the fold. Almost all sequence alignment programs make use of a scoring scheme based on a substitution matrix [24–27]. The similarity or differences in these matrices are calculated based on odds ratio rooted either on its chemistry or statistics or both. The general consensus is that intra-molecular interactions and combinations of it in varying degrees, arising from the main-chain and side-chain of an amino-acid sequence *initiate* the motion of protein chain, *propagation* of that motion and its *culmination* in a unique fold. One important missing link that may have to decipher is the trace of geometrical events of this protein folding motor. We make an attempt to trace the path of the geometry of this motion, by following the dihedral angle distribution during the early events of protein folding. Other acceptable means for such an investigation would be to employ Molecular Dynamics Simulation methods generating and ensemble and attempt to map the movements. However, MD simulations can be performed only for a handful of structures and not an extensive and comprehensive dataset used for this investigation.

### Rotors and Stators of the Folding motor

Structure of a protein chain can be geometrically mapped by its backbone dihedral angles ϕ and ψ. Side-chain dihedral angles χ_1_ and χ_2_ and their contributions were more a concern for protein designers and crystallographers, while they optimize side-chain packing of a given fold. Allowed regions of ϕ and ψ combinations in a Ramachandran plot is very limited, and therefore structural diversity of protein secondary structure is also limited to α-helices, β-sheets and connecting segments. In 2009, Cole and Bystroff proposed the phone cord model for helix formation and favouring of right handed helices over left handed ones in the crossovers of βαβ super-secondary structures [28]. Phone cord model highlights the creation of a torque on two ends of the helix during its formation, which may either be relieved by the rotation of one end of the chain or by its pivoting. Protein polymeric chain does not behave like a random flight chain with un-restricted freedom of movement, instead is a stiff chain mainly due to the geometric restrictions of its dihedral angle rotations. Chain stiffness is a general term used to describe the degree of angular correlation between neighbouring bonds, and is often measured in terms of characteristic ratio. Characteristic ratio of a random flight chain is 1. Characteristic ratio calculated by P.J. Flory for polypeptide chains ranges between 9.0 and 9.5, mainly due to the poly L (or hypothetically poly R) stereochemistry of polypeptide chain [29]. This indicates that the folding of this stiff protein chain is a directed motorized process, with the motive force generated through non-bonded interactions and dispensed through dihedral angle motions.

**Fig 1:**
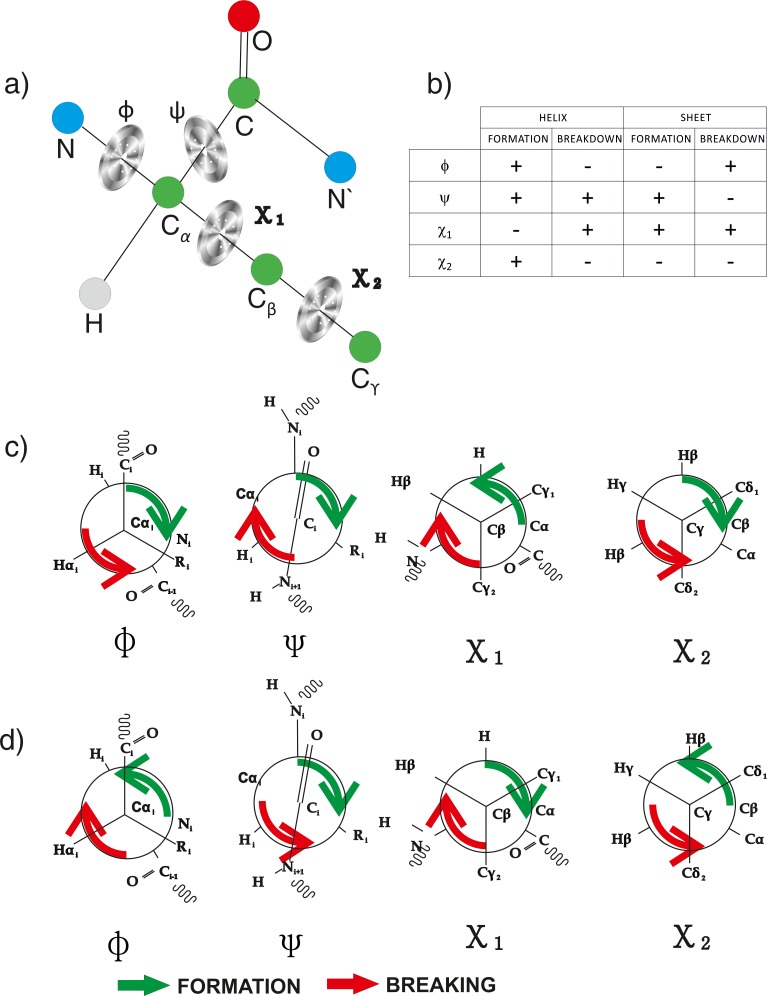
Functional rotors involved in structure formation: a) The four rotors representing four dihedral angles of an amino acid residue in a polypeptide chain. b) Orientation of four rotor motions during formation and breaking of helix and sheet with positive sign indicating right handed or clockwise direction and negative sign indicating left handed or counter clockwise direction. c) Rotational patterns of rotors during helix formation (green) and helix breaking (red) are shown d) Orientation of rotors resulting in the formation and breaking of sheeted structures.

A self-folding polypeptide chain may hence be assumed as a motor with four rotors ϕ, ψ, χ_1_, χ_2_, (Fig 1A) and all other elements being stators. The motive force in either direction of folding or unfolding, may come from the mutual dispensation of enthalpic and entropic forces within the chain, subject to reaction conditions. The necessary torque required to fold a polypeptide chain, may come from within, and thermodynamics of folding has already been studied extensively. We were curious to find out how this torque is manifested sequentially such that the chain can move into helix and sheets and eventually assume a unique fold for a given sequence. It is safe to assume that this torque is manifested through dihedral angle movements. Therefore, we attempted to map the sequential geometric events of folding by following the movements of four dihedral angles during secondary structure formation and breaking. A post-secondary structure motion of this motorized process culminating in a fold, is unique for a given sequence and hence cannot be brought to a common platform.

## Materials and Methods

Our statistical analysis is based on a 22,997 strong non-redundant protein dataset available on the PISCES server [30], consisting of 14,556,807 amino acid residues, altogether forming 406,799 helices and 576,240 β-sheets. The set of non-redundant protein structures as provided in the PISCES server were downloaded from PDB and dihedral angles were calculated in the given structures. The output was segregated into three groups: β-sheets, α-helices and others as per their main chain dihedral angle distributions. To these groups, we also added the flanking amino acids of each secondary structure, stretching to three amino acids on either side. Further, each of these lists were reduced into a 37 X 37 residue frequency matrix with each value in the matrix representing the number of amino acids present in a 10^0^ by 10^0^ angle grid, as per the value of their dihedral angles. Such matrices were constructed for every flanking region and secondary structure positions for various combinations of dihedral angles. After the construction of such matrices, we plotted them as contour maps in order to analyse the quantitative distribution of amino acid main and side chain dihedrals in protein structures.

## Results

### Dataset

Our logical deductions are based on the statistical analysis of a non-redundant protein data set of about 23,000 protein structures from Dunbrack’s library [30]. We extended the broad conclusions of this study by generating three substitution matrices for protein sequence alignment. These matrices were compared with the standard ones and their relative performances are elaborated in the latter sections of this manuscript.

In an attempt to map the early events of folding and understanding the driving force responsible for the sequential events, we have classified the dihedral angles of amino acids preceding and succeeding the basic secondary structure elements—helices and sheets. Therefore, we have separately classified amino acids at H-3, H-2, H-1, H, H+1, H+2 and H+3 positions each getting into and leaving the helical structures. Similarly, we have S-3, S-2, S-1, S, S+1, S+2 and S+3 for sheets. We have taken the whole sheet segment and not just a strand for better clarity and precision of data.

### Relative motions of secondary structure forming dihedral angle rotors

We follow the motions of dihedral angles by mapping the dihedral angle basins of three residues going into the secondary structure, and three residues leaving it as described in the previous paragraph. We observe that, the behaviour of polypeptide chain dihedral angle rotors are analogous to an enigma machine with the information regarding targeted secondary structure is enciphered in it. Rotor orientations are distinctly different for different secondary structures. We observed that helix formation is a result of concerted motion of main chain and side-chain rotors, in varying degree of participation and differential mode of rotations (Fig 1A). We first evaluated the orientation of χ_1_ and χ_2_ dihedral angles, because they are successive rotors of the same branch. We had an interesting observation that χ_1_ and χ_2_ dihedral angles are counter-rotating while formation and breaking of secondary structures under all conditions (Fig 1B). A more interesting observation is that χ_1_ is also counter-rotating with ϕ while formation and breaking of the helix. Therefore, folding-unfolding machine has these three rotors counter-rotating, while forming any secondary structure. The next important observation is that ψ dihedral angle rotor is always left handed in its orientation except while breaking of sheet. This further indicates that ψ dihedral angle do not have a decisive role in the evolution and choice of secondary structure formation (Fig 1B–1D).

#### Helix formation and breaking

**Fig 2:**
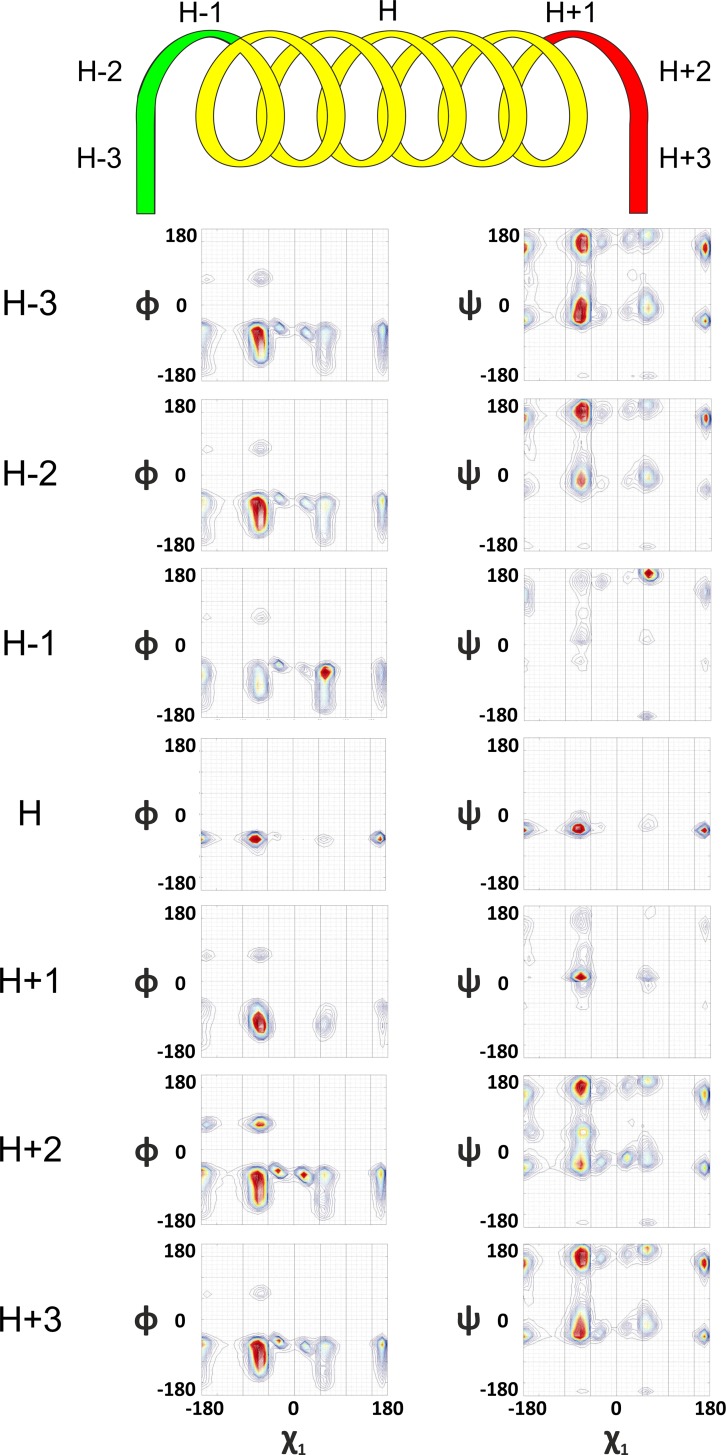
Helical structure formation and its breaking. H-3 to H basin shifts indicate formation of helix as a result of left handed rotation of the χ_1_ rotor and right handed rotation of ϕ by 80° while helix breaking the rotors assume reverse orientations. The ψ rotor maintains its right handed rotation throughout helix formation and breaking. See also Figures A1-A4 in S1 File.

Helix is formed when χ_1_ rotor rotates to the left. ϕ dihedral angle rotor complements in counter direction by rotating to the right by 80°. While helix breaking, χ_1_ and ϕ rotors assumes reverse orientation. χ_2_ dihedral angle maintains its mandate of counter-rotation with χ_1_ rotor, during formation and breaking. The ψ dihedral rotor maintains a right handed rotation during both helix formation and breaking (Fig 2).

#### Sheet formation and breaking

**Fig 3:**
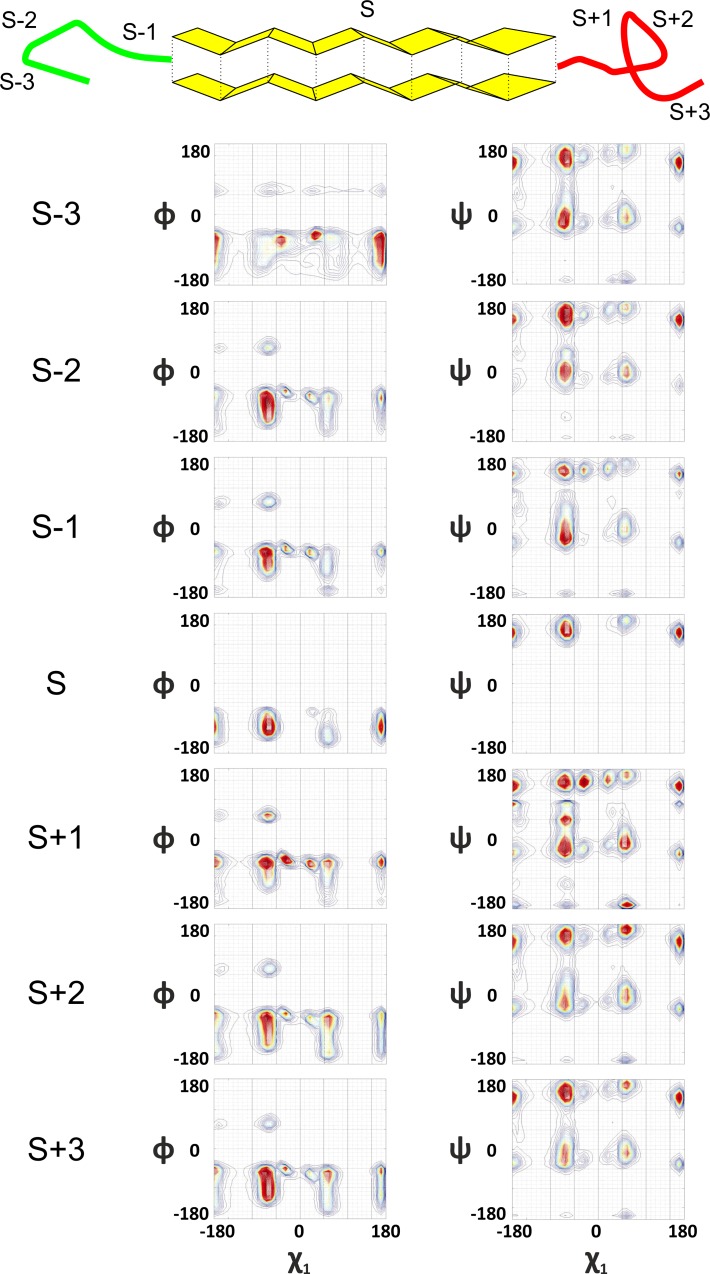
Formation of extended sheeted structures and their breaking. S-3 to S basin shifts indicates formation of sheet as a result of right handed movement of the χ_1_ rotor and complimenting counter rotation of ϕ; ψ rotor moves in the counter-direction of the ϕ rotor throughout formation and breaking of sheet. See also Figures A1-A4 in S1 File.

Sheet or a beta strand is formed when χ_1_ rotor rotates (clock-wise) to the right unlike helix. ϕ dihedral angle rotor complements in counter rotating direction by rotating to the left full circle 360^0^ (Fig 3). χ_2_ dihedral angle maintains its mandate of counter-rotation with χ_1_ rotor, during formation and breaking of sheet as well. Both ϕ and ψ rotate in counter direction while breaking. Rotations of ϕ in counter directions, while formation and breaking is hence, a common feature for both helices and sheets.

Therefore, trace of dihedral angle geometry tells us an interesting story, untold so far to the best of our knowledge, which can be summarized as follows: i) Geometric evolution of helix and sheet are distinctly different and in most cases opposite; ii) Rotor motions, through which these differential evolution patterns are orchestrated are unique for a given secondary structure; and (iii) the resultant secondary structure formation is a result of this differential rotor motions (Figs 1, 2 and 3 and Figures A1-A4 in S1 File), though the constitution of differential driving forces for these secondary structures are still unclear.

### Dihedral angle rotor patterns and amino-acid choices

**Fig 4:**
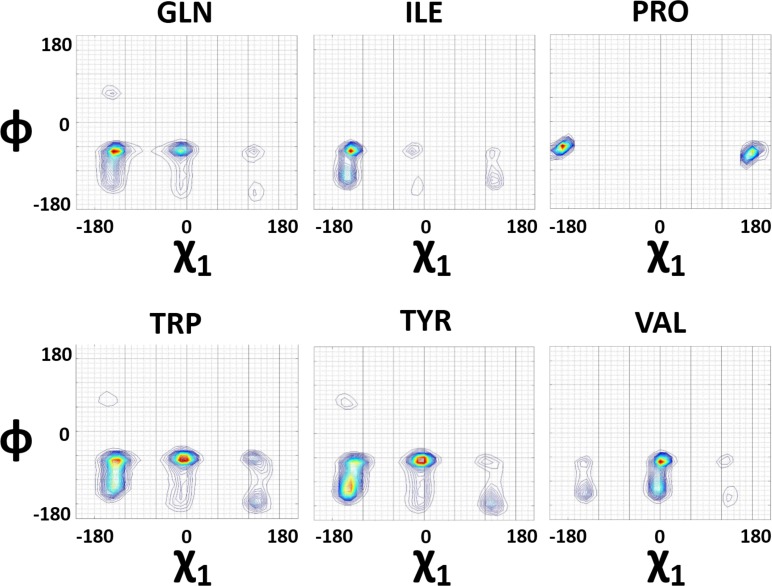
Differential distribution of side-chain and main-chain rotors among representative amino acid sets. Dissimilar ϕ vs χ_1_ basins in protein structures for various amino acid types. Differential basin preferences for different amino-acids are calculated from the entire database of 22,977 non-redundant structures. Localization for the χ_1_ and ϕ dihedral rotors in protein structures are evident.

From the above analysis, we may conclude that there are two distinctly different rotor motions operative for helix and sheet formation. A perturbation to this rotor motion will result in a sequence segment of coil that connects between successive secondary structure elements. In this general scenario, it would be interesting to see whether amino-acids will respond differently to specific secondary structures, while rotor motions are executed by the chain. This is of a very significant importance, in light of the fundamental question of sequence–structure relationship in the protein folding problem. Main chain of any protein sequence is a constant, but the side-chain is believed to influence its evolution to a complete functional fold. So far, studies along these lines, were biased to focus on the chemistry, polarity, β-branching, hydrophobicity, aromaticity, size etc. Our significant lead from the first part of this investigation is that the rotation patterns of side-chain rotors directly influence main chain rotor motions, which will eventually decide the structure. Based on the standard parameters, Gln, Ile, Pro, Trp, Tyr and Val were selected as representative residues initially, to evaluate our objective to investigate side-chain geometric influences on main chain structure evolution. The dihedral angle distributions were calculated and plotted as described in the previous section for each of the amino acids and classified as helices and sheets. The residue-wise data thus generated, was similar not only to the cumulative dataset presented in the previous sections, but also brought out new insights into our perspective (Fig 4, Figure A5 in S1 File). The Linear side chains, both light (Gln) and bulky (Trp & Tyr) side-chains showed similar dihedral angle rotations. The χ_1_ for Pro, like ϕ wasn’t allowed much conformations as compared to others, but was present in two continuous basins viz. (-150)-(-180) and (130)-(180) (Figure A6-A25 in S1 File). The left handed helix conformation was also seen at flanking positions in most of the residues with more prominence in Gln and Lys, while completely missing in Ile, Val and Pro (A26-A39 in S1 File). This observation is in concordance with the observations by Kleywegt *et al*. [31].

In our earlier analysis, we found that χ_1_ and χ_2_ are counter-rotating while secondary structure formation, and direction of χ_1_ rotor is the most critical factor influencing ϕ rotor. Therefore, we mapped χ_1_ *vs* χ_2_ and ϕ *vs* χ_1_, basins during secondary structure formation and breaking (Fig 4, Figures A6-A17 in S1 File). Interestingly, all seven amino acids we choose are distinctly different in their basin distribution profiles (Figures A6-A44 in S1 File). This partially answers how structure of a sequence is unique, owing to the observation that the side-chain and main chain-rotor that directs their structural evolution and pathway, are distinctly different and unique.

### Translation of rotor preferences to substitution matrices: MIDMAT Series

The data generated had information about dihedral angle distribution for amino acids in protein structure. We decided to extend the study to all amino acids and test it on a sequence alignment platform. Hence, a methodology was developed to convert this distribution data into three possible substitution matrices. The majority of the scoring matrices are based on the evolution of the protein sequence, whereas our scoring matrices are derived from the experimental structural data. To construct the matrices, the geometrical arrangements of dihedral angles were distributed into four basins from +150 to -150 (via ±180), -150 to -50, -50 to +50 and +50 to +150 degrees in the rotational plane. The frequency of appearance of each amino acid was calculated for each basin and the basin energy for each reference basin was calculated as per the following equation:

$$∆G=-RTln\frac{N_{x}}{B}$$ (1)

Where, ∆*G* is the energy of the basin, R is the Universal Gas Constant, T is temperature (kept constant at 25°C), *N*_*x*_ is the number of basin entries, and *B* is the variable which was computed in three different ways for three different matrices proposed.

For each of the matrices, B represents the following; i) the sum of hits in remaining basins, ii) average of remaining basins and iii) average over all the four basins. Thus, to generate these three values of B for the first basin (Basin1), we used the following set of equations for each matrix

$$\mathrm{MIDMAT1:}B_{i}=\left(\sum_{i=1}^{n}b_{i}\right)-b_{i}$$ (2)

$$\mathrm{MIDMAT2:}B_{i}=\frac{\left(\left(\sum_{i=1}^{n}b_{i}\right)-b_{i}\right)}{n-1}$$ (3)

$$\mathrm{MIDMAT3:}B_{i}=\frac{\left(\sum_{i=1}^{n}b_{i}\right)}{n}$$ (4)

where, B_i_ is the value of B for *i*^th^ basin and b_i_ is the sum of total number of entries in *i*^th^ basin

The basin energies for each amino acid were thus calculated for the four rotors (ϕ, ψ, χ_1_ and χ_2_). Comparative estimates of corresponding basins for any given amino acid pair was measured based on the Euclidean distance calculation as per the following equation.

$$D_{xy}=\sum_{i=1}^{n}\sqrt{{\left(\Phi_{ix}-\Phi_{iy}\right)}^{2}+{\left(\Psi_{ix}-\Psi_{iy}\right)}^{2}+{\left(\chi_{1ix}-\chi_{1iy}\right)}^{2}+{\left(\chi_{2ix}-\chi_{2iy}\right)}^{2}}$$ (5)

where, D_xy_ is the distance (indicates the dissimilarity between the rotor preferences of two amino acids) from amino acid x to amino acid y, and ϕ_i_, ψ_i_, χ_1i_, χ_2i_ represent the basin energies for the *i*^th^ basin for ϕ, ψ, χ_1_ and χ_2_ dihedral distributions for the amino acids x and y.

The distances value calculated for all amino acid pairs has the very nature and physical meaning of a typical substitution matrix. The values of this distance matrix were then normalized by dividing all the values by a common denominator, 1000.

The values thus generated in the distance matrix were further processed to calculate substitution values between each pair of two amino acids x and y (SV_xy_) by subtracting each of them with the median value of the distance matrix and multiplying with a normalization factor of 0.5 as illustrated below.

$${SV}_{xy}=0.5\times \left(Median\left(\left(D_{x1y1}\right)\left(D_{x1y2}\right)..\left(D_{\mathrm{xn yn}}\right)\right)-D_{xy}\right)$$ (6)

To calculate the substitution value of an amino acid with itself (e.g. ala with ala), we took the maximum substitution value (SV) from the substitution matrix and added it to the existing maximum substitution value of the same amino acid.

$${SV}_{xx}=Max(\begin{array}{ccc} {SV}_{x1y1} & {SV}_{x1y2}\ldots & {SV}_{xn\mathrm{yn}} \end{array})+Max(\begin{array}{ccc} {SV}_{x1y1} & {SV}_{x1y2}\ldots & {SV}_{x1\mathrm{yn}} \end{array})$$ (7)

The values were then rounded off to the nearest decimal point to give the final substitution score required for the construction of three substitution matrices, MIDMAT1 MIDMAT2 and MIDMAT3 (Tables 1, 2 and 3).

**Table 1:** MIDMAT 1 substitution matrix. MIDMAT 1 substitution matrix values are calculated based on basin statistics derived from the rotor combinations of amino acids in the structural dataset of 22,997 non-redundant structures from PISCES server. The amino acids are represented as their single letter codes.

	A	R	N	D	C	Q	E	G	H	I	L	K	M	F	P	S	T	W	Y	V
A	9	-2	-1	0	1	-2	-2	-1	-1	-3	-4	-3	-2	-1	-5	2	1	-1	-1	0
R	-2	15	-1	-1	-1	6	6	-6	2	3	2	7	6	0	-9	-1	-1	0	0	-2
N	-1	-1	13	6	3	0	-1	-2	3	-3	-2	-1	0	3	-9	2	1	3	3	-1
D	0	-1	6	13	3	0	-1	-3	3	-3	-2	-1	0	3	-8	3	2	4	2	0
C	1	-1	3	3	13	0	-1	-2	3	-1	-2	-1	0	2	-7	5	5	3	2	3
Q	-2	6	0	0	0	14	7	-5	3	3	2	7	6	1	-8	0	-1	1	1	-1
E	-2	6	-1	-1	-1	7	14	-6	2	3	2	6	6	1	-8	-1	-1	0	1	-2
G	-1	-6	-2	-3	-2	-5	-6	6	-4	-8	-8	-6	-6	-5	-10	-2	-4	-5	-5	-6
H	-1	2	3	3	3	3	2	-4	12	0	-1	2	3	5	-9	2	1	4	5	0
I	-3	3	-3	-3	-1	3	3	-8	0	11	1	3	4	0	-9	-2	-1	-1	0	-2
L	-4	2	-2	-2	-2	2	2	-8	-1	2	10	2	2	-1	-9	-3	-3	-2	-2	-2
K	-3	7	-1	-1	-1	7	6	-6	2	3	3	14	6	0	-9	-1	-2	0	0	-2
M	-2	6	0	0	0	6	6	-6	3	4	3	6	14	2	-8	0	0	1	2	-1
F	-1	0	3	3	3	1	0	-5	5	0	-1	0	2	15	-8	1	1	6	7	1
P	-4	-9	-9	-8	-7	-8	-8	-10	-9	-9	-9	-9	-8	-8	3	-7	-7	-7	-8	-6
S	2	-1	2	3	5	0	-1	-2	2	-2	-3	-1	0	2	-7	13	5	2	1	2
T	1	-1	1	2	5	-1	-1	-4	1	-1	-2	-2	0	1	-7	5	12	2	1	2
W	0	0	3	4	3	1	0	-5	4	-1	-2	0	1	6	-7	2	2	13	6	1
Y	-1	0	3	2	2	1	0	-5	5	0	-2	0	2	7	-8	1	1	6	15	1
V	0	-2	-1	0	3	-1	-1	-6	0	-2	-2	-2	-1	2	-6	2	2	2	1	10

**Table 2:** MIDMAT 2 substitution matrix. MIDMAT 2 substitution matrix constructed using a different approach than MIDMAT1 as discussed in results section, from the same set of 22,997 non-redundant structures from PISCES server. The amino acids are represented as single letter codes.

	A	R	N	D	C	Q	E	G	H	I	L	K	M	F	P	S	T	W	Y	V
A	10	-3	-1	-1	1	-2	-2	-2	-1	-4	-5	-3	-2	-2	-5	2	1	-1	-2	0
R	-1	14	-1	-1	-1	6	6	-6	2	2	2	7	5	0	-9	-1	-2	-1	0	-2
N	1	-1	13	5	3	0	-1	-3	3	-3	-2	-1	0	3	-9	2	1	3	2	-1
D	1	-1	5	12	3	-1	-1	-3	2	-3	-2	-1	-1	2	-8	2	1	3	2	0
C	2	0	4	4	12	0	-1	-3	2	-2	-2	-1	0	2	-8	5	4	2	2	2
Q	0	6	0	-1	1	13	6	-6	3	2	2	6	6	1	-9	-1	-1	0	1	-2
E	0	6	-1	-1	1	6	13	-6	2	3	2	6	6	0	-8	-1	-2	0	0	-2
G	-2	-4	-1	-2	-2	-4	-5	6	-4	-9	-9	-6	-6	-6	-11	-2	-4	-5	-6	-6
H	1	2	3	2	4	3	2	-3	12	0	-1	2	3	5	-9	1	1	4	5	0
I	-2	2	-3	-3	-1	2	3	-8	0	10	1	2	3	-1	-9	-3	-1	-1	-1	-2
L	-2	2	-2	-2	-1	2	2	-7	-1	1	9	2	2	-2	-9	-3	-3	-2	-2	-2
K	-1	7	-1	-1	1	6	6	-4	2	2	2	14	6	0	-9	-1	-2	-1	0	-2
M	0	5	0	-1	1	6	6	-5	3	3	2	6	13	1	-9	-1	-1	1	1	-1
F	0	0	3	2	4	1	0	-4	5	-1	-2	0	1	14	-9	1	1	6	7	1
P	-4	-9	-9	-8	-8	-9	-8	-10	-9	-9	-9	-9	-9	-8	2	-7	-7	-7	-9	-6
S	3	0	3	3	5	1	0	-1	3	-1	-2	0	1	2	-7	12	4	2	1	1
T	2	-1	2	2	4	0	0	-3	2	0	-2	-1	0	2	-7	4	12	2	1	1
W	1	-1	3	3	4	0	0	-4	4	-1	-2	-1	1	6	-7	3	3	13	6	1
Y	0	0	2	2	3	1	0	-4	5	-1	-2	0	1	7	-8	2	2	6	14	1
V	1	-1	0	0	2	0	-1	-6	1	-1	-1	-1	0	2	-6	1	1	2	2	9

**Table 3:** MIDMAT 3 substitution matrix. MIDMAT3 substitution matrix constructed by following the third strategy (results section) for calculation of B_i_, from the identical data set of 22,997 non-redundant structures from PISCES server.

	A	R	N	D	C	Q	E	G	H	I	L	K	M	F	P	S	T	W	Y	V
A	9	-1	1	1	2	0	0	-2	1	-1	-1	-1	0	1	-3	3	2	1	1	1
R	-1	12	-1	-1	1	5	5	-4	2	2	1	6	5	0	-7	0	0	0	1	-1
N	1	-1	11	5	3	0	-1	-1	3	-3	-1	0	0	2	-7	3	1	2	2	0
D	1	-1	5	11	4	0	-1	-2	3	-3	-1	-1	0	3	-6	3	2	3	2	0
C	2	1	3	4	11	1	1	-2	4	-1	0	1	1	3	-5	5	4	4	3	2
Q	0	5	0	0	1	12	6	-4	3	2	2	6	5	1	-6	1	0	0	1	0
E	0	5	-1	-1	1	6	12	-4	2	2	1	5	5	1	-6	0	0	0	1	0
G	-2	-4	-1	-2	-2	-4	-4	5	-3	-7	-6	-4	-4	-4	-8	-1	-3	-4	-4	-5
H	1	2	3	3	4	3	2	-3	10	0	0	2	3	4	-6	3	2	3	4	1
I	-1	2	-3	-3	-1	2	2	-7	0	9	1	2	3	0	-6	-1	0	-1	0	0
L	-1	1	-1	-1	0	2	1	-6	0	1	8	2	2	0	-6	-1	-1	0	0	0
K	-1	6	0	-1	1	6	5	-4	2	2	2	12	5	1	-7	0	0	0	1	0
M	0	5	0	0	1	5	5	-4	3	3	2	5	12	2	-6	1	1	1	2	1
F	1	0	2	3	3	1	1	-4	4	0	0	1	2	12	-6	2	2	5	6	2
P	-3	-7	-7	-6	-5	-6	-6	-8	-6	-6	-6	-7	-6	-6	3	-5	-4	-4	-6	-3
S	3	0	3	3	5	1	0	-1	3	-1	-1	0	1	2	-5	11	4	3	2	1
T	2	0	1	2	4	0	0	-3	2	0	-1	0	1	2	-4	4	10	3	2	2
W	1	0	2	3	4	0	0	-4	3	-1	0	0	1	5	-4	3	3	11	5	2
Y	1	1	2	2	3	1	1	-4	4	0	0	1	2	6	-6	2	2	5	12	2
V	1	-1	0	0	2	0	0	-5	1	0	0	0	1	2	-3	1	2	2	2	9

### Evaluation of MIDMAT1, MIDMAT2 and MIDMAT3

**Fig 5:**
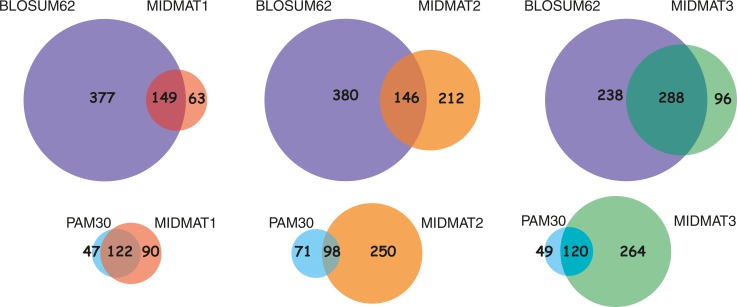
Similar and unique hits for MIDMATs compared to BLOSUM62 and PAM30: The number of total, common and unique hits scored by MIDMATs (MIDMAT 1, MIDMAT 2 and MIDMAT 3), against BLOSUM62 and PAM30 matrices when plugged into a BLAST program against PDB, using the CASP11 database as a query set, while maintaining the default parameters of BLAST.

The constructed matrices, MIDMAT1, MIDMAT2 and MIDMAT3 were tested for their efficacy as substitution scoring matrices by employing them in the BLAST program. CASP11 data set was used as a test set, because majority of proteins in CASP11 have minimal sequence similarity with the structures available in PDB at the time of submission. All three matrices were successful in aligning the sequences from CASP11 dataset with their respective PDB IDs, now available in PDB. The results were promising for the MIDMATs and MIDMAT3 consistently produced a better score and E-value for the best matching alignment than MIDMAT1 and MIDMAT2. This, however, is a simple qualitative demonstration highlighting the utility of three matrices generated solely on the basis of dihedral angle geometries of representative protein dataset. We have employed BLOSUM62 (25) and PAM30 (24) as standards. For a set of 67 proteins used for CASP11, the number of common and unique hits is represented by the Venn diagrams for MIDMATs, BLOSUM62 and PAM30 (Fig 4). As is evident from Fig 5, MIDMAT1 showed most similar results to PAM30, a matrix used for aligning highly similar sequences of a significantly small length, usually less than 50. MIDMAT3 is the most promising among all MIDMAT matrices in terms of scores and E-values. This, however, is a simple suggestive demonstration highlighting the utility of three matrices generated solely on the basis of dihedral angle geometries of representative protein dataset.

## Discussion

Folding of a protein molecule may be envisaged as an evolution of a given polypeptide chain from random to fixed geometry; with geometrical fixation of the final fold is decided by its amino acid sequence. Polypeptide chain has a characteristic ratio approximately 9 times more than a random flight chain indicating the constraint in the chain due to peptide bond, poly L stereochemistry and steric interaction. Practically, hugely restricted combinations of ϕ and ψ dihedral angles of main chain and χ_1_, χ_2_ etc. dihedral angles of side chain are the only variables in this complex process. Since main chain is a constant for all the chains, we made an assumption that the side chain geometrical variables χ_1_ and χ_2_ are guiding main chain geometrical variables ϕ and ψ. The results obtained were rather surprising with secondary structure elements showing distinctly different geometrical rotation patterns while formation and breaking. We modelled the protein molecule as an ‘enigma machine’ with the directive of its geometrical evolution encoded in its sequence. The four geometrical variables are modelled as four rotors of this molecular machine. Amino acid sequence is essentially its side chain sequence and therefore, we could logically assume that side chain rotors χ_1_ and χ_2_ are directing main chain rotors. We could show that their differential motions differentiate secondary structure formations. We further examined seven amino acids of diverse side chain type to evaluate their rotor motion. The observations that all seven were distinctly different in their side chain and main chain rotor combinations further strengthened our belief that side chain geometry controls main chain geometries of a poly-peptide chain. We further extended the study to all amino acids by calculating free energy of each dihedral angle combination basins from their population statistics. All amino acids are compared against each other and quantified in the form of 3 substitution matrices. Their performances are compared by plugging in a BLAST engine. The results of these comparative analysis with standard substitution matrices like BLOSUM62 and PAM30 further underlies the efficacy of these substitution matrices in sequence alignment tools. However, a detailed study on their relative efficacy when compared to other scoring matrices (BLOSUM62 and PAM) is beyond the scope of this paper, hence not attempted.

We believe that our study is throwing light on the following fundamental aspects of protein folding. i) How an amino acid sequence choose a secondary structure. ii) How side chain geometries and its combination with main chain geometries directs the evolution of a random chain to its prescribed fold and iii) How different sequences generally end up in different fold. The emphasis on ‘How’ rather than ‘why’ in our investigation may perhaps helped us in proposing some important observations and a model, though they are of suggestive nature than that of a conclusive one.

## Supporting Information

S1 File Supplementary information. (PDF)
