# Supplementary material for: Mapping the Geometric Evolution of Protein Folding Motor
Source: PLoS One. 2016 Oct 7;11(10):e0163993. doi: 10.1371/journal.pone.0163993 (PMC5055333; doi:10.1371/journal.pone.0163993)
Supplement: S1 File — (PDF) [file pone.0163993.s001.pdf]

SUPPORTING INFORMATION

Mapping the Geometric Evolution of Protein Folding Motor

*Gaurav Jerath [1], Prakash Kishore Hazam [1], Shashi Shekhar [1] and Vibin Ramakrishnan [1]**

[1] Department of Biosciences and Bioengineering, Indian Institute of Technology Guwahati, Assam, India

* To whom correspondence should be addressed. E-mail: vibin@iitg.ernet.in

Comparative dihedral Rotor motion for secondary structures

Differential motion of rotors result in different secondary structure elements. The relative movement of dihedral rotors during formation and breaking of helix and sheet have been shown in the Supplemental Fig A1 (ψ and ϕ), A2 (χ_1 and χ_2), A3 (ϕ and χ_2) and A4 (ψ and χ_2). The remaining combinations have been represented in the manuscript as Fig 2.

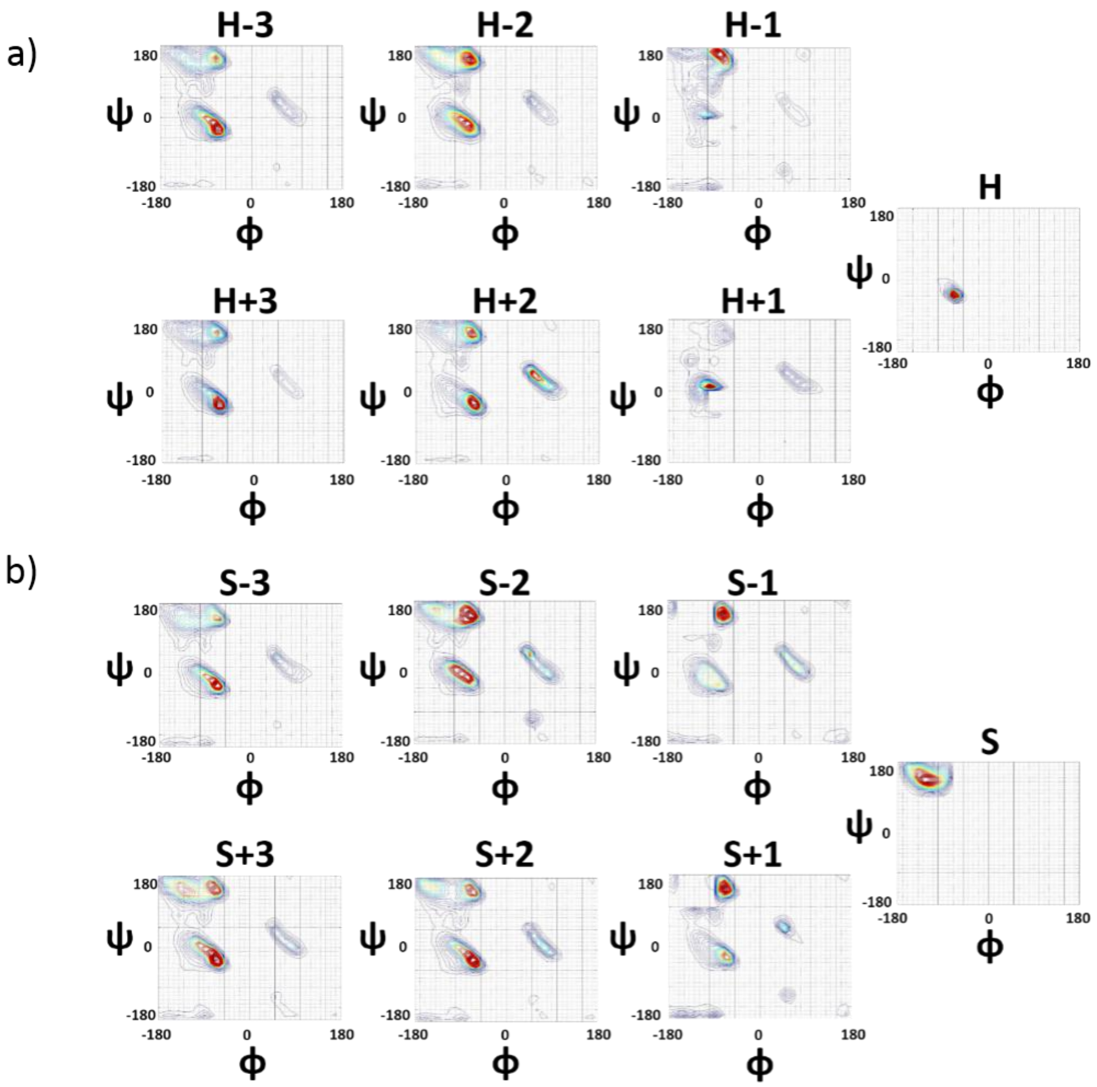

Fig A1. Related to Fig 2: Making and breaking of secondary structures. ψ vs ϕ for helix (a) and sheets (b). The plots depict the relative shifts in basin for ψ and ϕ dihedral rotors during secondary structure formation and breaking.

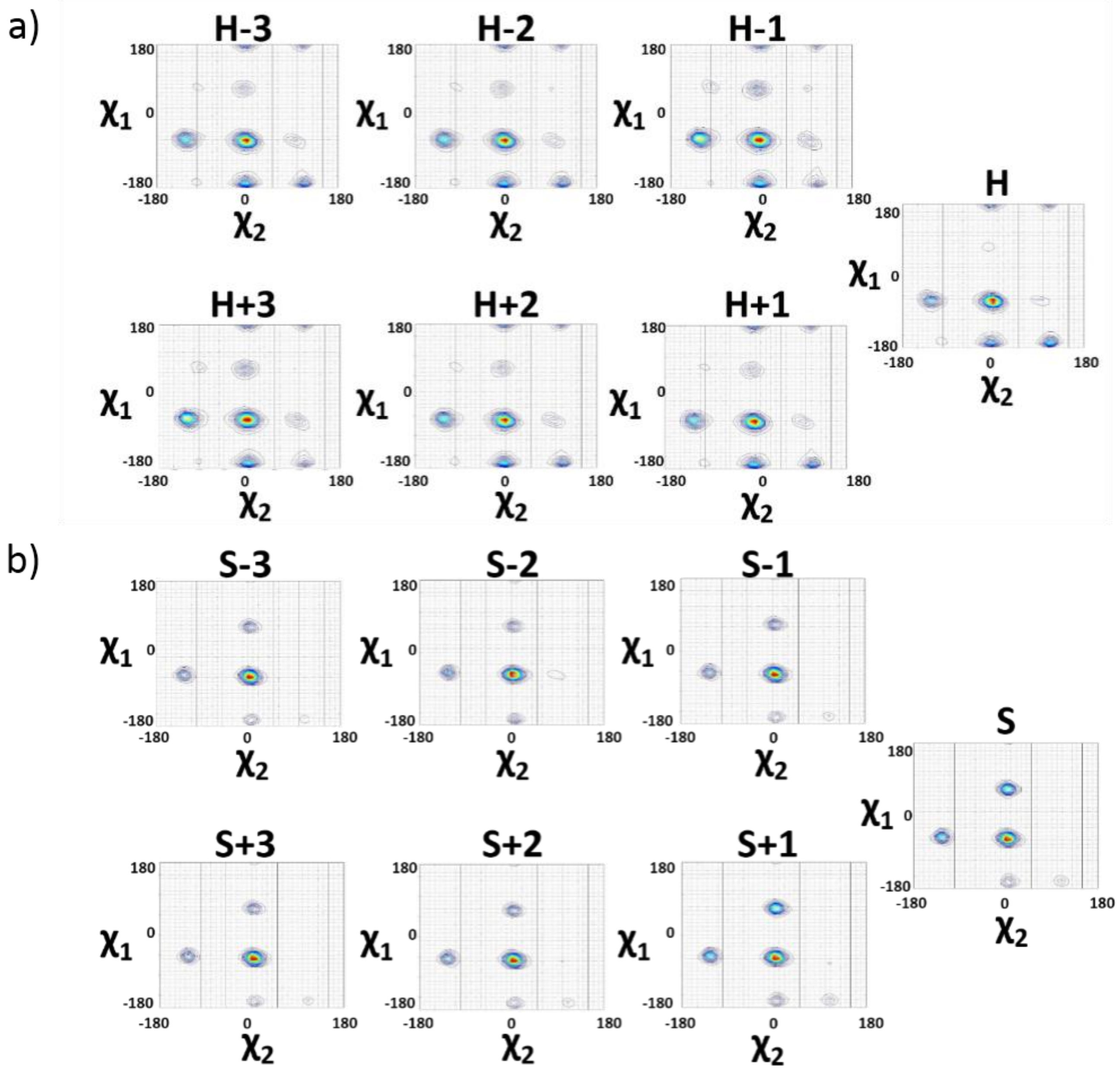

Fig A2. Related to Fig 2: **Making and breaking of secondary structures.** χ_1 vs χ_2 for helix (a) and sheets (b). The plots depict the relative shifts in basin for χ_1 and χ_2 dihedral rotors during secondary structure formation and breaking.

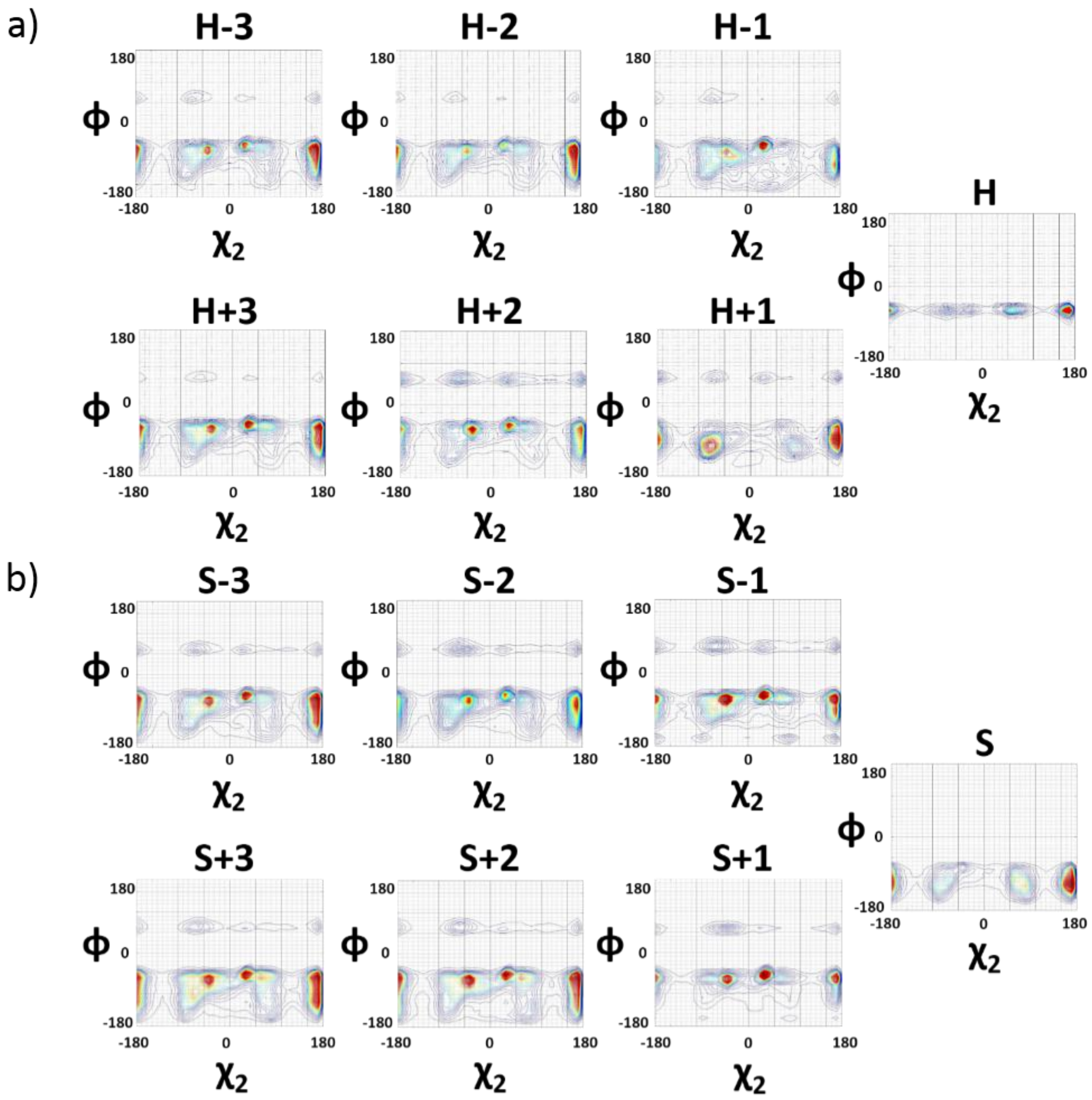

Fig A3. Related to Fig 2: Making and breaking of secondary structures. ϕ vs χ_2 for helix (a) and sheets (b). The plots depict the relative shifts in basin for ϕ and χ_2 dihedral rotors during secondary structure formation and breaking.

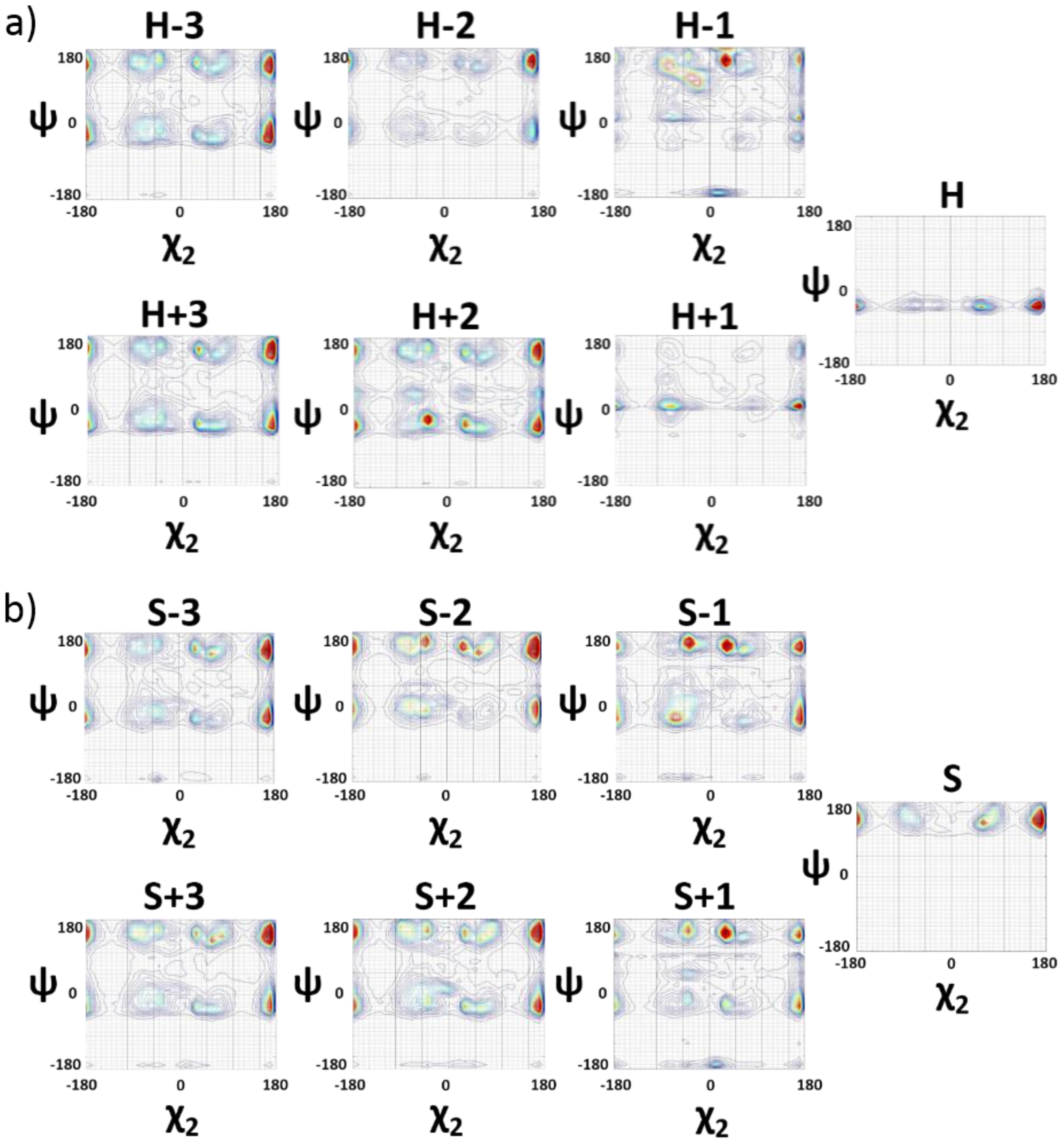

Fig A4. Related to Fig 2: **Making and breaking of secondary structures.** ψ vs χ_2 for helix (a) and sheets (b). The plots depict the relative shifts in basin for ψ and χ_2 dihedral rotors during secondary structure formation and breaking.

Differential amino acid wise preference ϕ and χ_1 Rotor orientations

The ϕ and χ_1 rotor preferences for different amino acids, not included in the manuscript have been illustrated in Fig A5.

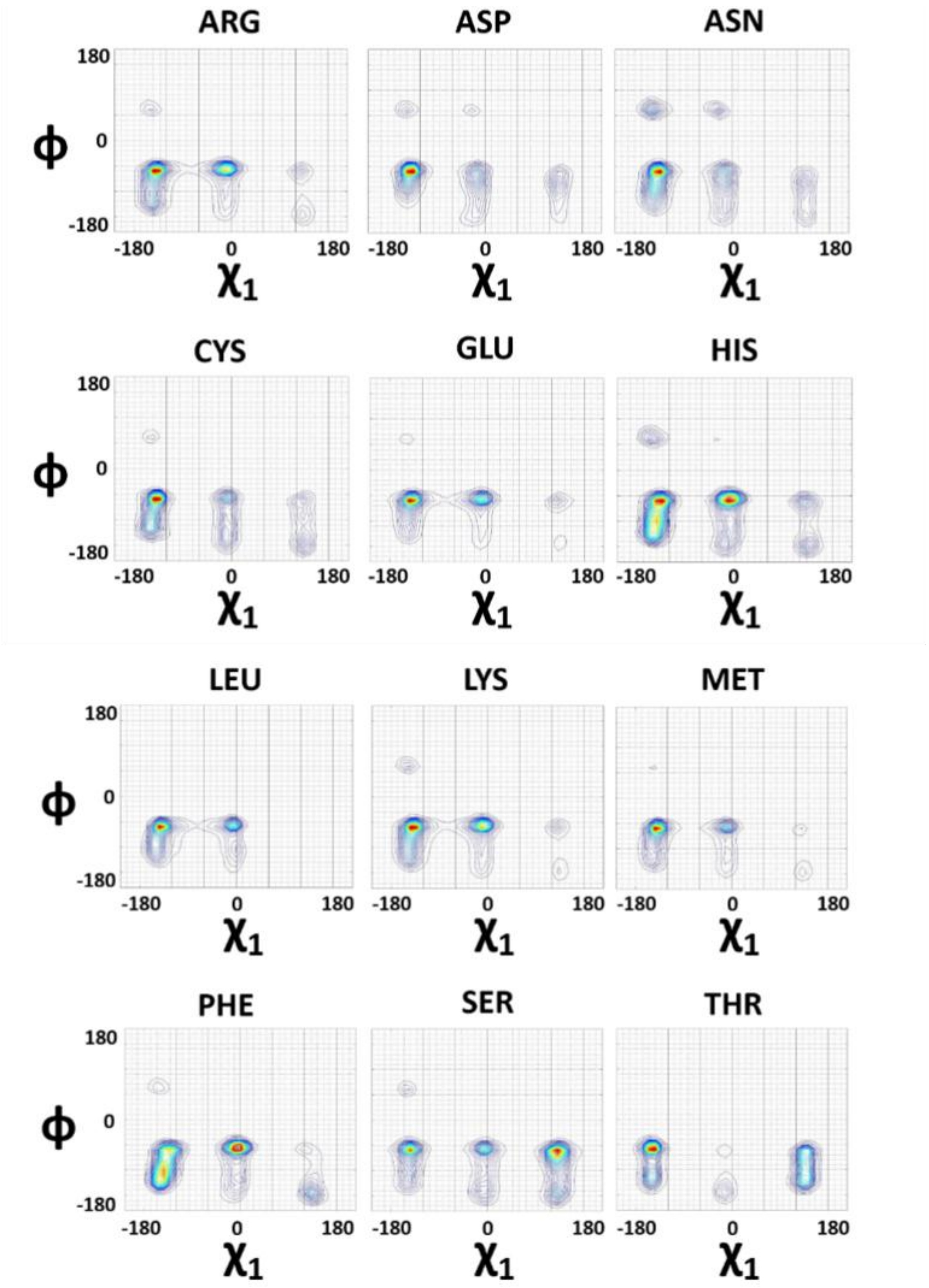

Fig A5. Related to Fig 3: **Dissimilar ϕ vs χ_1 basins for various amino acids in Protein structures.** The different basins of localization for the χ_1 and ϕ dihedral rotors in protein structures are evident. The amino acid names are represented above the individual basin plots.

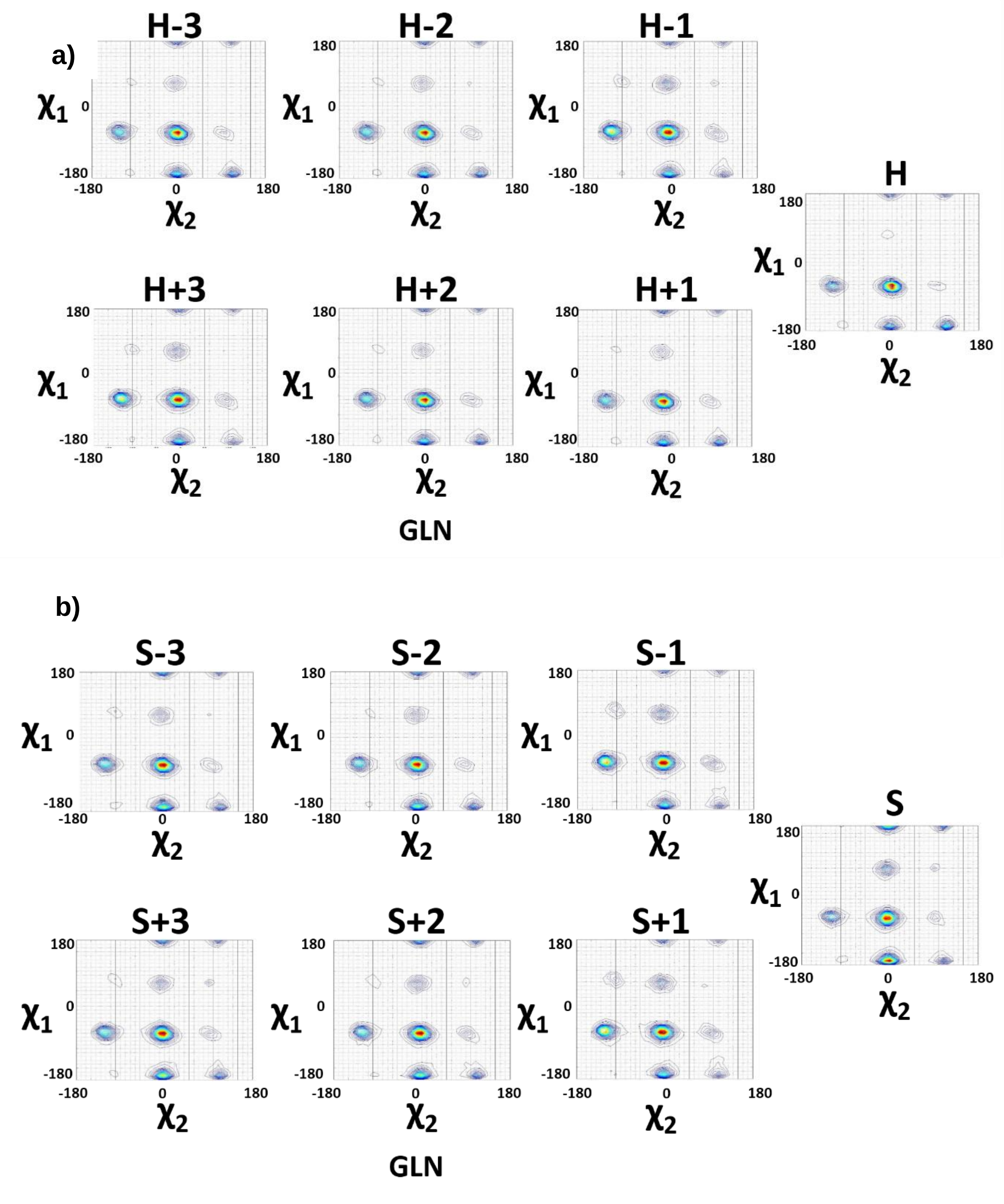

Fig A6: **Propensity of GLN in secondary structures.** χ_1 vs χ_2 for helix (a) and sheets (b). The plots depict the most prominent in basins of localization for χ_1 and χ_2 dihedral rotors in GLN during secondary structure formation and breaking.

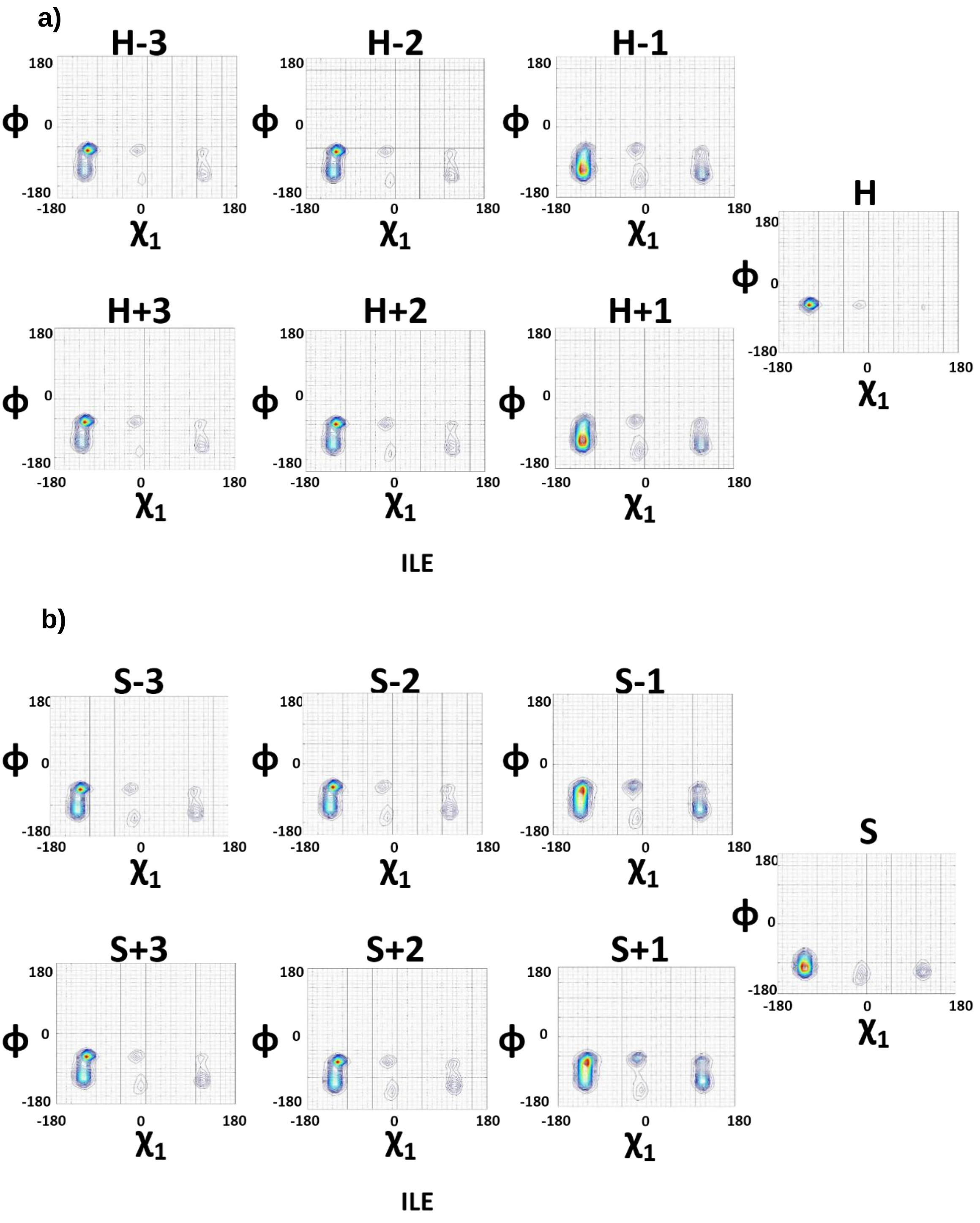

Fig A7: **Propensity of ILE in secondary structures.** χ_1 vs χ_2 for helix (a) and sheets (b). The plots depict the most prominent in basins of localization for χ_1 and χ_2 dihedral rotors in ILE during secondary structure formation and breaking.

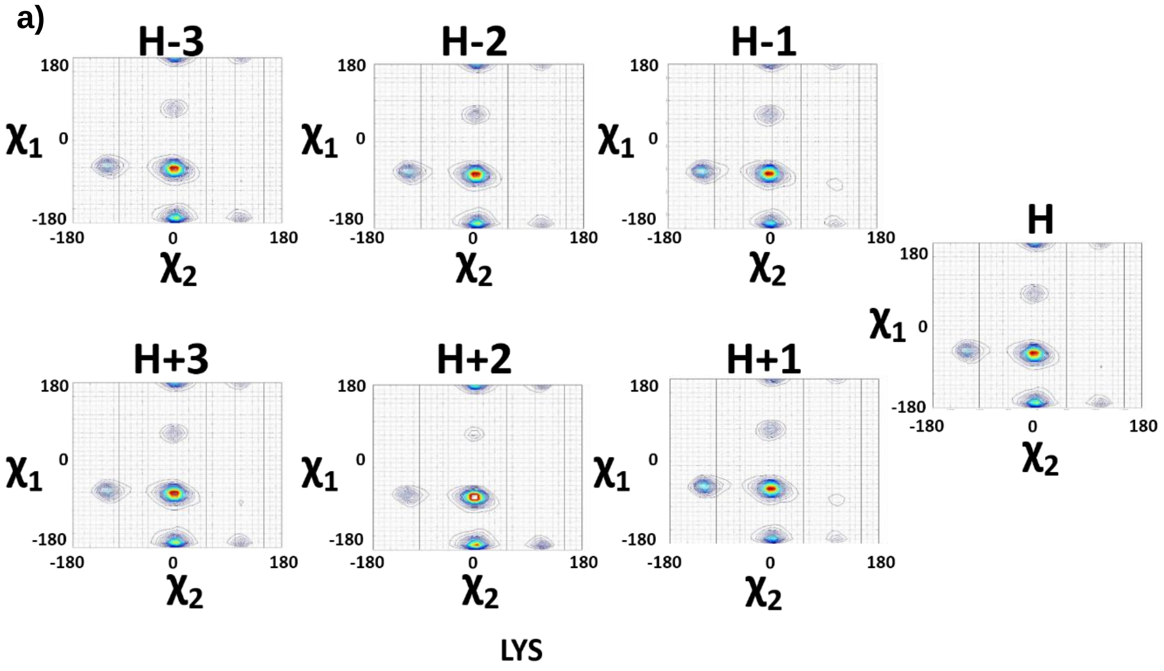

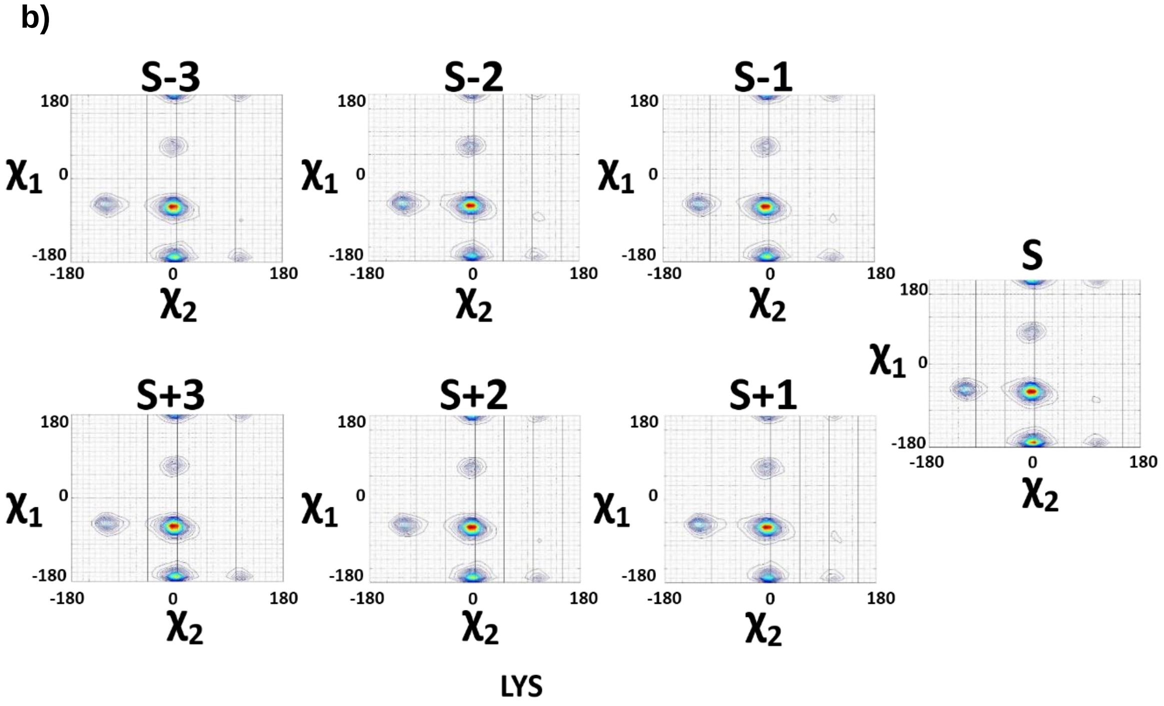

Fig A8: **Propensity of LYS in secondary structures.** χ_1 vs χ_2 for helix (a) and sheets (b). The plots depict the most prominent in basins of localization for χ_1 and χ_2 dihedral rotors in LYS during secondary structure formation and breaking.

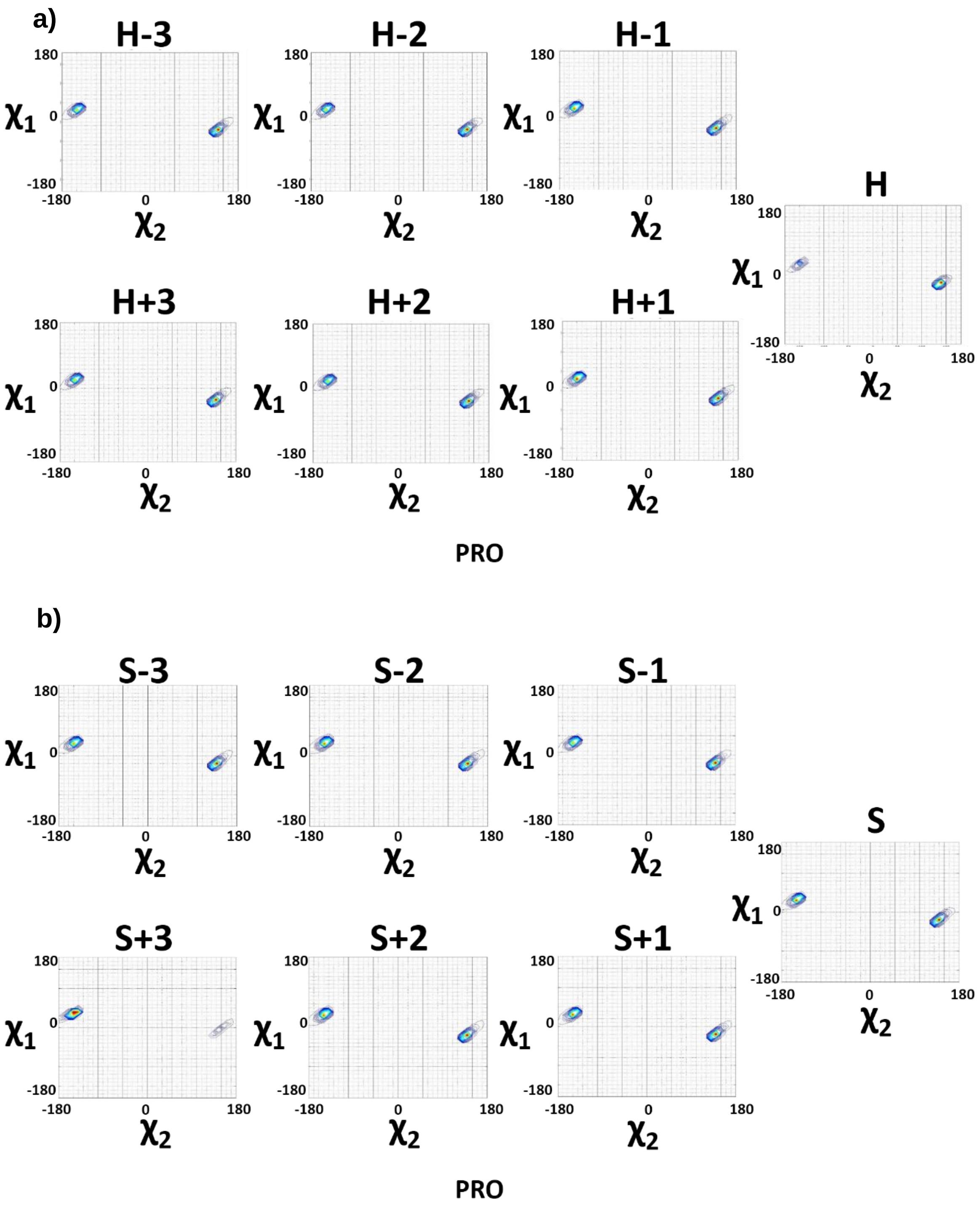

Fig A9: **Propensity of PRO in secondary structures.** χ_1 vs χ_2 for helix (a) and sheets (b). The plots depict the most prominent in basins of localization for χ_1 and χ_2 dihedral rotors in PRO during secondary structure formation and breaking.

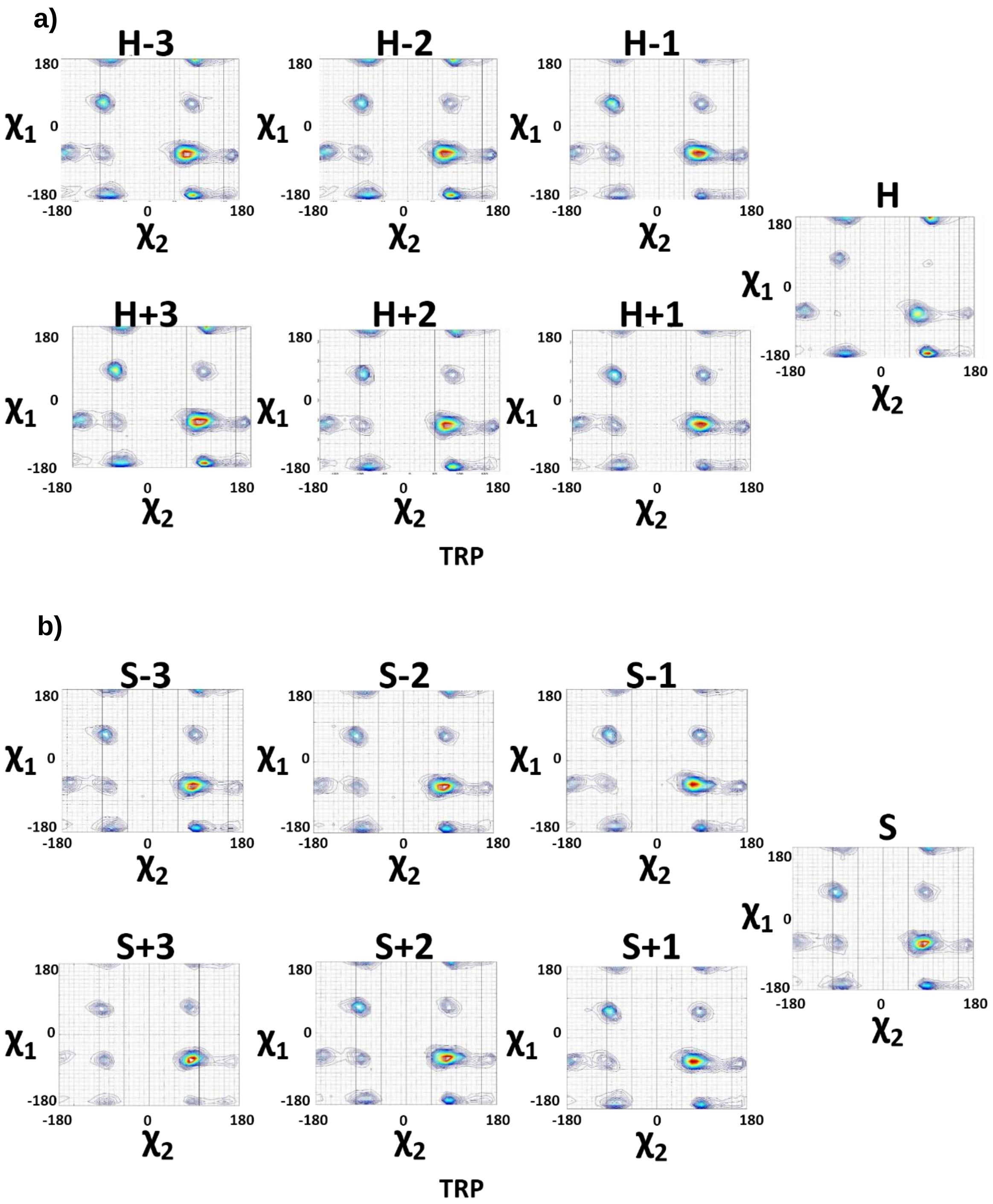

Fig A10: **Propensity of TRP in secondary structures.** χ_1 vs χ_2 for helix (a) and sheets (b). The plots depict the most prominent in basins of localization for χ_1 and χ_2 dihedral rotors in TRP during secondary structure formation and breaking.

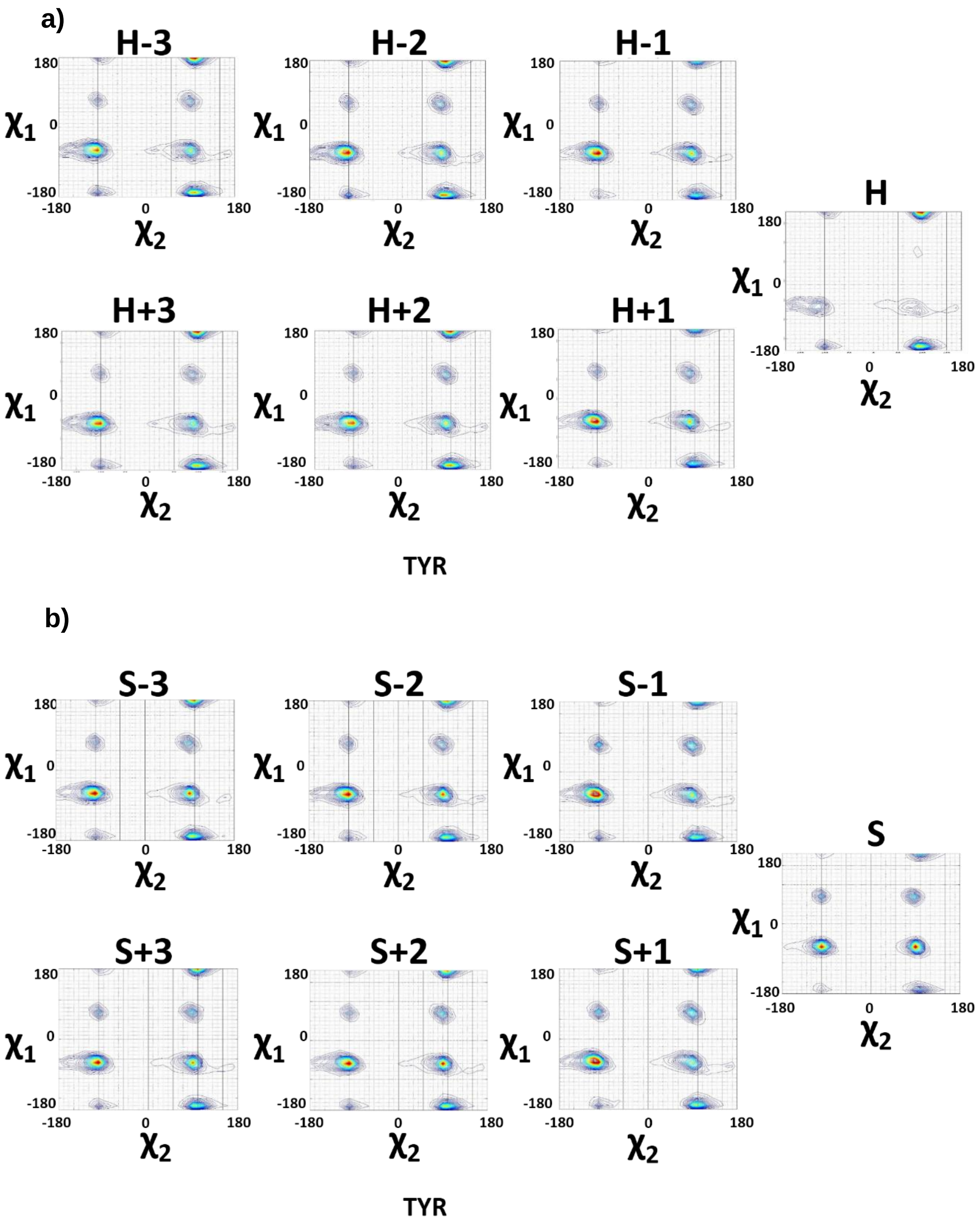

Fig A11: **Propensity of TYR in secondary structures.** χ_1 vs χ_2 for helix (a) and sheets (b). The plots depict the most prominent in basins of localization for χ_1 and χ_2 dihedral rotors in TYR during secondary structure formation and breaking.

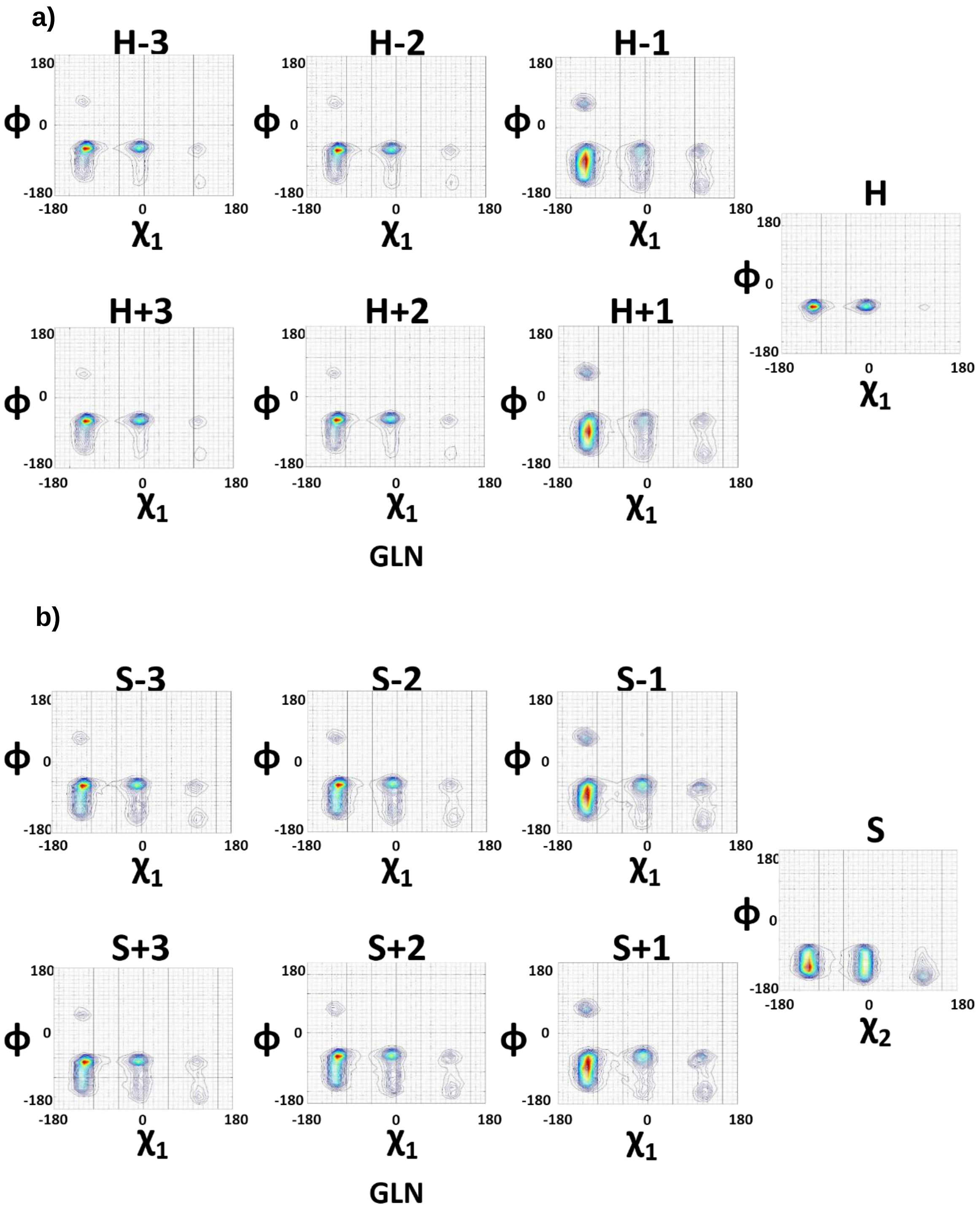

Fig A12: **Propensity of GLN in secondary structures.** ϕ vs χ_1 for helix (a) and sheets (b). The plots depict the most prominent in basins of localization for ϕ and χ_1 dihedral rotors in GLN during secondary structure formation and breaking.

a)

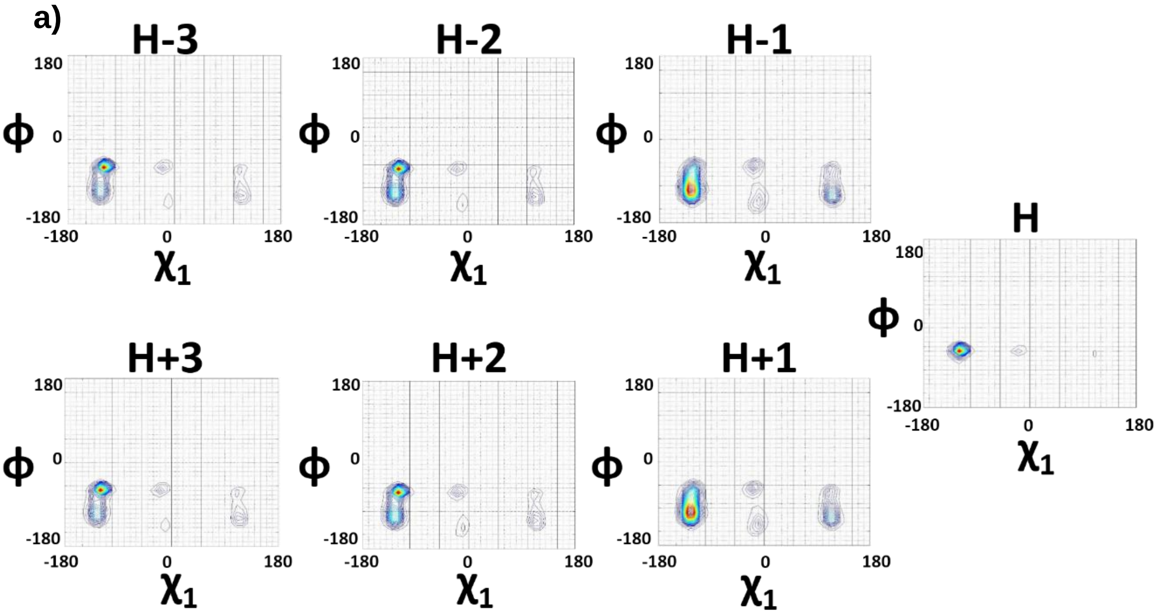

ILE

b)

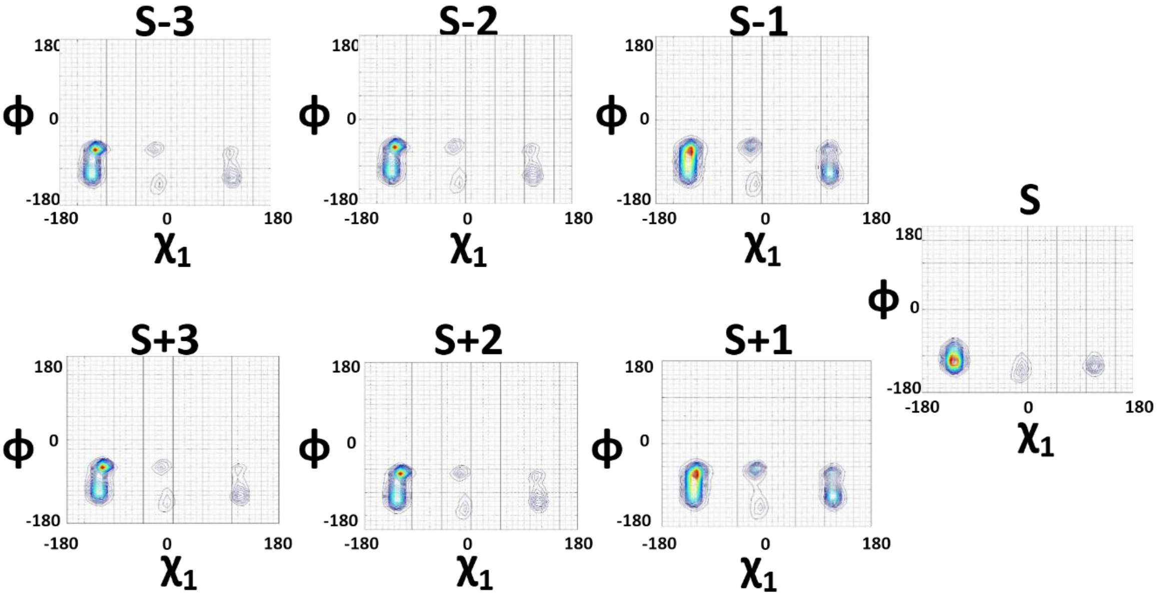

ILE

Fig A13: **Propensity of ILE in secondary structures.** ϕ vs χ_1 for helix (a) and sheets (b). The plots depict the most prominent in basins of localization for ϕ and χ_1 dihedral rotors in ILE during secondary structure formation and breaking.

a)

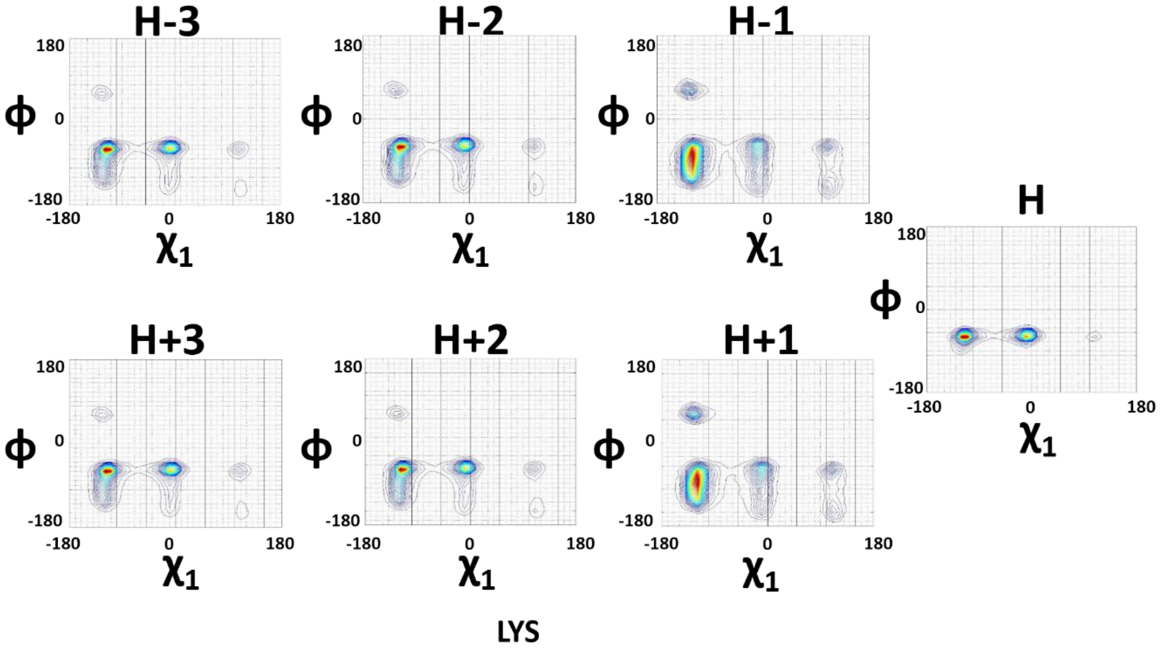

b)

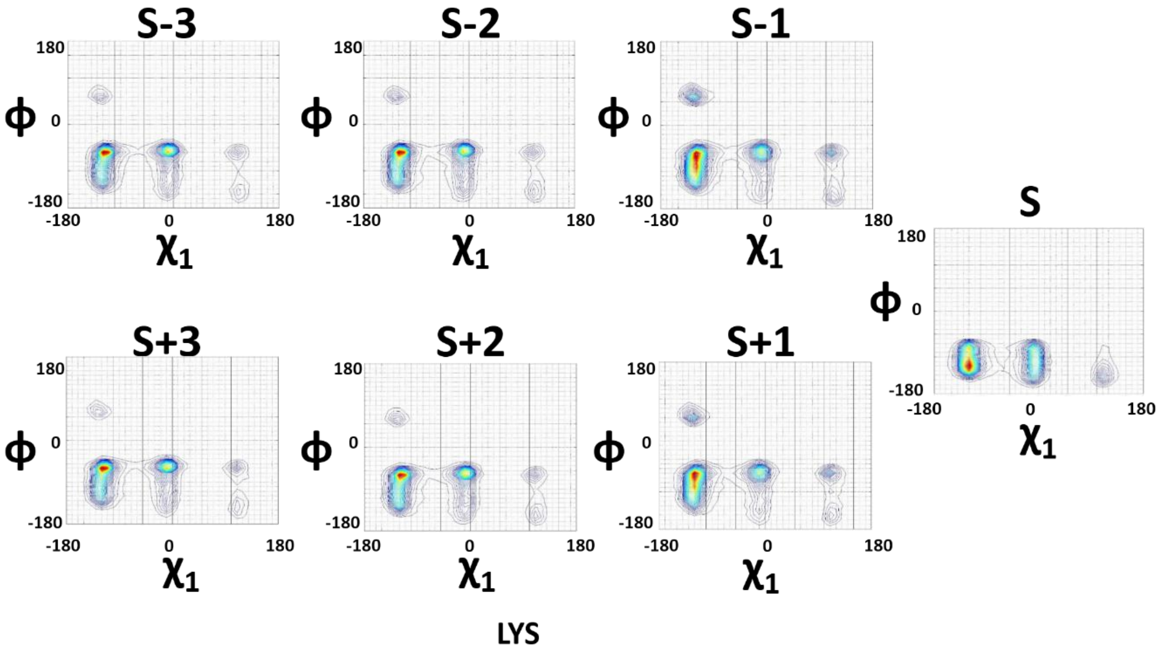

Fig A14: **Propensity of LYS in secondary structures.** ϕ vs χ_1 for helix (a) and sheets (b). The plots depict the most prominent in basins of localization for ϕ and χ_1 dihedral rotors in LYS during secondary structure formation and breaking.

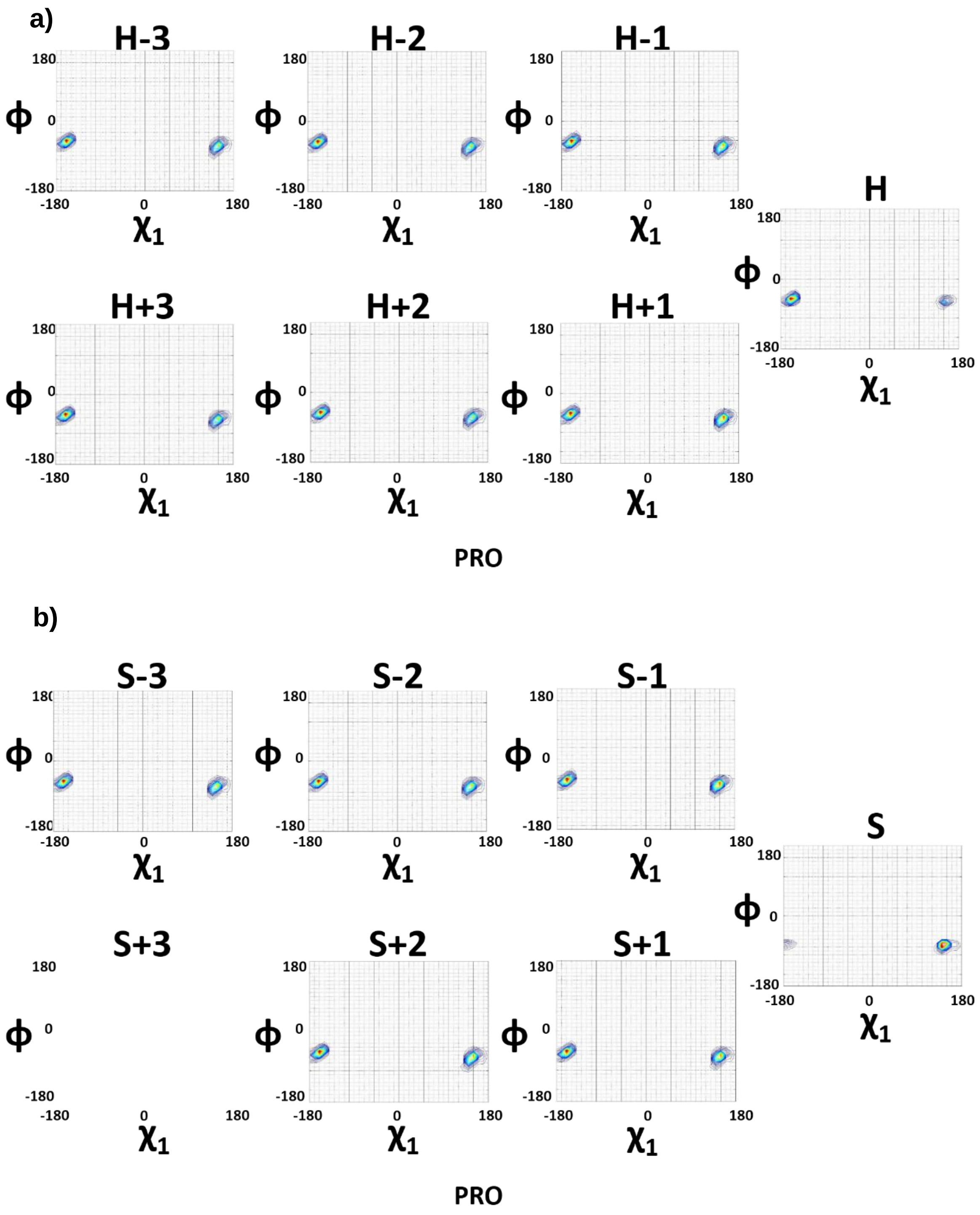

Fig A15: **Propensity of PRO in secondary structures.** ϕ vs χ_1 for helix (a) and sheets (b). The plots depict the most prominent in basins of localization for ϕ and χ_1 dihedral rotors in PRO during secondary structure formation and breaking.

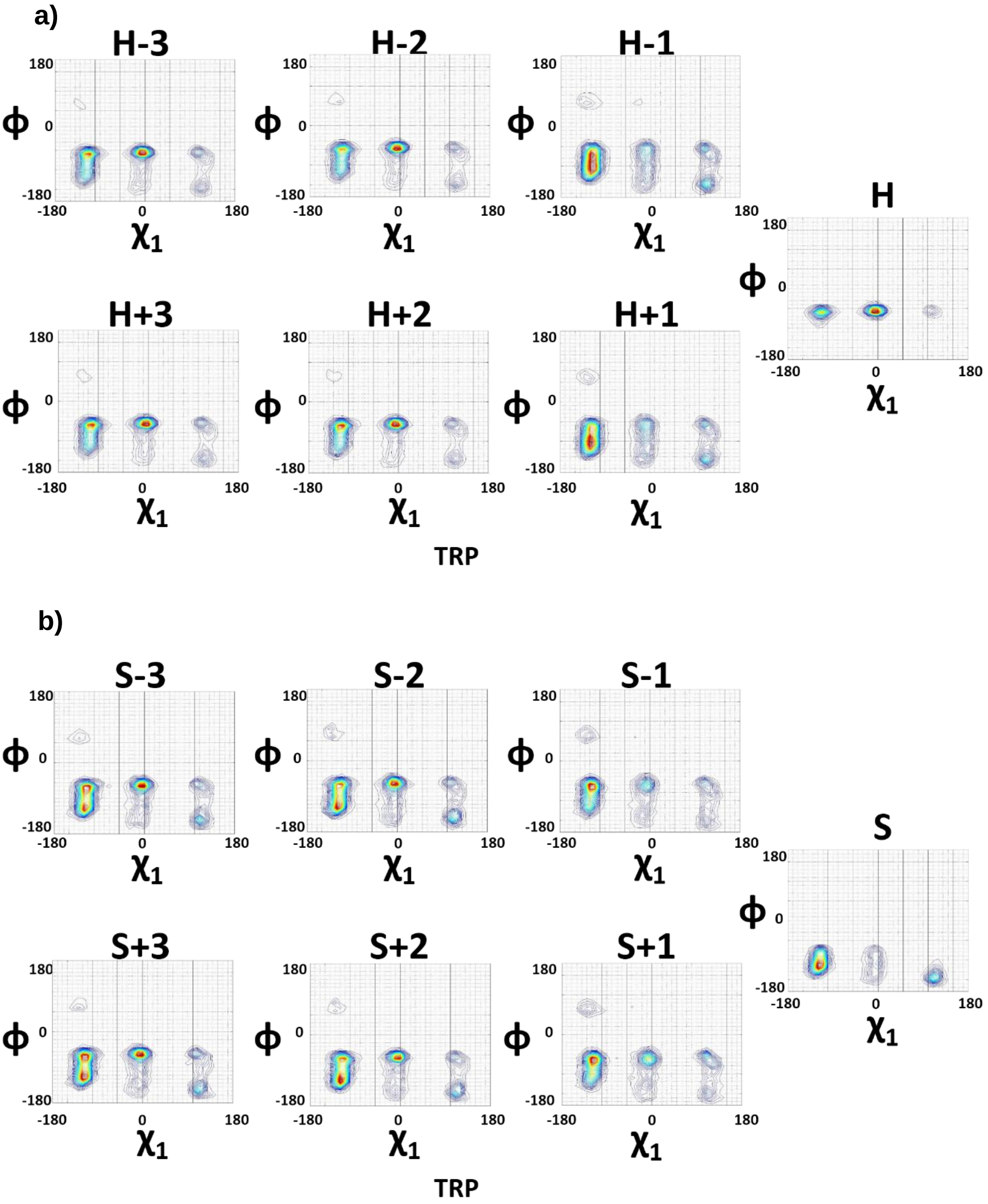

Fig A16: **Propensity of TRP in secondary structures.** ϕ vs χ_1 for helix (a) and sheets (b). The plots depict the most prominent in basins of localization for ϕ and χ_1 dihedral rotors in TRP during secondary structure formation and breaking.

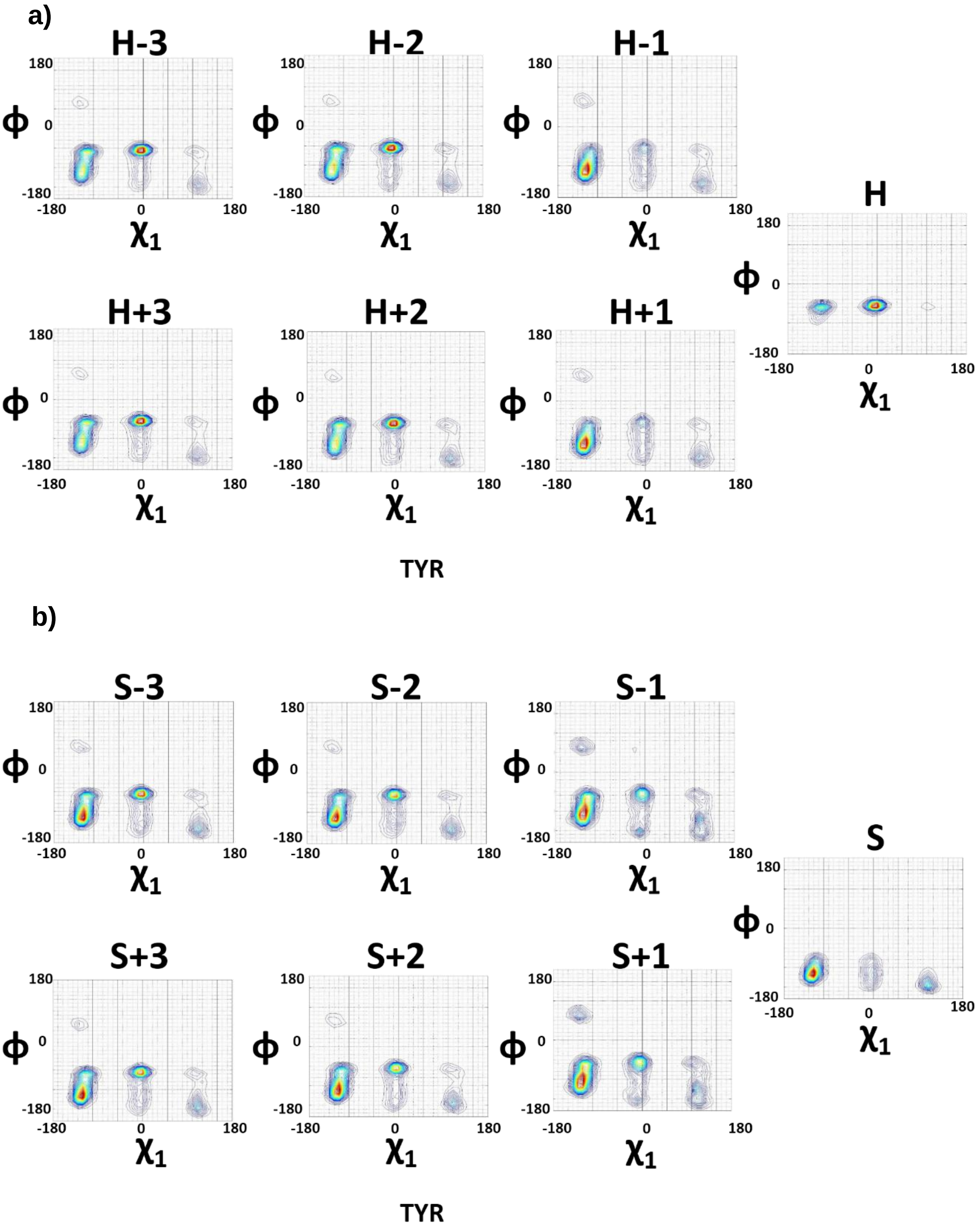

Fig A17: **Propensity of TYR in secondary structures.** ϕ vs χ_1 for helix (a) and sheets (b). The plots depict the most prominent in basins of localization for ϕ and χ_1 dihedral rotors in TYR during secondary structure formation and breaking.

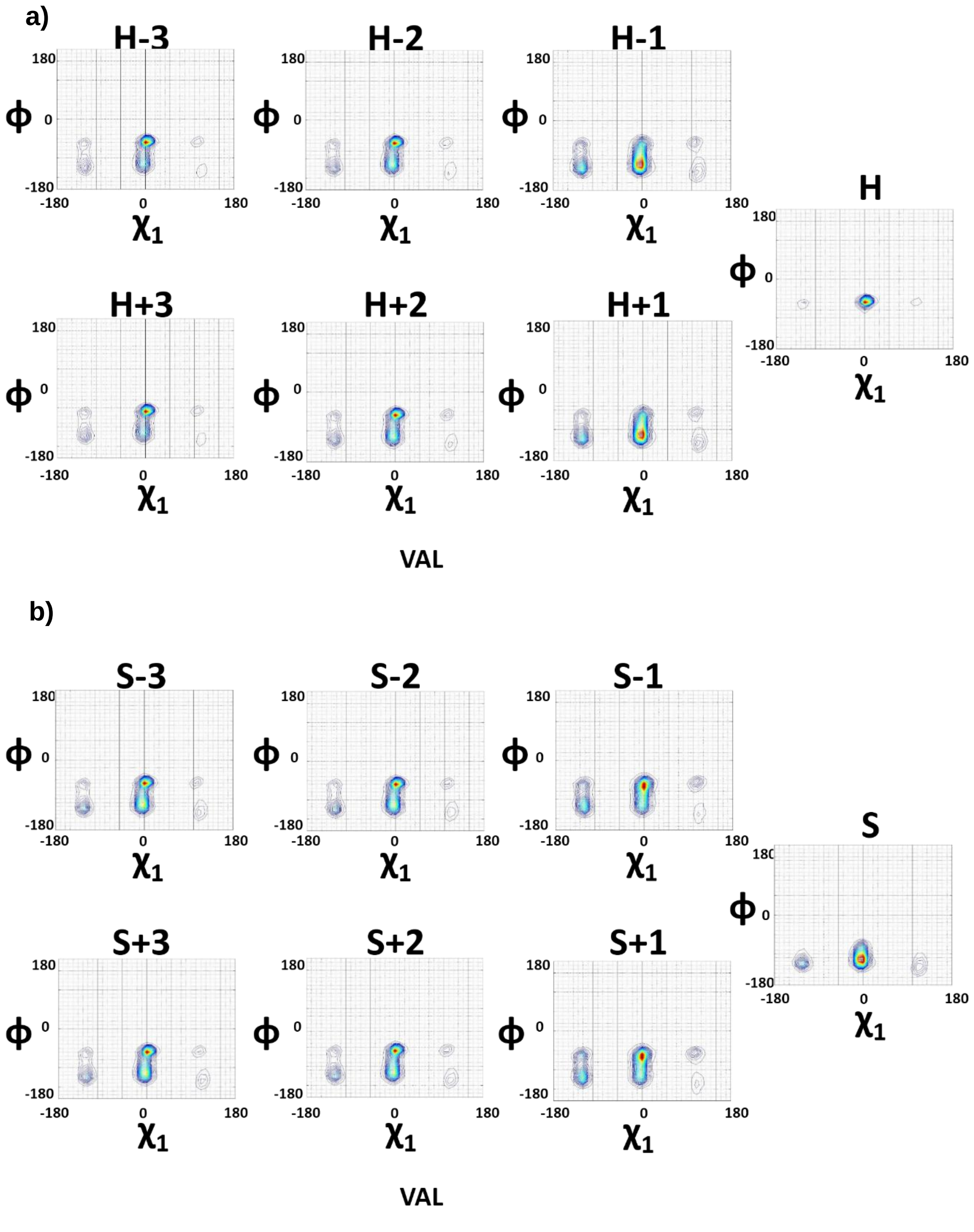

Fig A18: **Propensity of VAL in secondary structures.** ϕ vs χ_1 for helix (a) and sheets (b). The plots depict the most prominent in basins of localization for ϕ and χ_1 dihedral rotors in VAL during secondary structure formation and breaking.

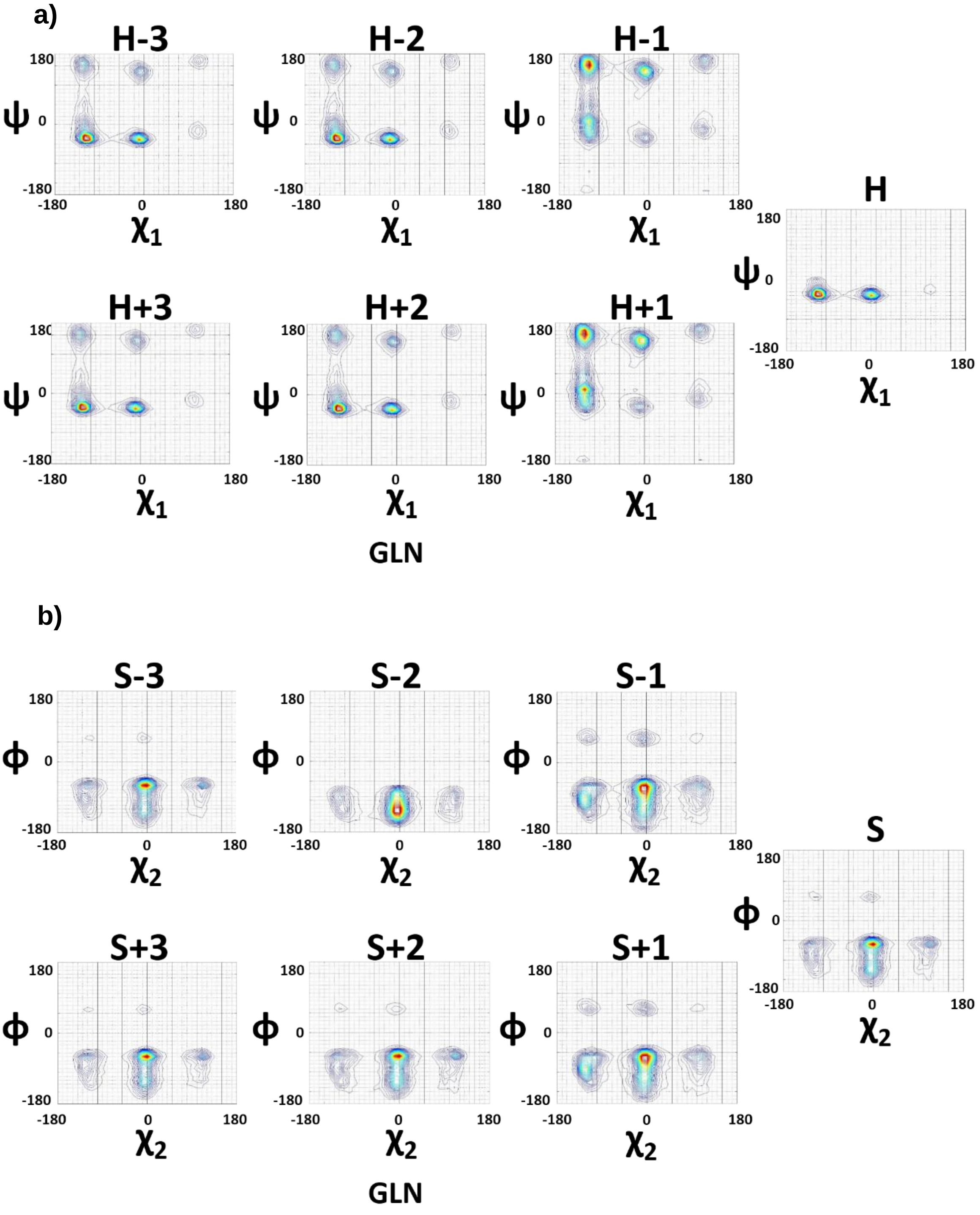

Fig A19: **Propensity of GLN in secondary structures.** ψ vs χ_1 for helix (a) and sheets (b). The plots depict the most prominent in basins of localization for ψ and χ_1 dihedral rotors in GLN during secondary structure formation and breaking.

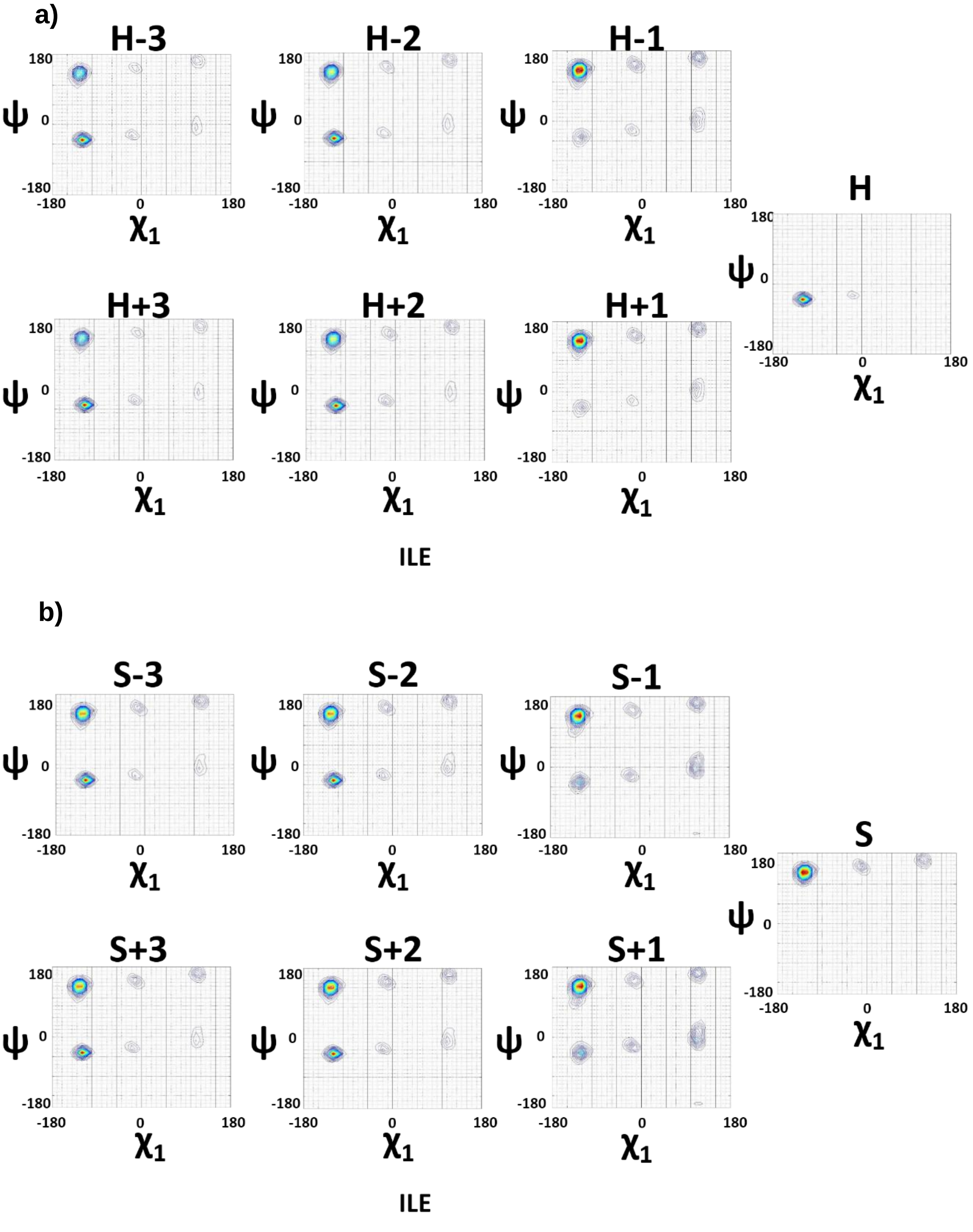

Fig A20: **Propensity of ILE in secondary structures.** ψ vs χ_1 for helix (a) and sheets (b). The plots depict the most prominent in basins of localization for ψ and χ_1 dihedral rotors in ILE during secondary structure formation and breaking.

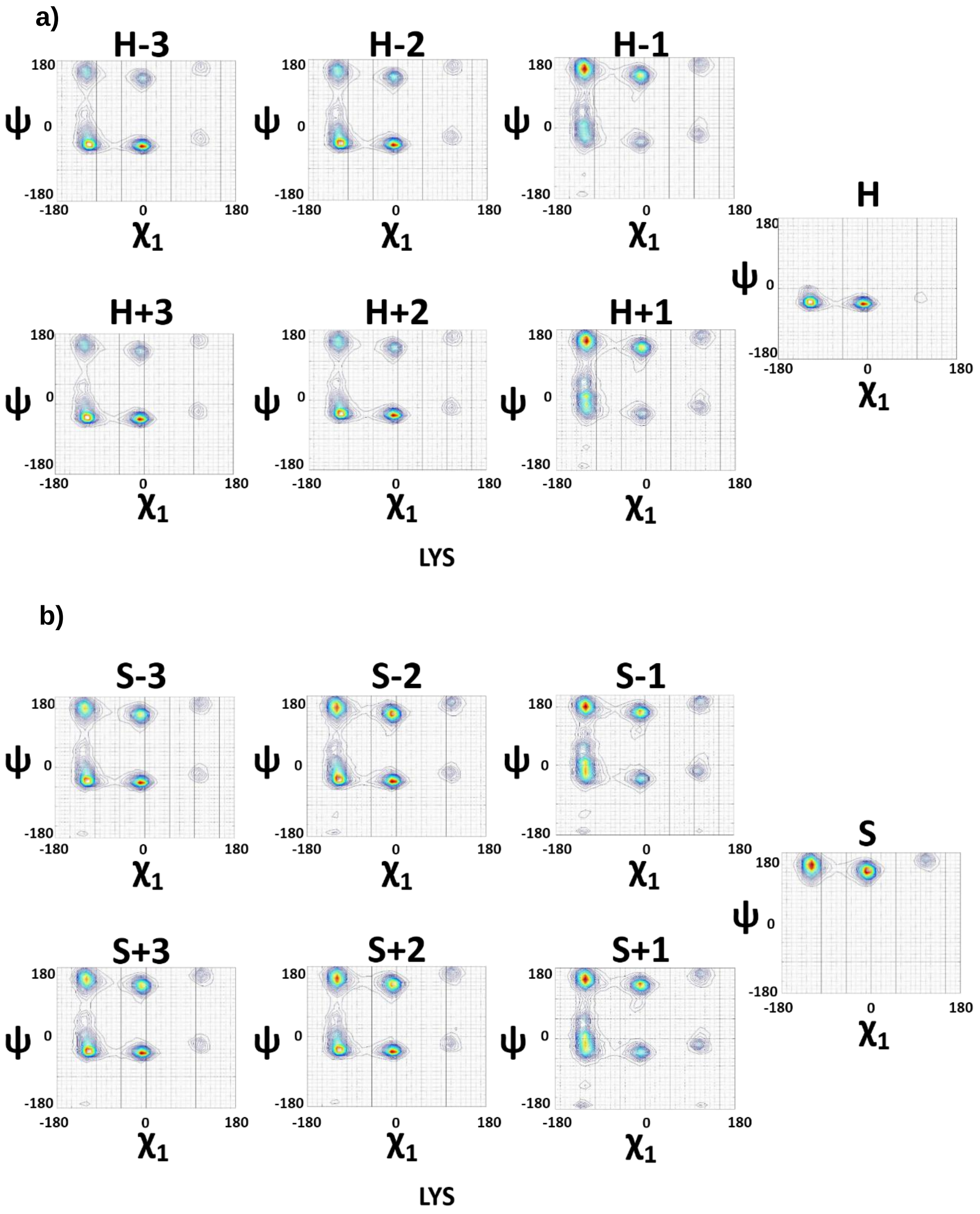

Fig A21: **Propensity of LYS in secondary structures.** ψ vs χ_1 for helix (a) and sheets (b). The plots depict the most prominent in basins of localization for ψ and χ_1 dihedral rotors in LYS during secondary structure formation and breaking.

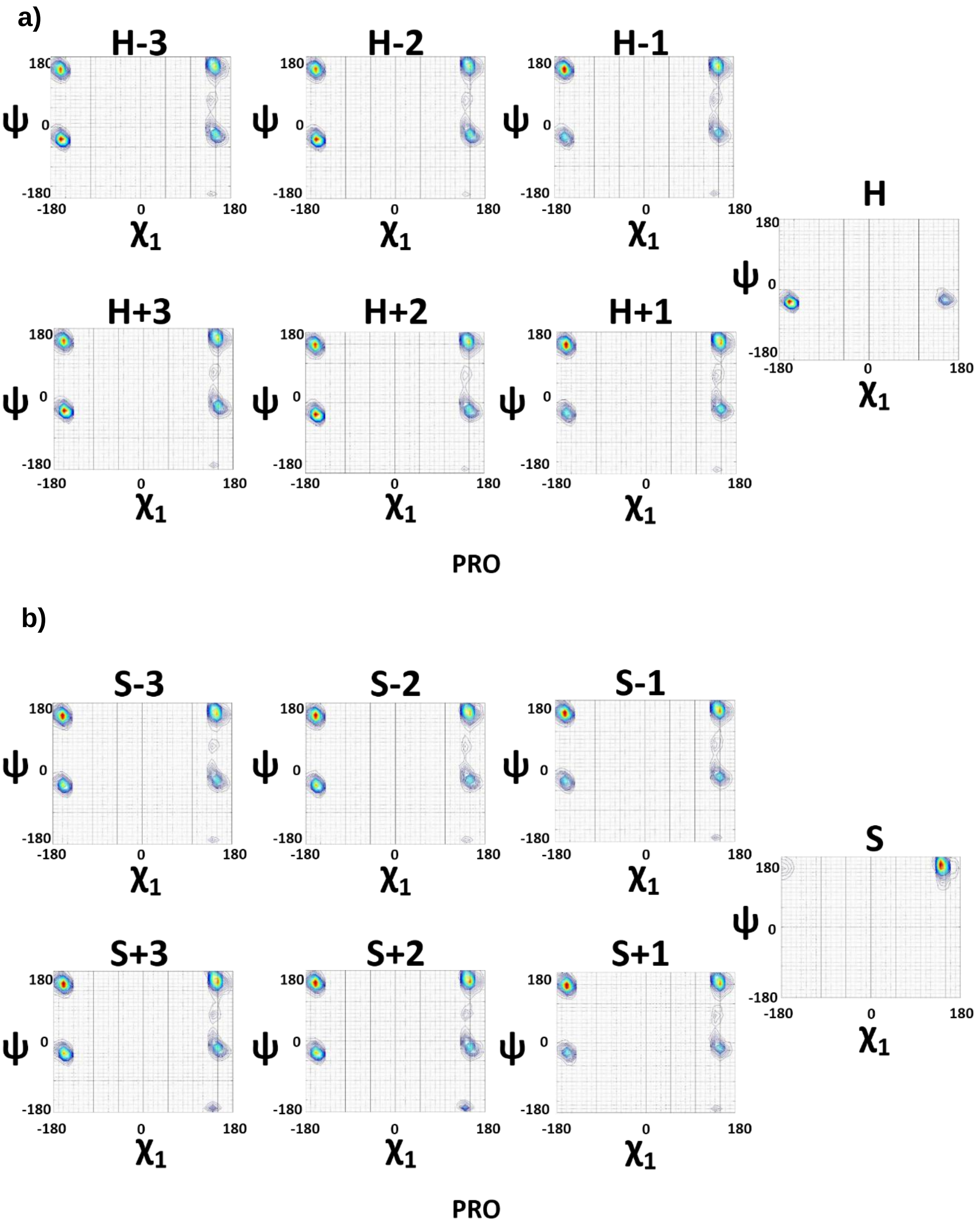

Fig A22: **Propensity of PRO in secondary structures.** ψ vs χ_1 for helix (a) and sheets (b). The plots depict the most prominent in basins of localization for ψ and χ_1 dihedral rotors in PRO during secondary structure formation and breaking.

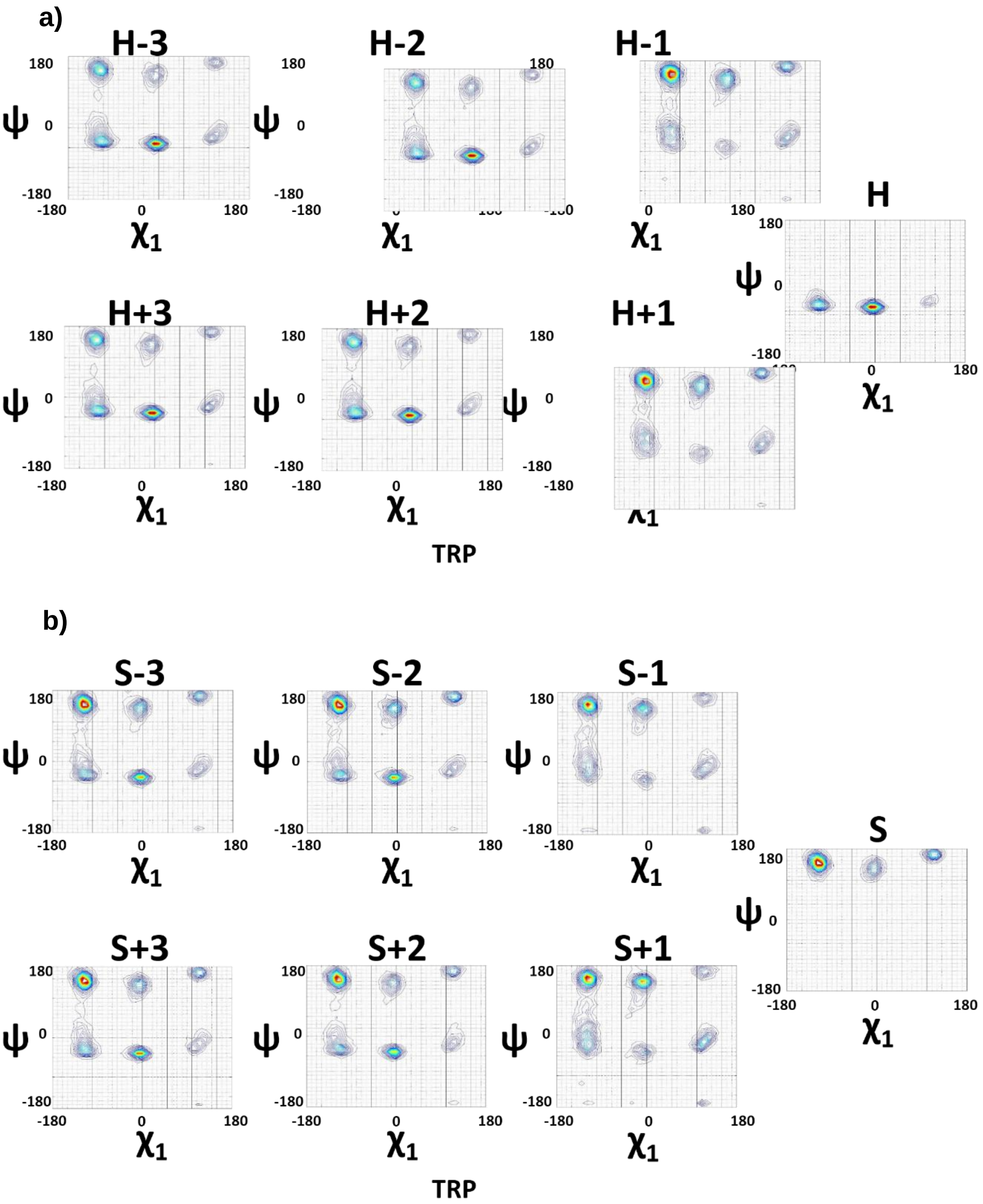

Fig A23: **Propensity of TRP in secondary structures.** ψ vs χ_1 for helix (a) and sheets (b). The plots depict the most prominent in basins of localization for ψ and χ_1 dihedral rotors in TRP during secondary structure formation and breaking.

a)

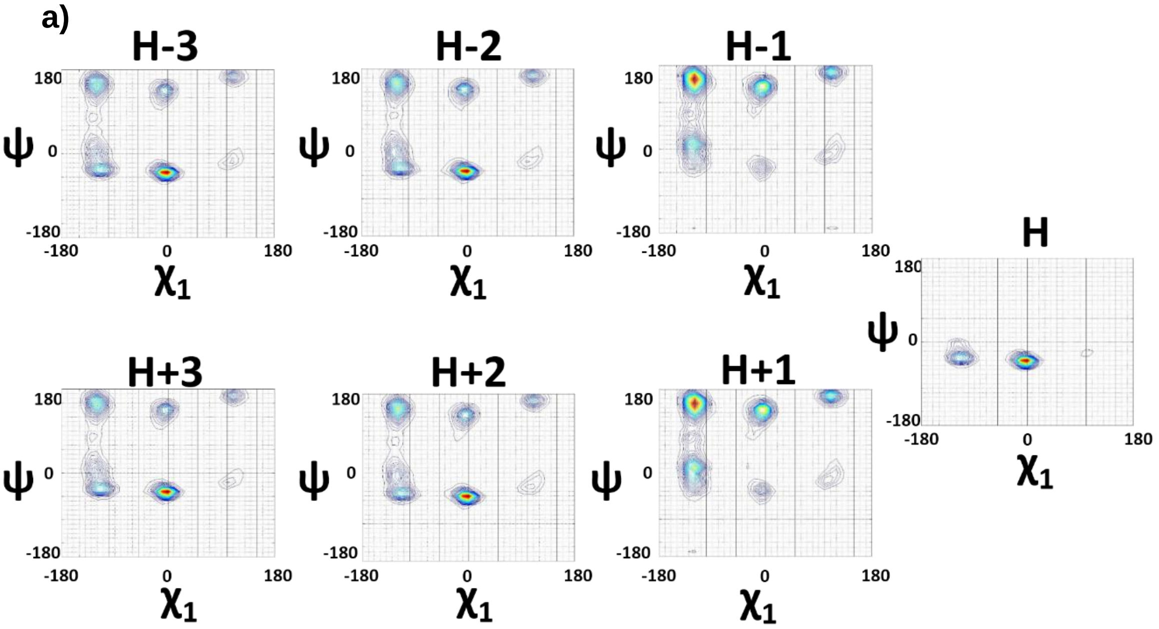

TYR

b)

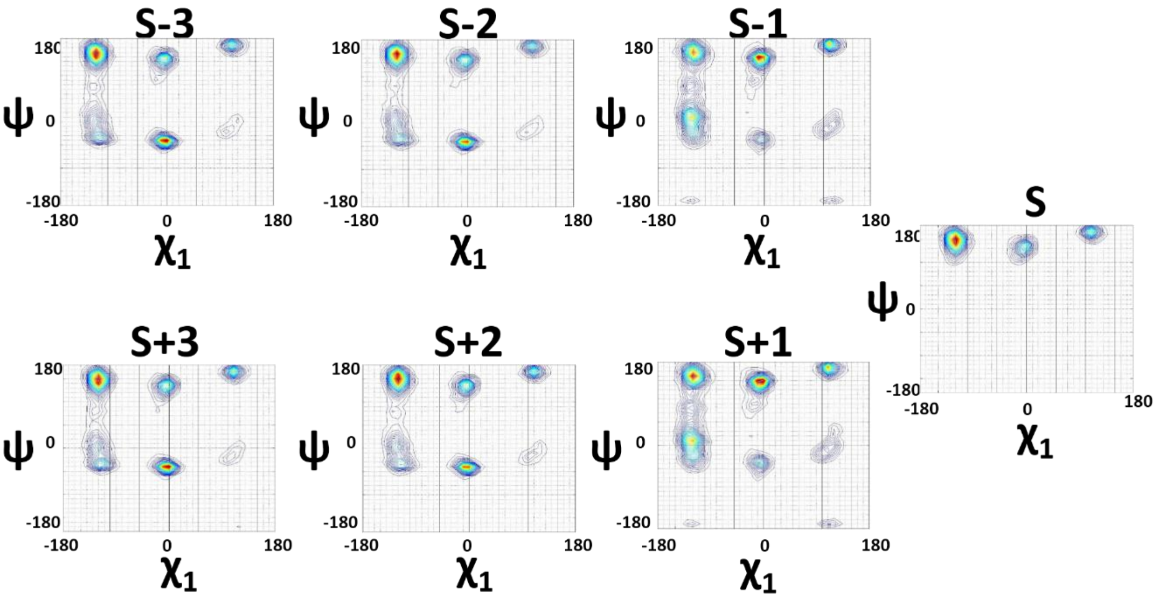

TYR

Fig A24: **Propensity of TYR in secondary structures.** ψ vs χ_1 for helix (a) and sheets (b). The plots depict the most prominent in basins of localization for ψ and χ_1 dihedral rotors in TYR during secondary structure formation and breaking.

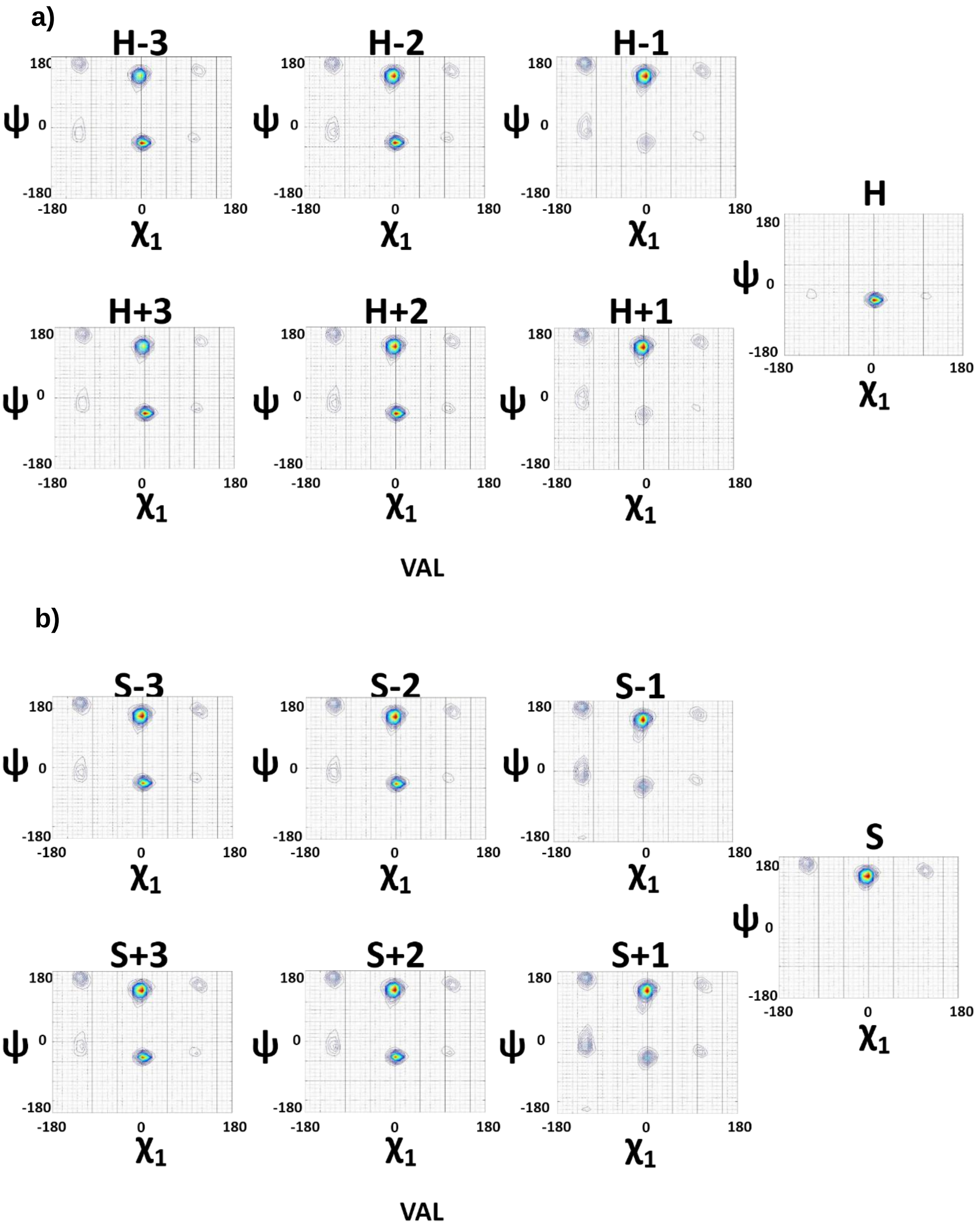

Fig A25: **Propensity of VAL in secondary structures.** ψ vs χ_1 for helix (a) and sheets (b). The plots depict the most prominent in basins of localization for ψ and χ_1 dihedral rotors in VAL during secondary structure formation and breaking.

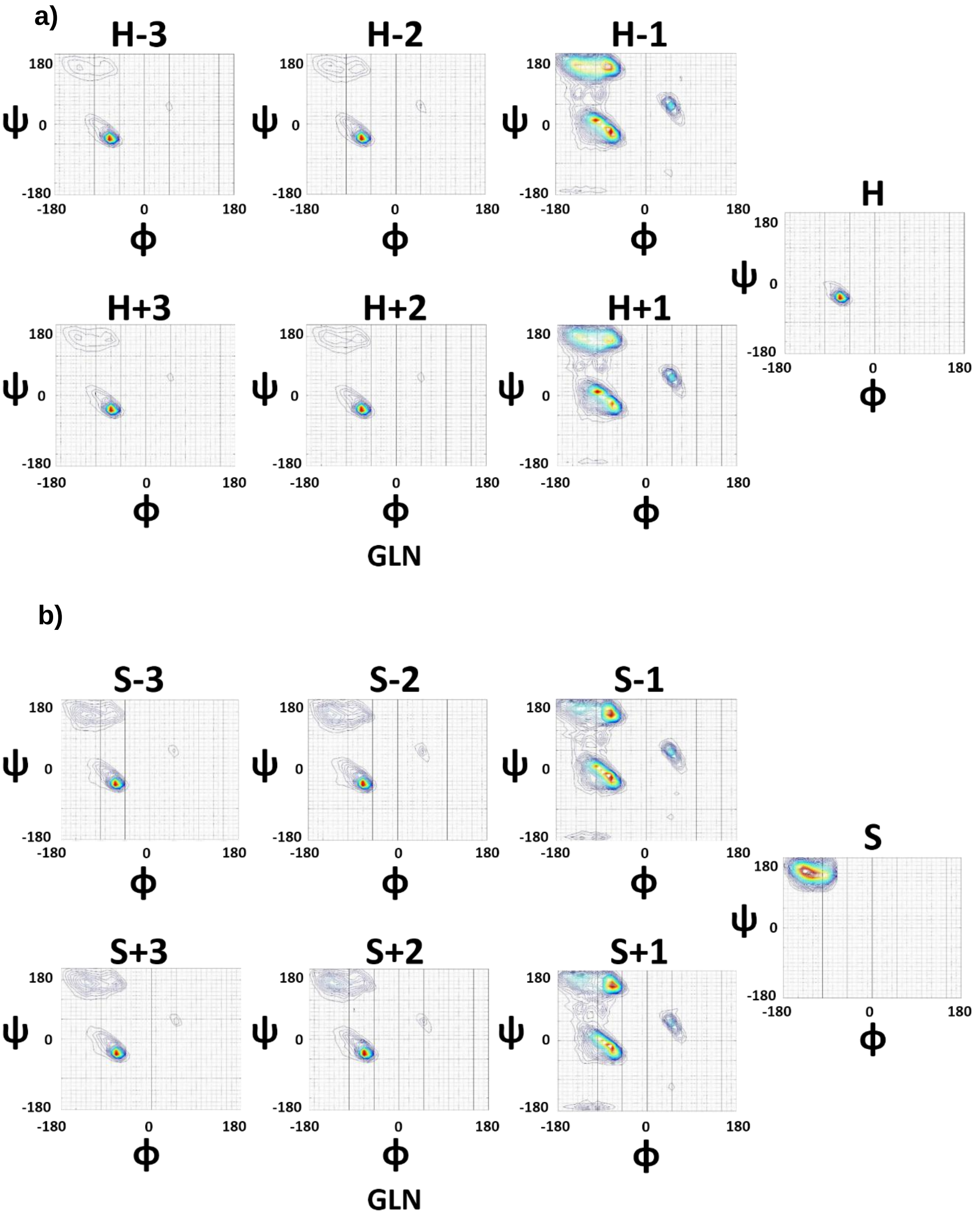

Fig A26: **Propensity of in GLN secondary structures.** : ψ vs ϕ for helix (a) and sheets (b). The plots depict the most prominent in basins of localization for ψ and ϕ dihedral rotors in GLN during secondary structure formation and breaking.

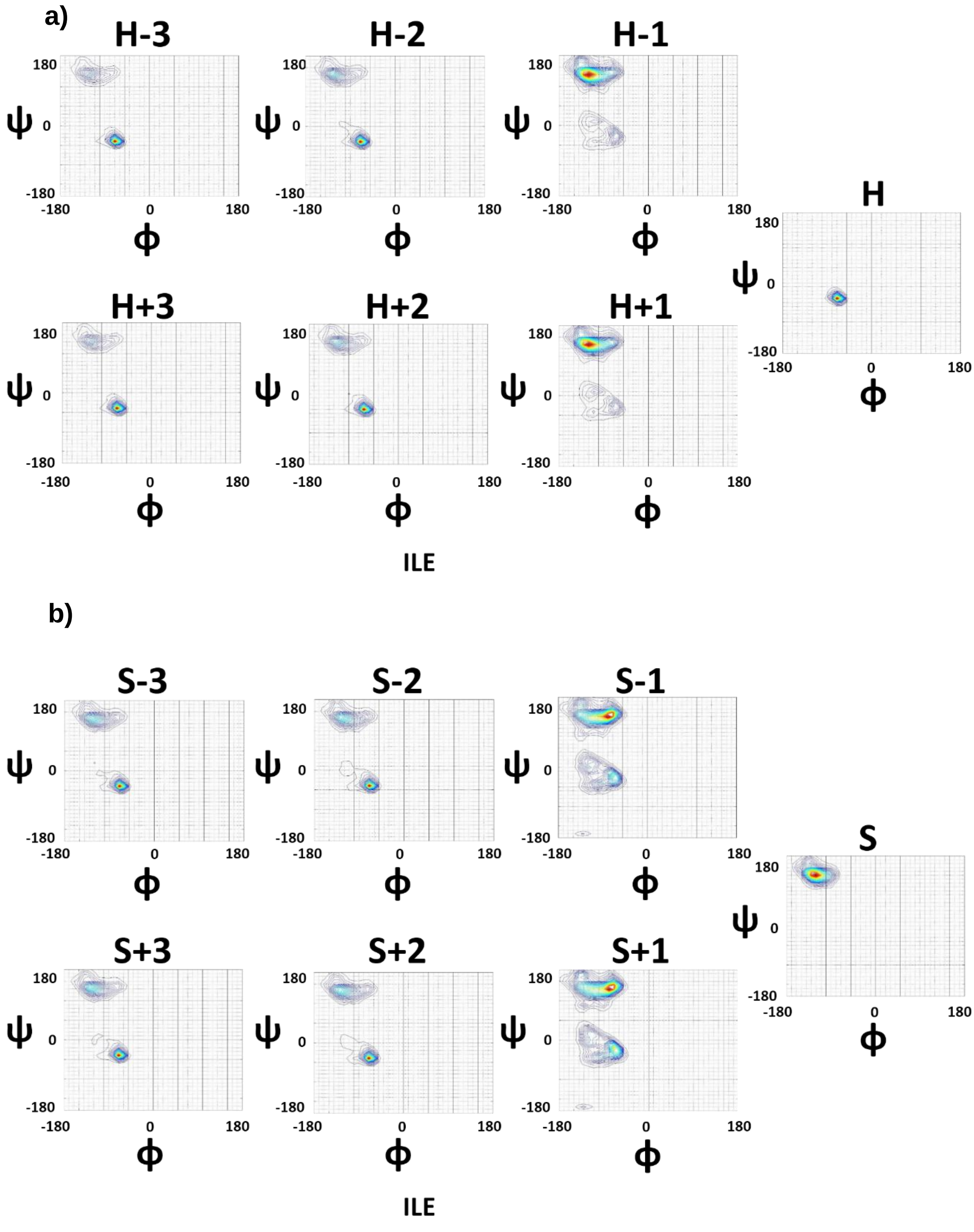

Fig A27: **Propensity of in ILE secondary structures.** : ψ vs ϕ for helix (a) and sheets (b). The plots depict the most prominent in basins of localization for ψ and ϕ dihedral rotors in ILE during secondary structure formation and breaking.

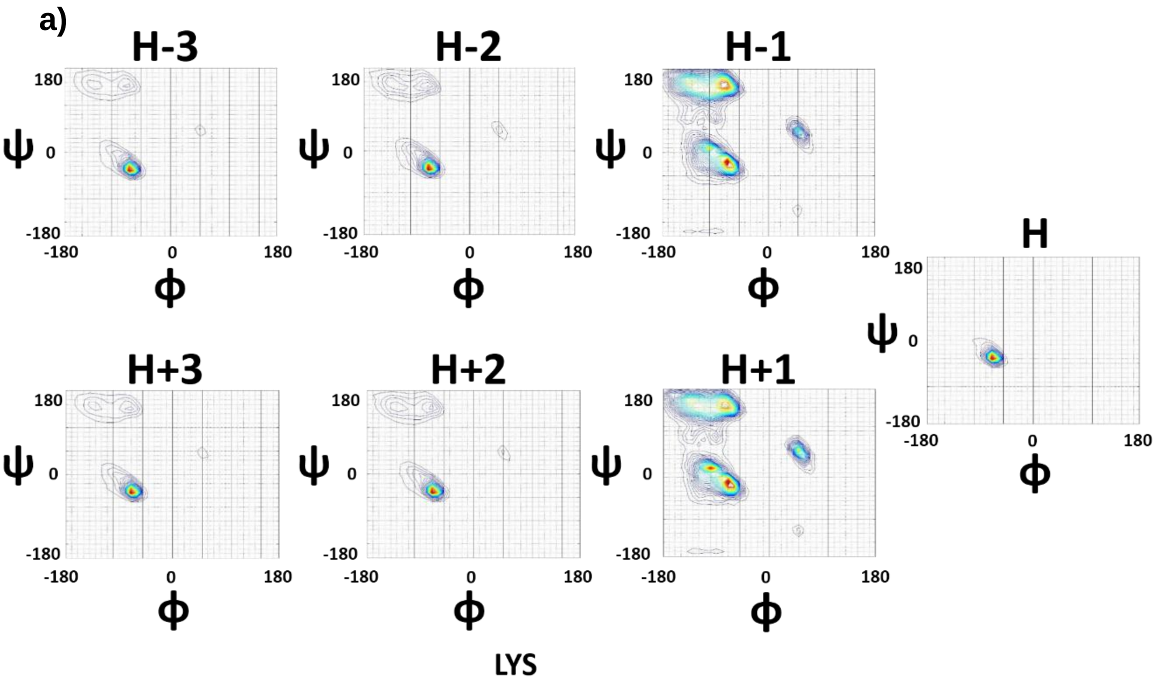

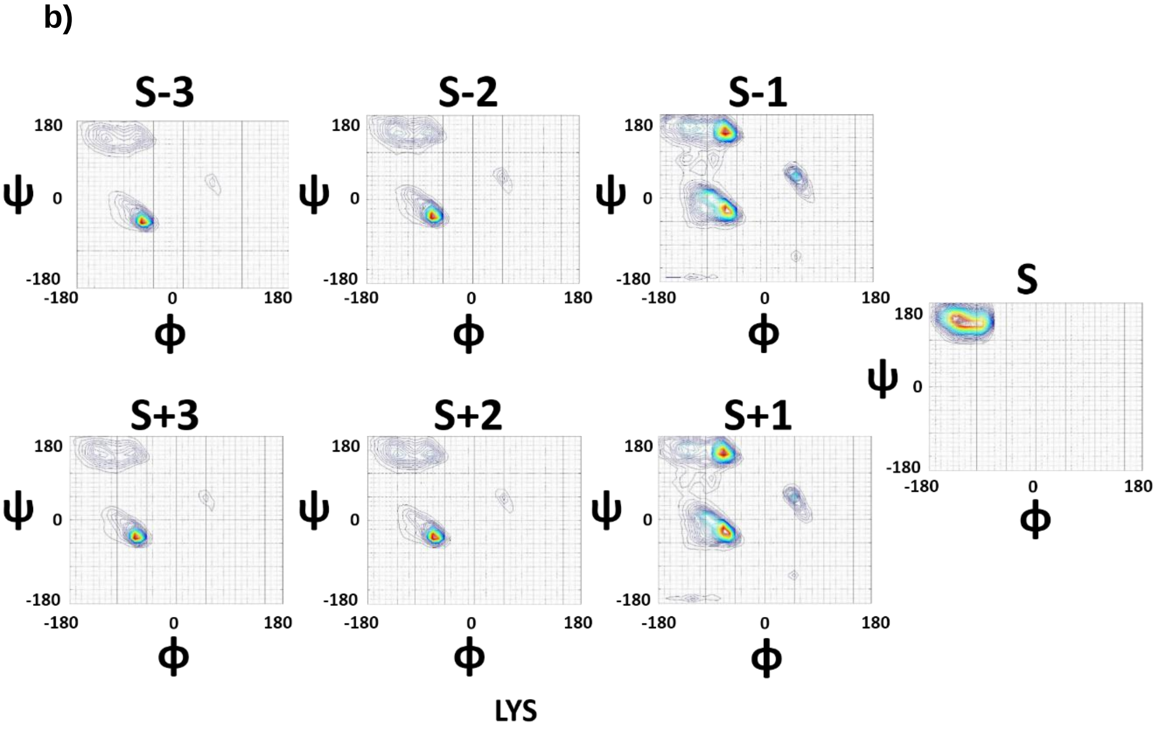

Fig A28: **Propensity of in LYS secondary structures.** : ψ vs ϕ for helix (a) and sheets (b). The plots depict the most prominent in basins of localization for ψ and ϕ dihedral rotors in LYS during secondary structure formation and breaking.

a)

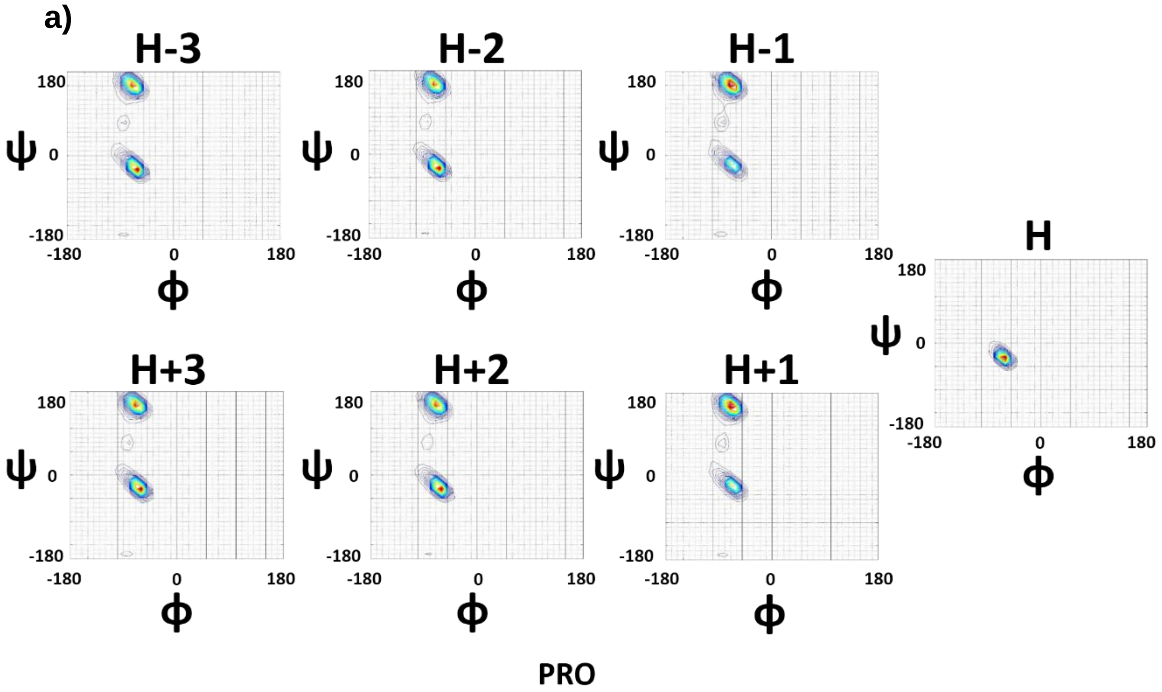

b)

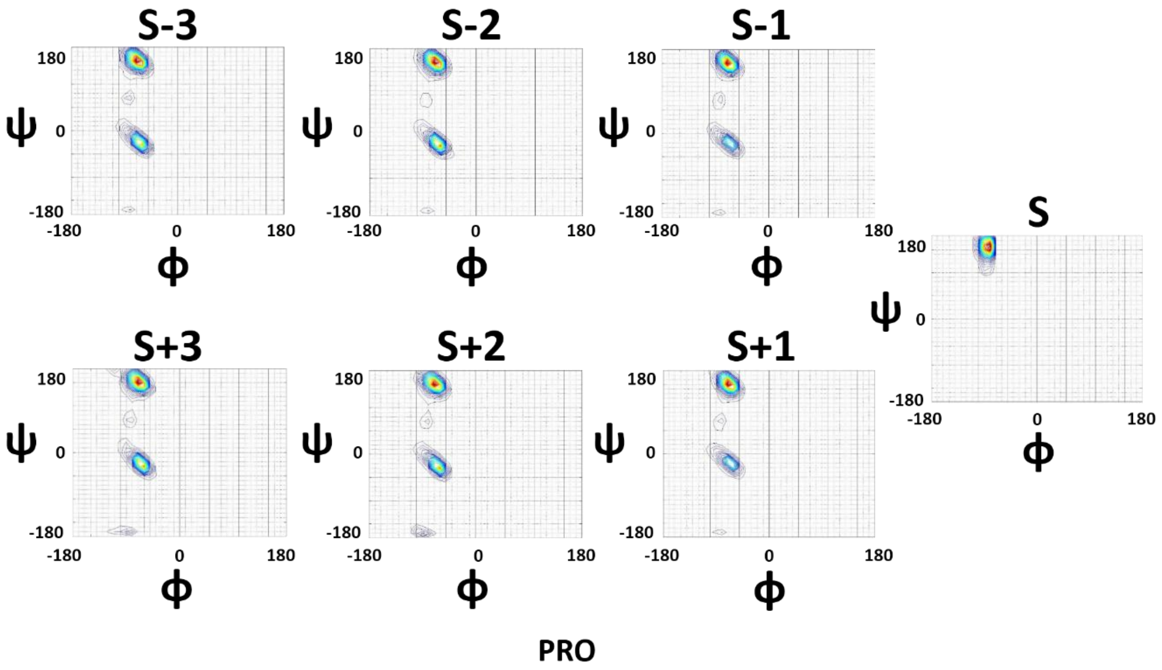

Fig A29: **Propensity of in PRO secondary structures.** : ψ vs ϕ for helix (a) and sheets (b). The plots depict the most prominent in basins of localization for ψ and ϕ dihedral rotors in PRO during secondary structure formation and breaking.

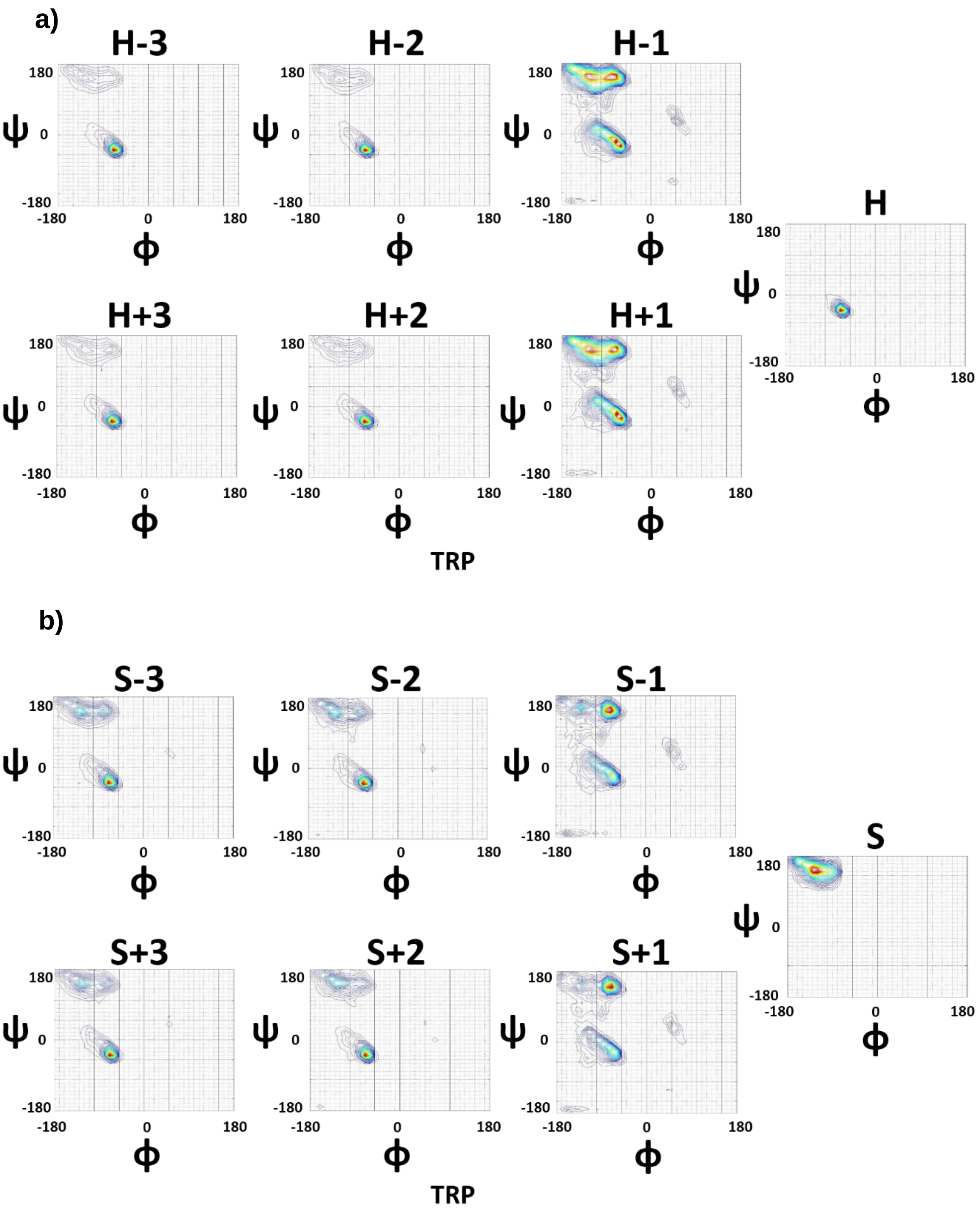

Fig A30: **Propensity of in TRP secondary structures.** : ψ vs ϕ for helix (a) and sheets (b). The plots depict the most prominent in basins of localization for ψ and ϕ dihedral rotors in TRP during secondary structure formation and breaking.

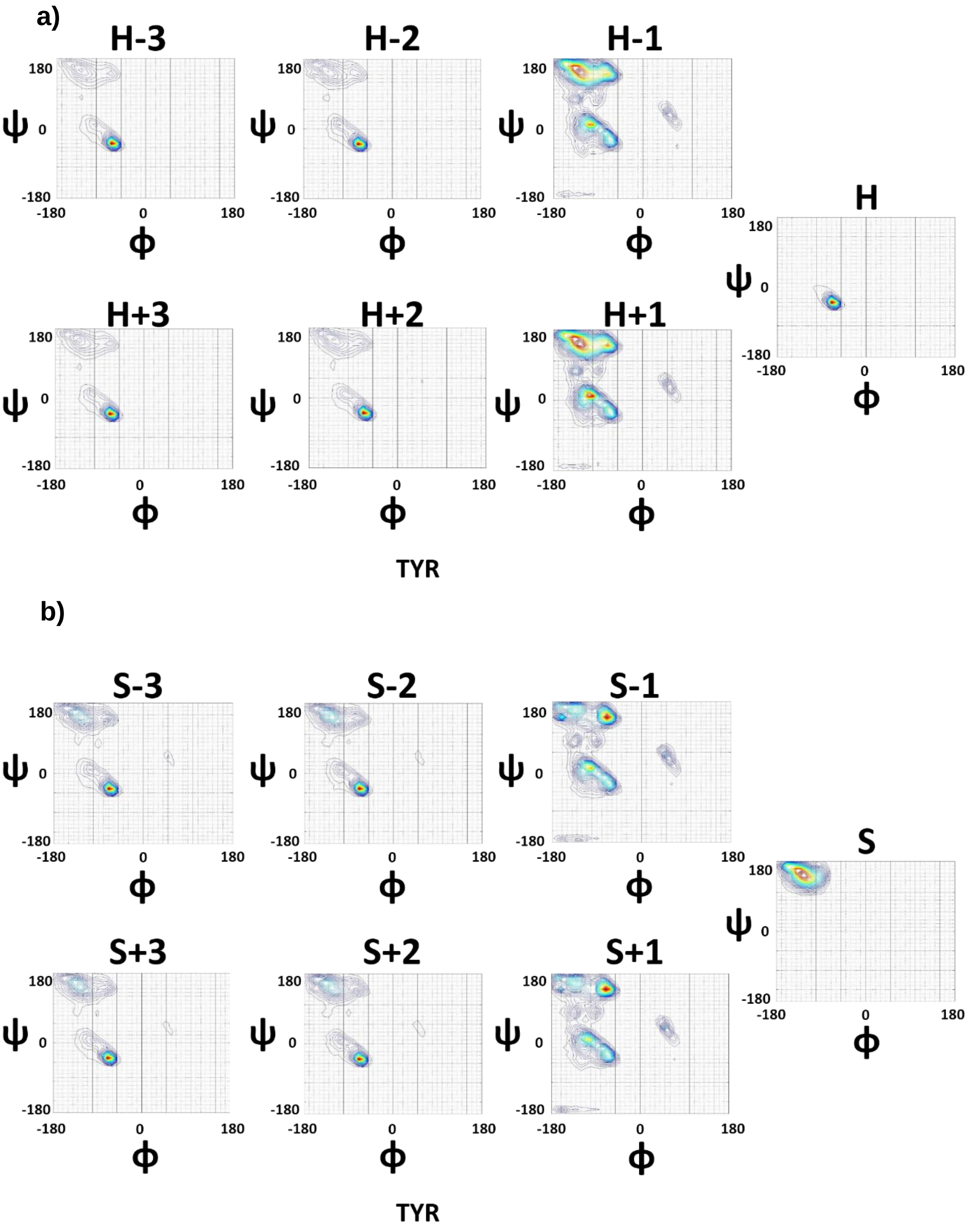

Fig A31: **Propensity of in TYR secondary structures.** : ψ vs ϕ for helix (a) and sheets (b). The plots depict the most prominent in basins of localization for ψ and ϕ dihedral rotors in TYR during secondary structure formation and breaking.

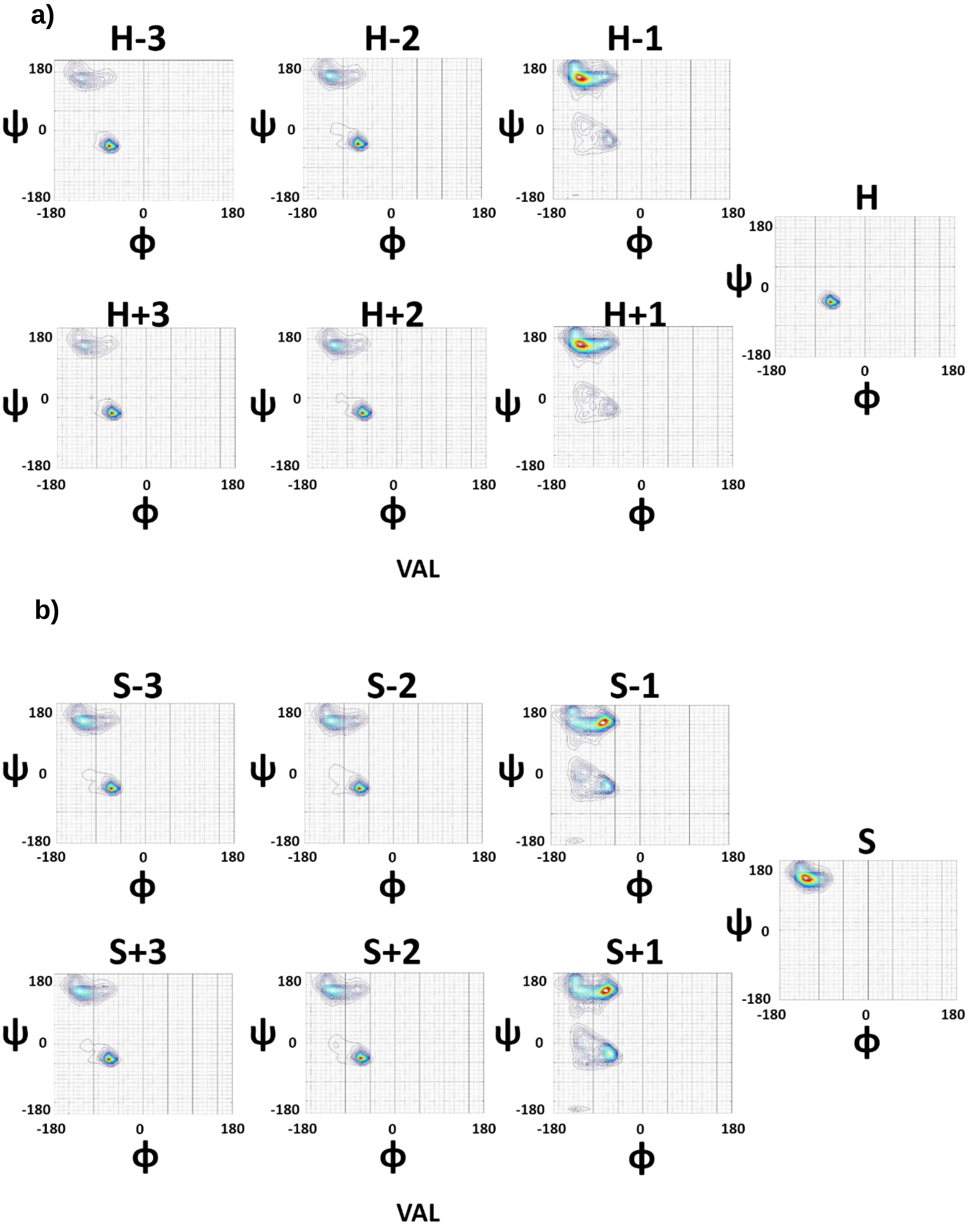

Fig A32: **Propensity of in VAL secondary structures.** : ψ vs ϕ for helix (a) and sheets (b). The plots depict the most prominent in basins of localization for ψ and ϕ dihedral rotors in VAL during secondary structure formation and breaking.

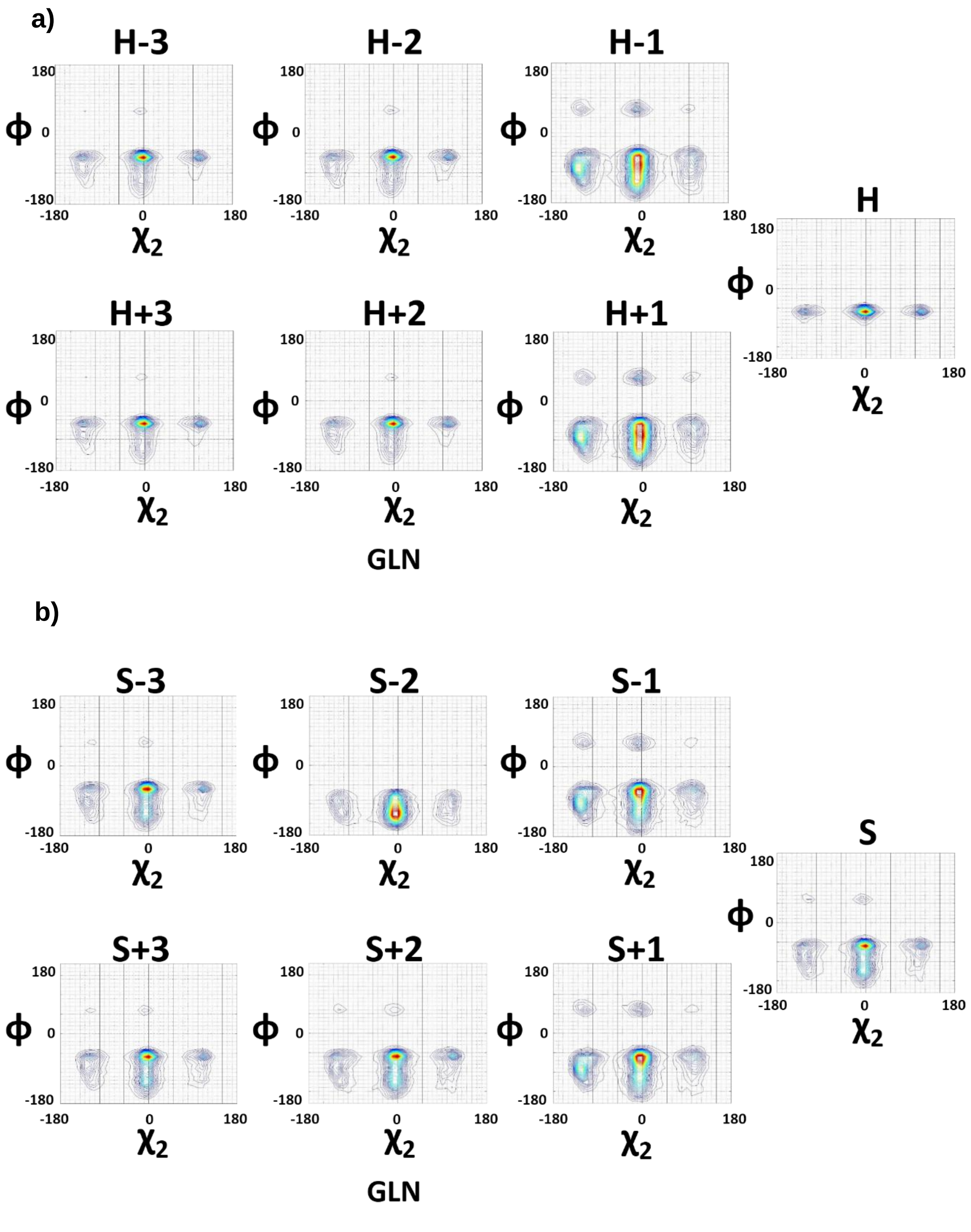

Fig A33: **Propensity of GLN in secondary structures.** : ϕ vs χ_2 for helix (a) and sheets (b). The plots depict the most prominent in basins of localization for : ϕ and χ_2 dihedral rotors in GLN during secondary structure formation and breaking.

a)

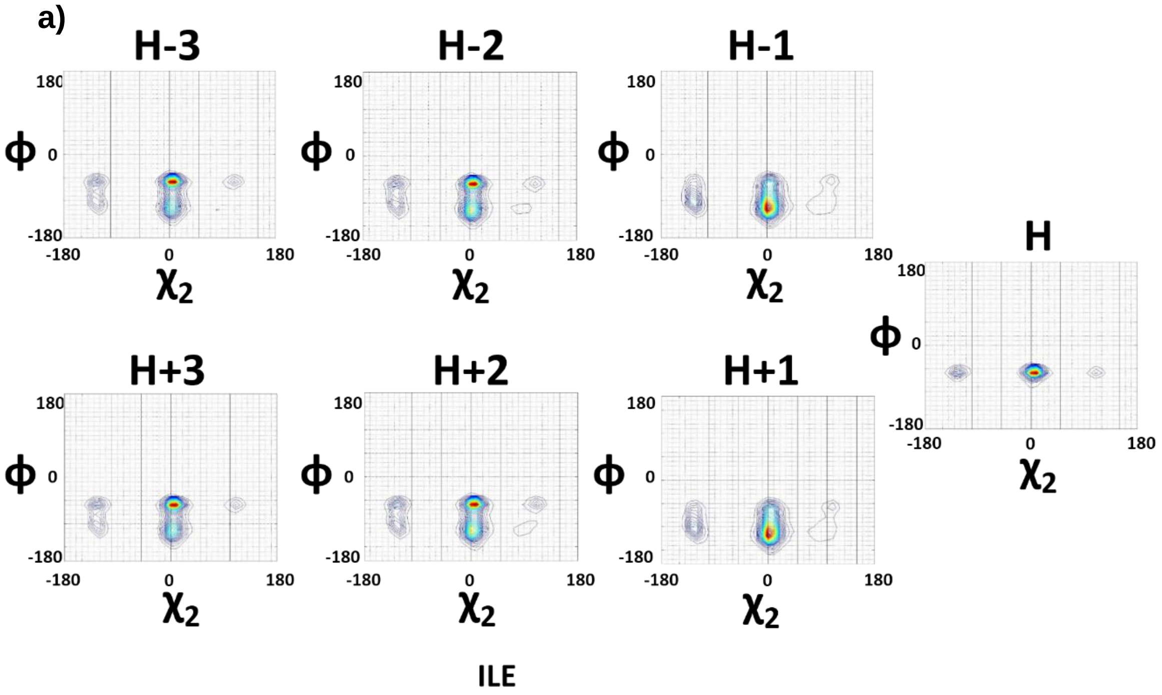

b)

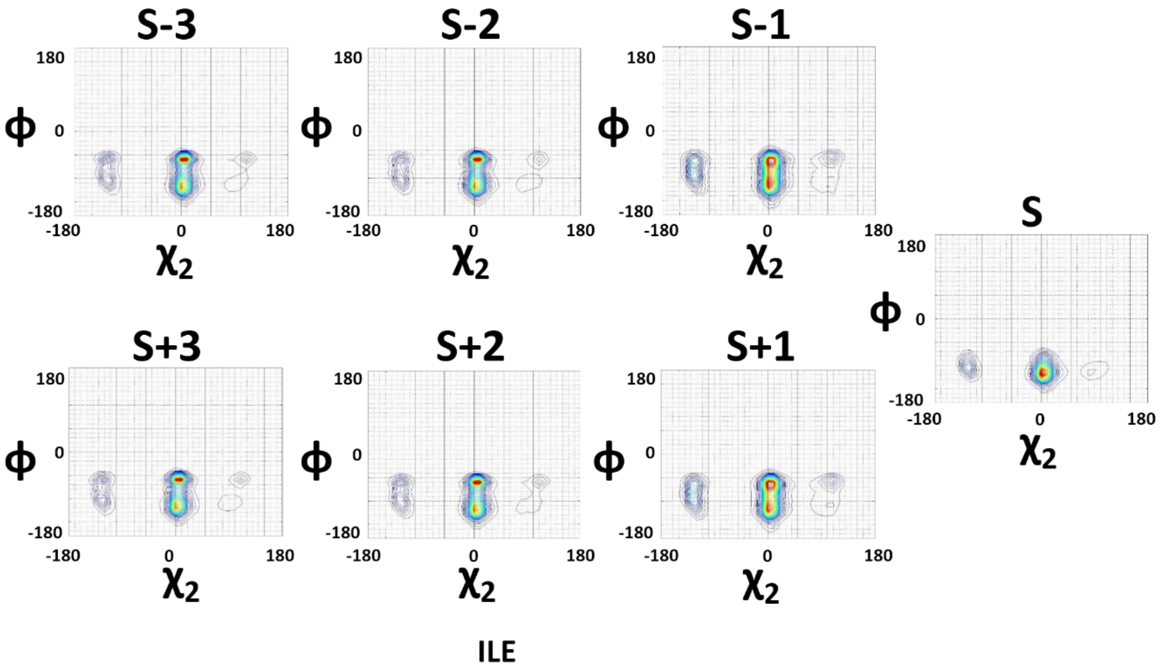

Fig A34: **Propensity of ILE in secondary structures.** : ϕ vs χ_2 for helix (a) and sheets (b). The plots depict the most prominent in basins of localization for : ϕ and χ_2 dihedral rotors in ILE during secondary structure formation and breaking.

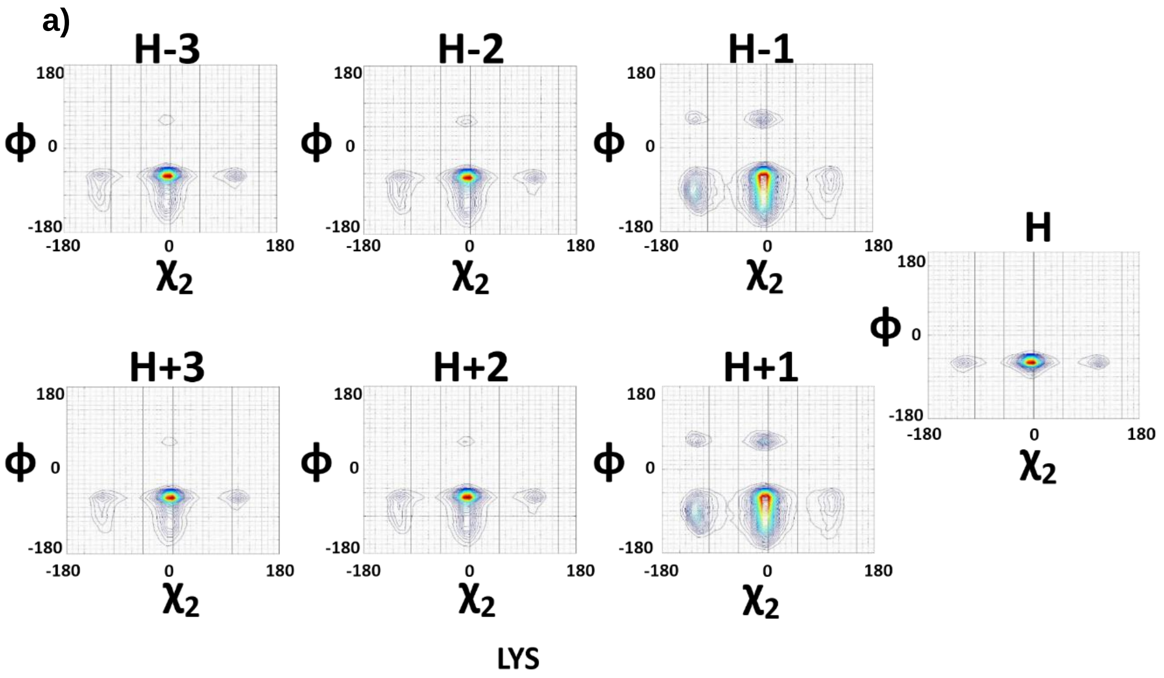

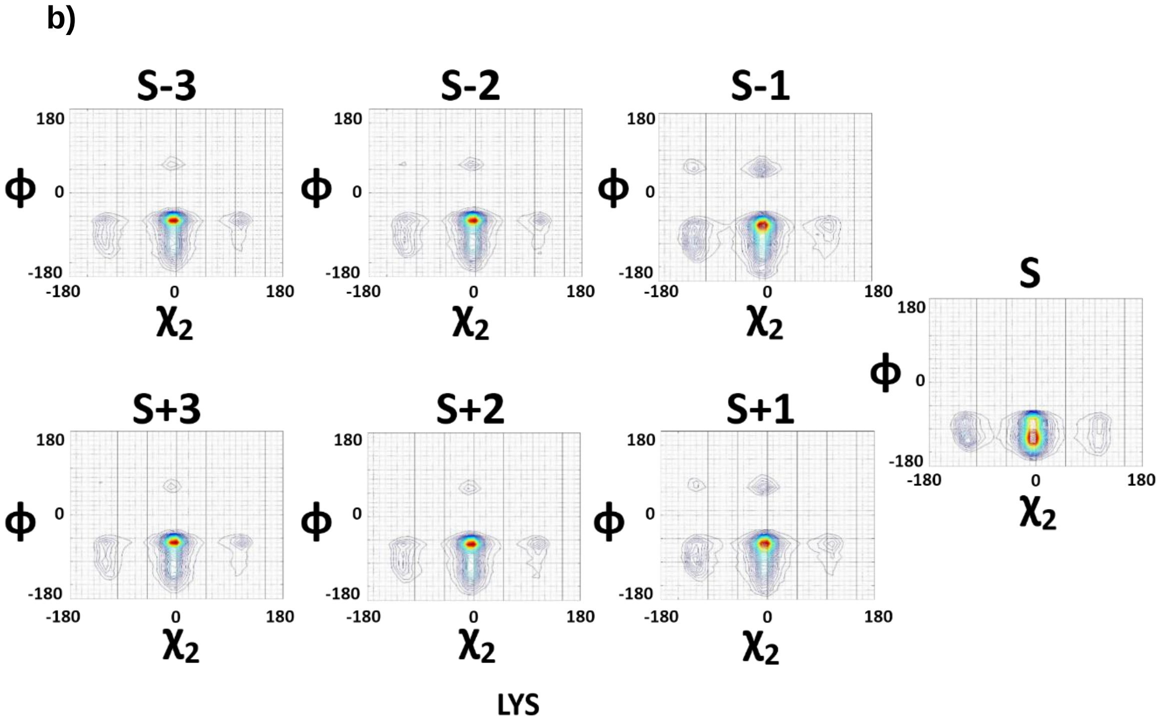

Fig A35: **Propensity of LYS in secondary structures.** : ϕ vs χ_2 for helix (a) and sheets (b). The plots depict the most prominent in basins of localization for : ϕ and χ_2 dihedral rotors in LYS during secondary structure formation and breaking.

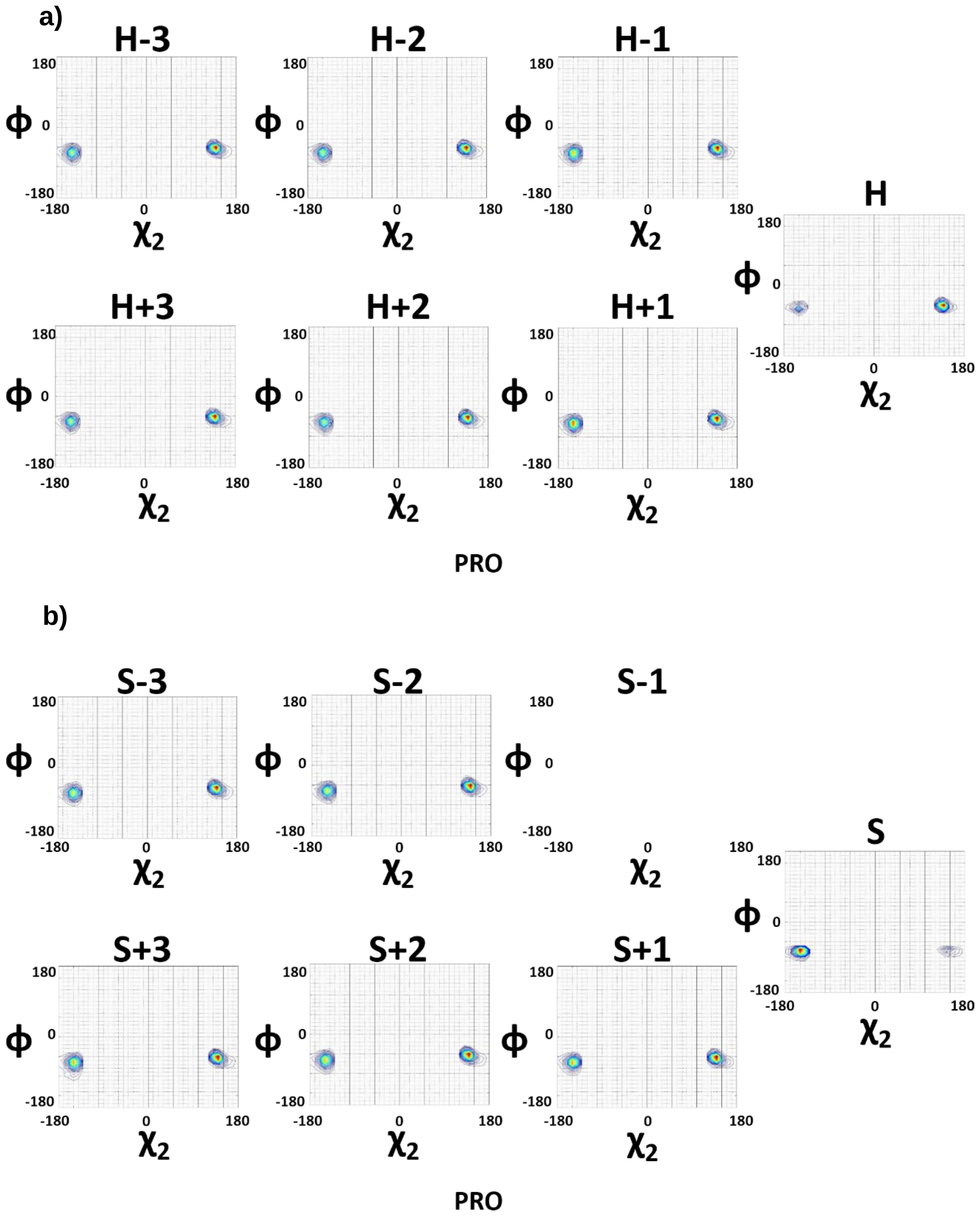

Fig A36: **Propensity of PRO in secondary structures.** : ϕ vs χ_2 for helix (a) and sheets (b). The plots depict the most prominent in basins of localization for ϕ and χ_2 dihedral rotors in PRO during secondary structure formation and breaking.

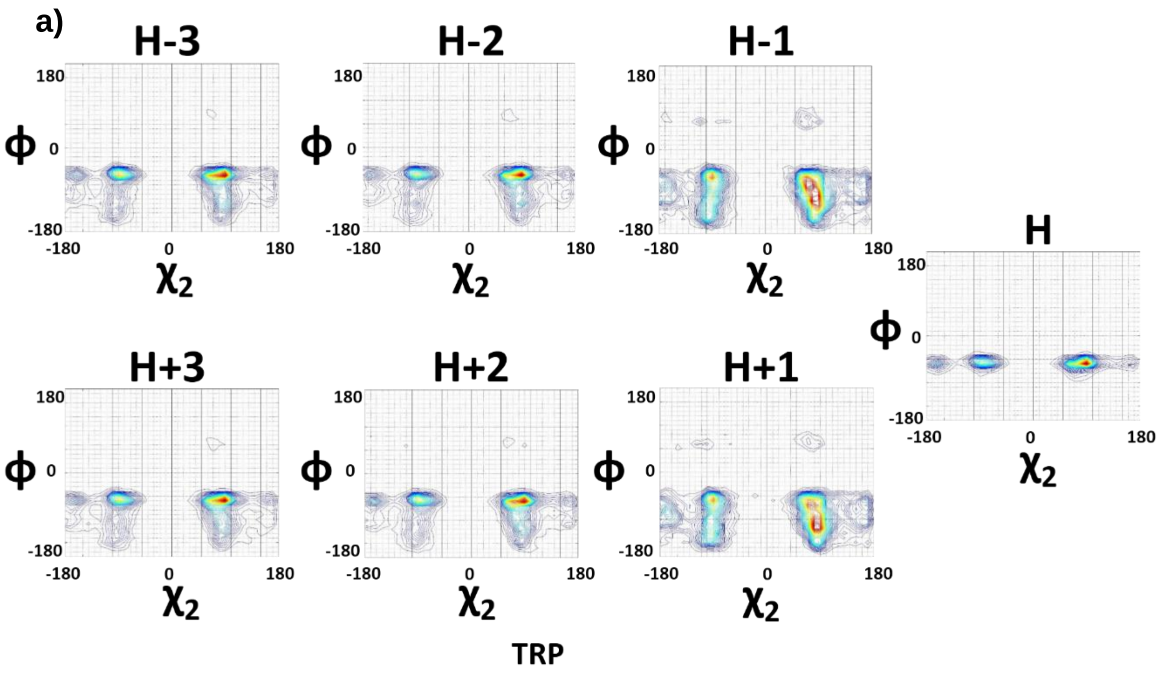

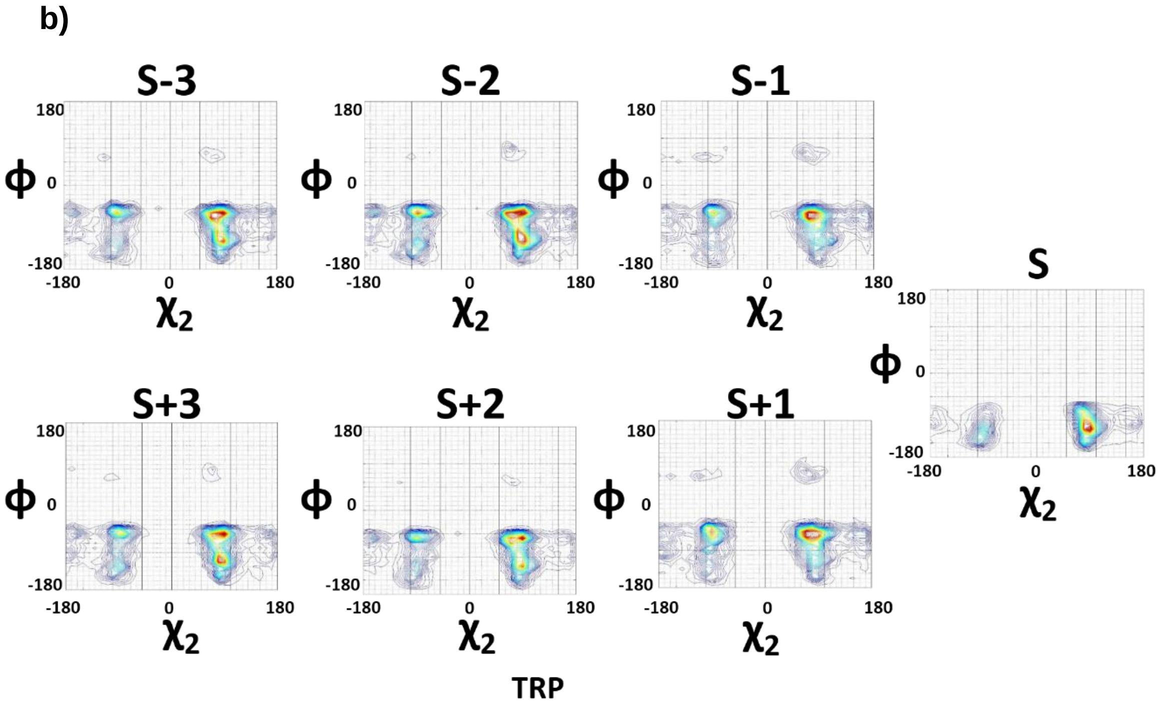

Fig A37: **Propensity of TRP in secondary structures.** : ϕ vs χ_2 for helix (a) and sheets (b). The plots depict the most prominent in basins of localization for ϕ and χ_2 dihedral rotors in TRP during secondary structure formation and breaking.

a)

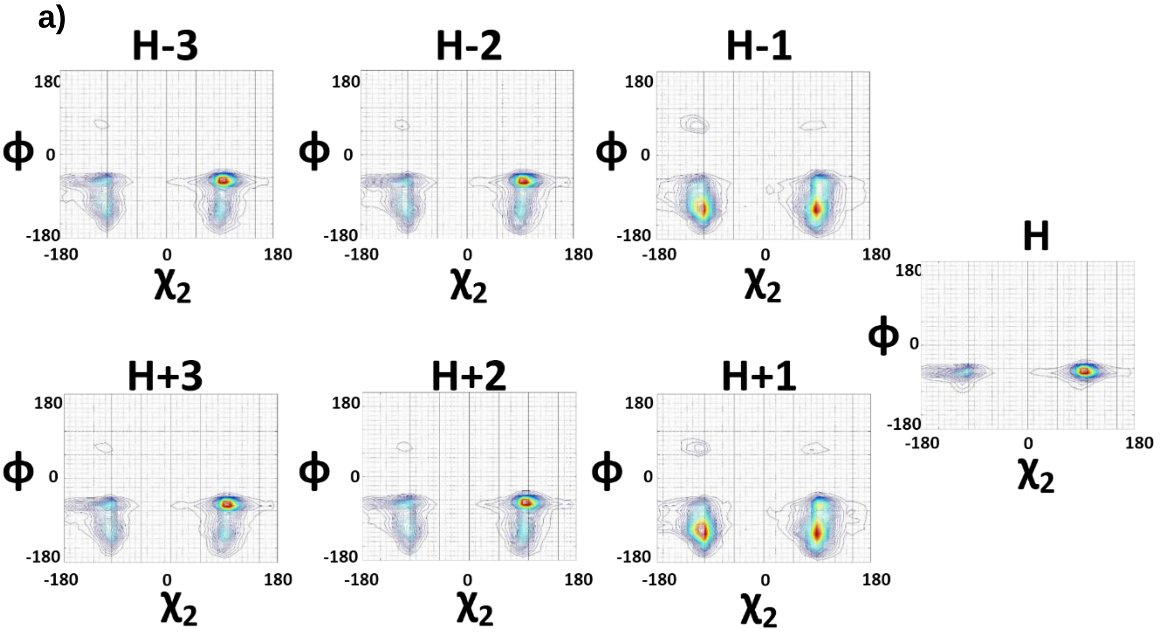

TYR

b)

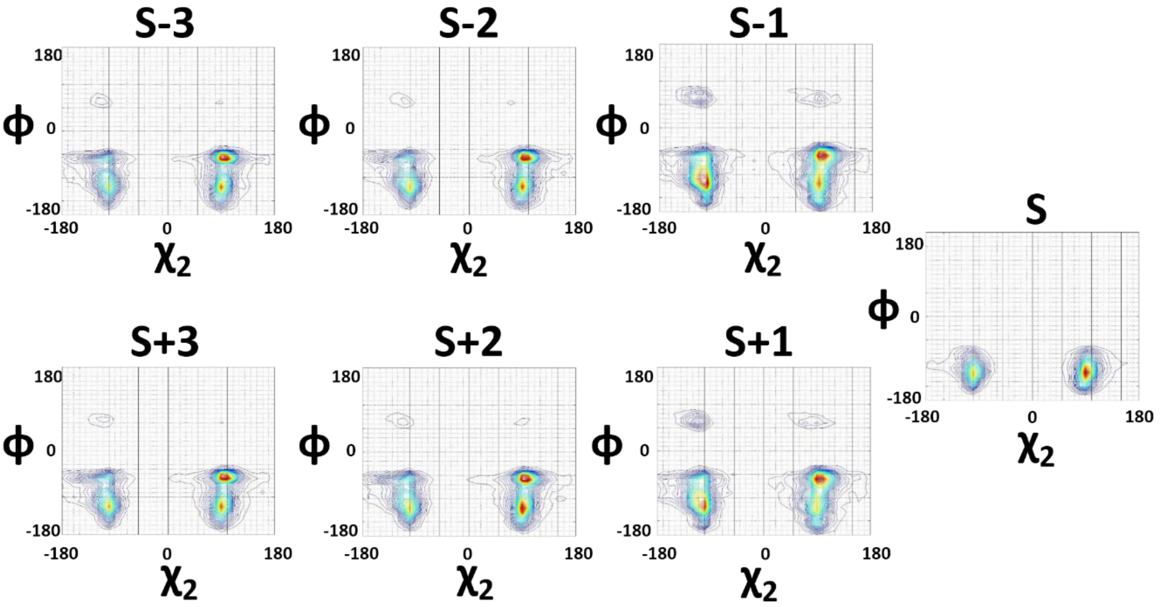

TYR

Fig A38: **Propensity of TYR in secondary structures.** : ϕ vs χ_2 for helix (a) and sheets (b). The plots depict the most prominent in basins of localization for ϕ and χ_2 dihedral rotors in TYR during secondary structure formation and breaking.

a)

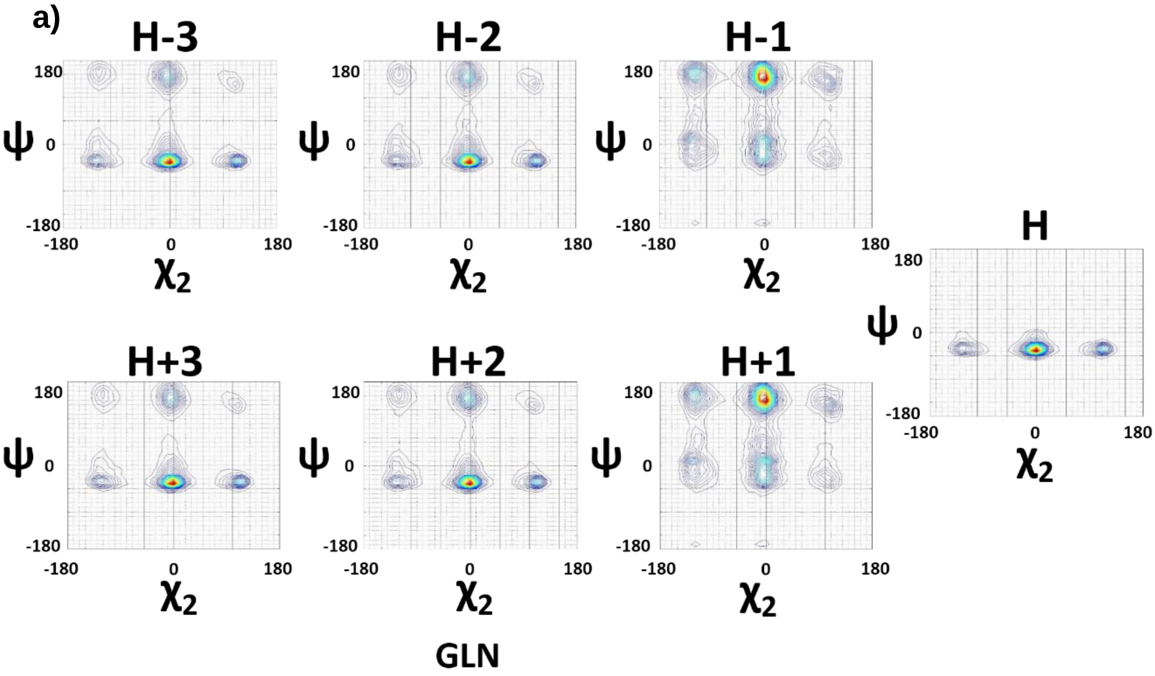

b)

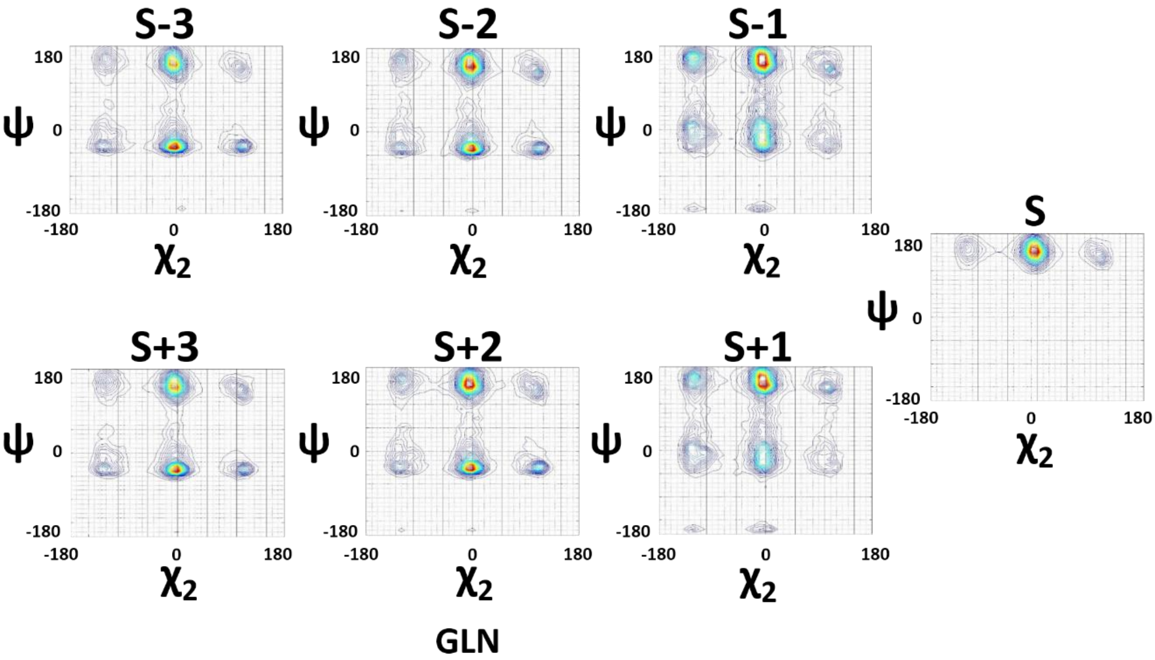

Fig A39: **Propensity of GLN in secondary structures.** : ψ vs χ_2 for helix (a) and sheets (b). The plots depict the most prominent in basins of localization for ψ and χ_2 dihedral rotors in GLN during secondary structure formation and breaking.

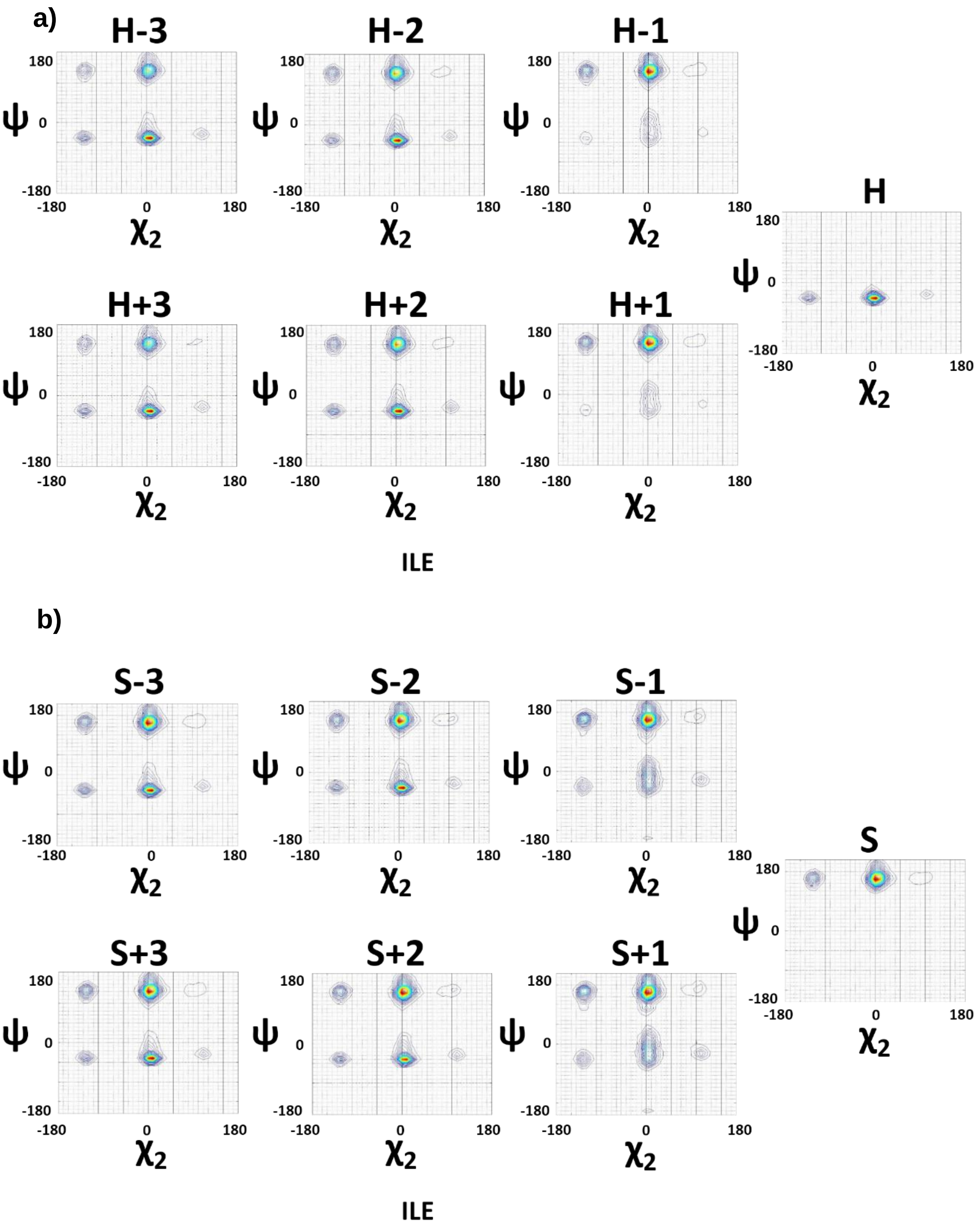

Fig A40: **Propensity of ILE in secondary structures.** : ψ vs χ_2 for helix (a) and sheets (b). The plots depict the most prominent in basins of localization for ψ and χ_2 dihedral rotors in ILE during secondary structure formation and breaking.

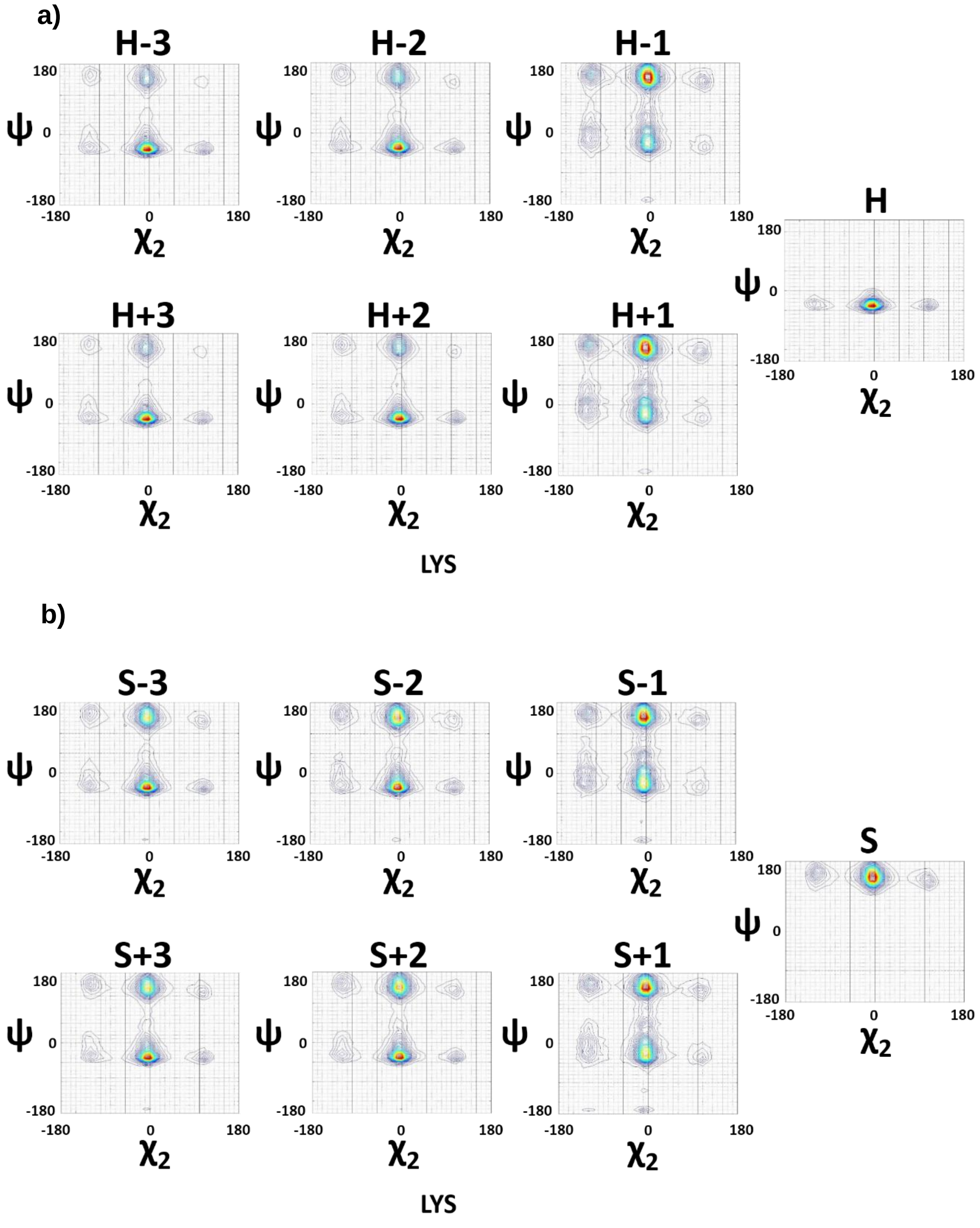

Fig A41: **Propensity of LYS in secondary structures.** : ψ vs χ_2 for helix (a) and sheets (b). The plots depict the most prominent in basins of localization for ψ and χ_2 dihedral rotors in LYS during secondary structure formation and breaking.

a)

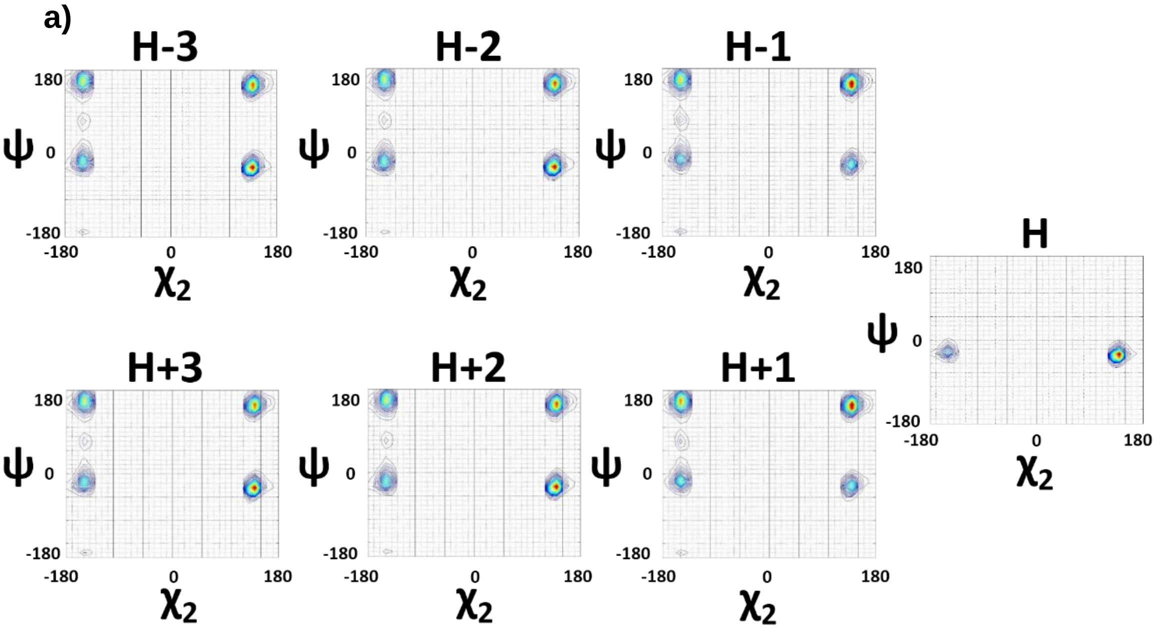

PRO

b)

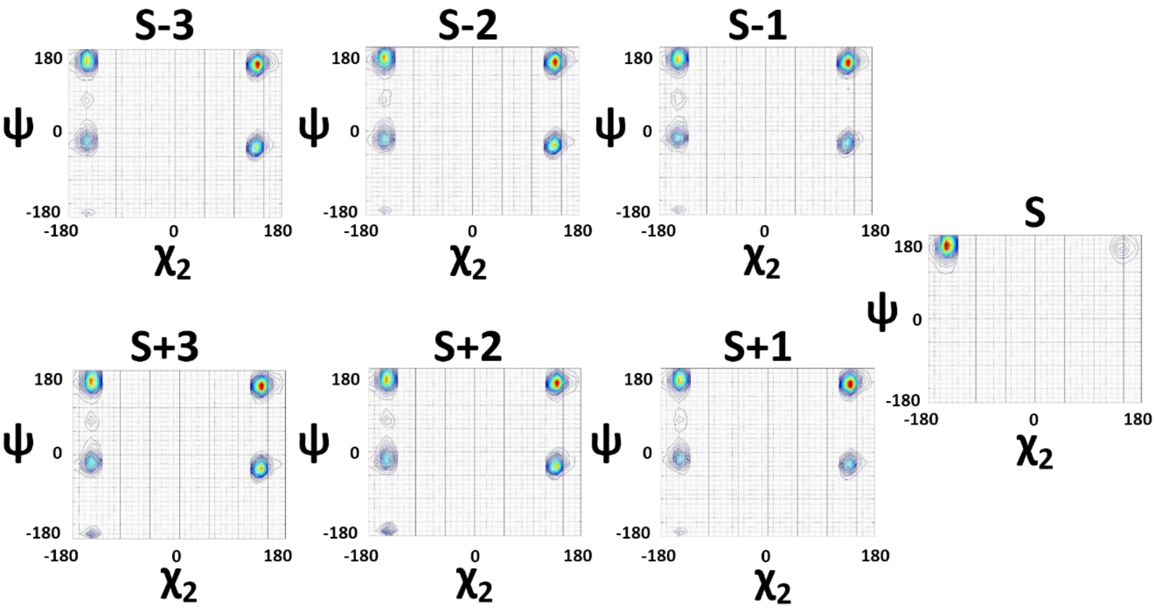

PRO

Fig A42: **Propensity of PRO in secondary structures.** : ψ vs χ_2 for helix (a) and sheets (b). The plots depict the most prominent in basins of localization for ψ and χ_2 dihedral rotors in PRO during secondary structure formation and breaking.

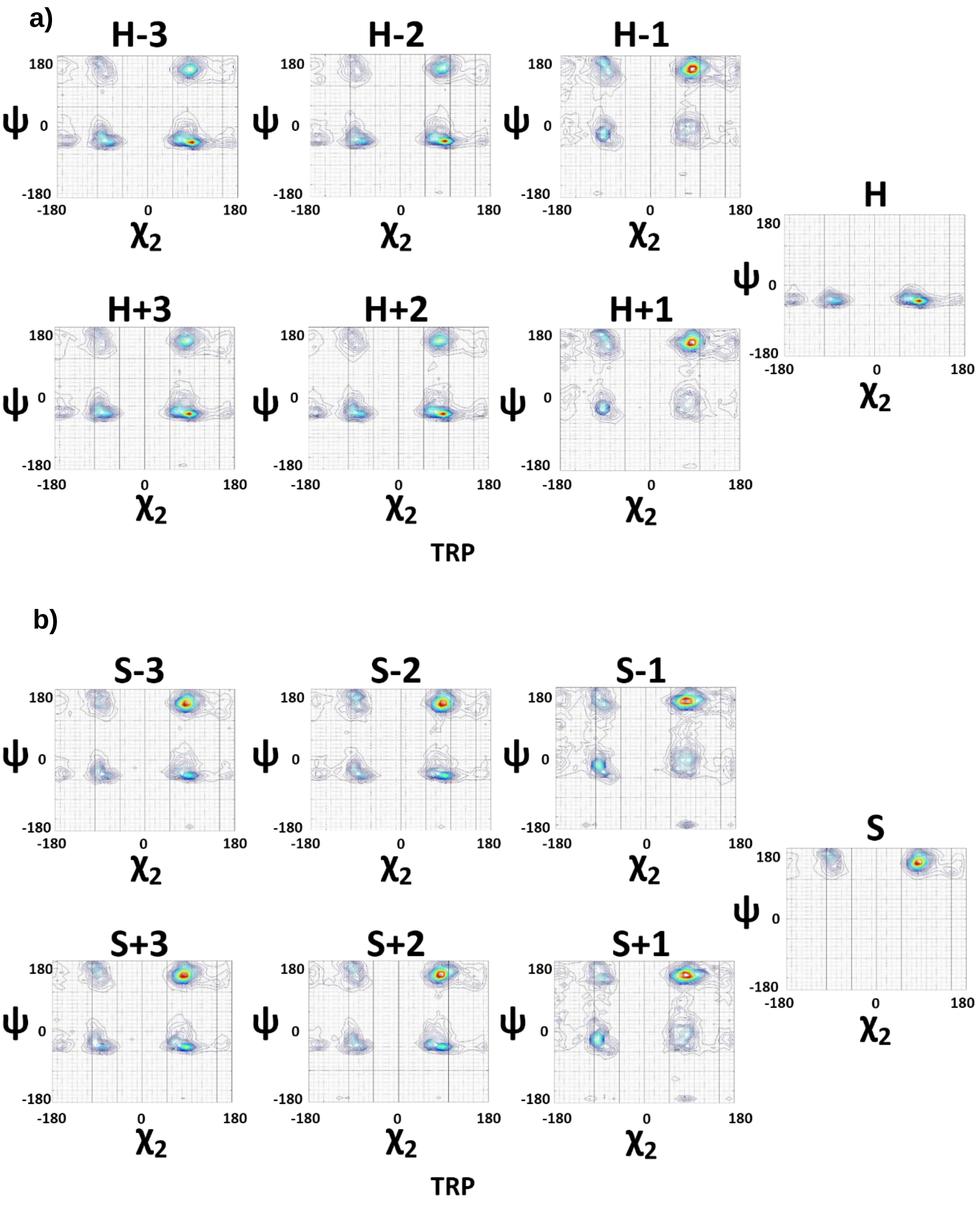

Fig A43: **Propensity of TRP in secondary structures.** : ψ vs χ_2 for helix (a) and sheets (b). The plots depict the most prominent in basins of localization for ψ and χ_2 dihedral rotors in TRP during secondary structure formation and breaking.

a)

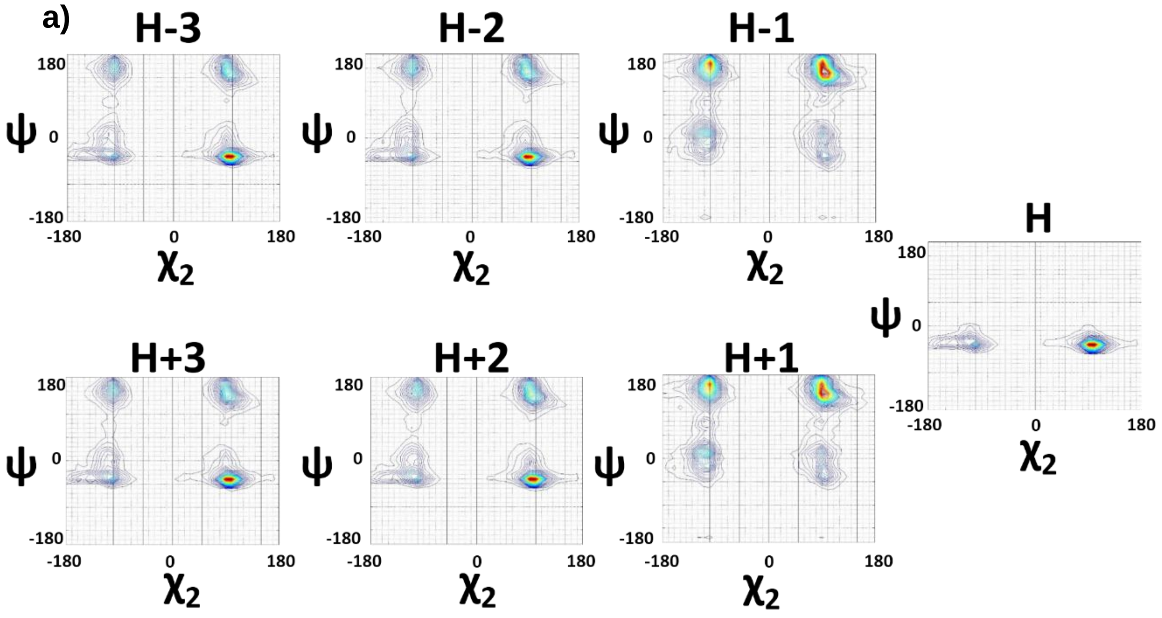

TYR

b)

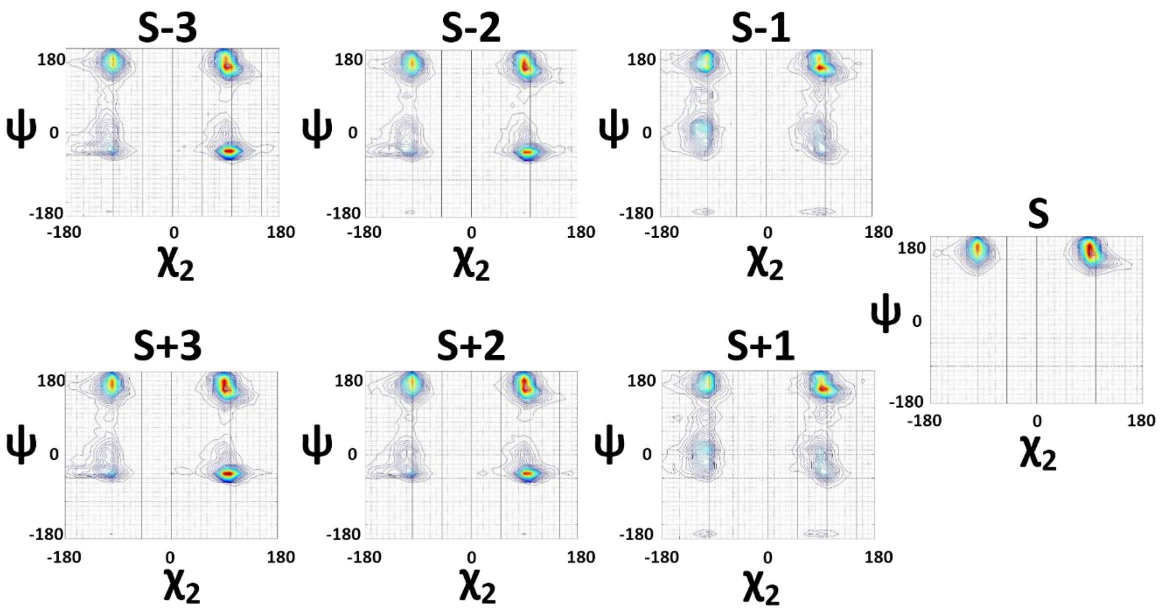

TYR

Fig A44: **Propensity of TYR in secondary structures.** : ψ vs χ_2 for helix (a) and sheets (b). The plots depict the most prominent in basins of localization for ψ and χ_2 dihedral rotors in TYR during secondary structure formation and breaking.
